# Supplementary material for: Truncating NFKB1 variants cause combined NLRP3 inflammasome activation and type I interferon signaling and predispose to necrotizing fasciitis
Source: Cell Rep Med. 2024 Apr 8;5(4):101503. doi: 10.1016/j.xcrm.2024.101503 (PMC11031424; doi:10.1016/j.xcrm.2024.101503)
Supplement: Document S1. Figures S1‒S4 and Tables S1–S5 [file mmc1.pdf]

Cell Reports Medicine, Volume *5*

Supplemental information

Truncating *NFKB1* variants cause combined NLRP3 inflammasome activation and type I interferon signaling and predispose to necrotizing fasciitis

Katariina Nurmi, Kristiina Silventoinen, Salla Keskitalo, Kristiina Rajamäki, Vesa-Petteri Kouri, Matias Kinnunen, Sami Jalil, Rocio Maldonado, Kirmo Wartiovaara, Elma Inés Nievas, Silvina Paola Denita-Juárez, Christopher J.A. Duncan, Outi Kuismin, Janna Saarela, Inka Romo, Timi Martelius, Jukka Parantainen, Arzu Beklen, Marcelina Bilicka, Sampsa Matikainen, Dan C. Nordström, Meri Kaustio, Ulla Wartiovaara-Kautto, Outi Kilpivaara, Christoph Klein, Fabian Hauck, Tiina Jahkola, Timo Hautala, Markku Varjosalo, Goncalo Barreto, Mikko R.J. Seppänen, and Kari K. Eklund

Figure S1

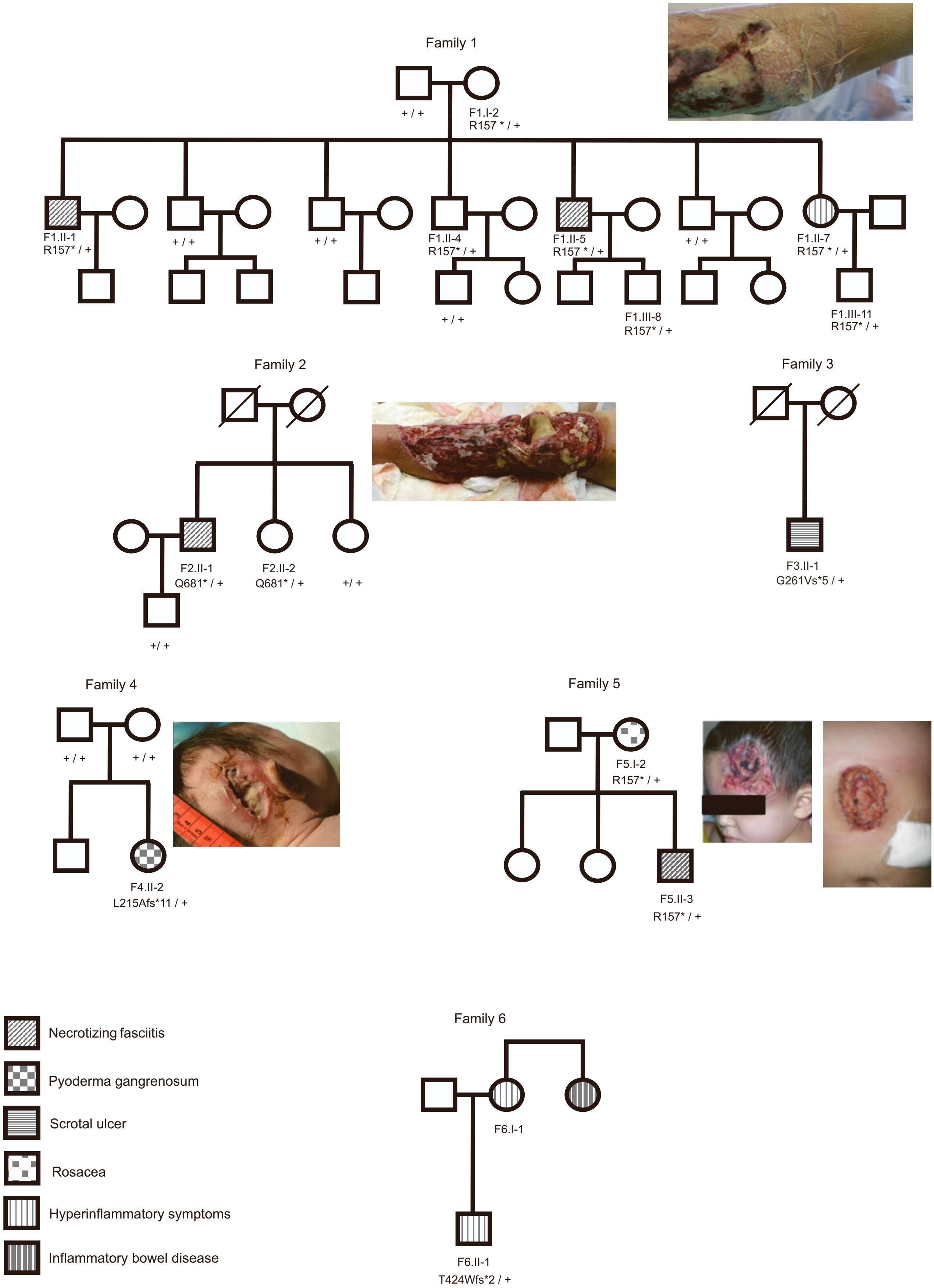
Family 1
+ / +
F1.I-2
R157 * / +
F1.II-1
R157* / +
+ / +
+ / +
F1.II-4
R157* / +
F1.II-5
R157 * / +
+ / +
F1.II-7
R157 * / +
+ / +
F1.III-8
R157* / +
F1.III-11
R157* / +
Family 2
F2.II-1
Q681* / +
F2.II-2
Q681* / +
+/ +
+/ +
Family 3
F3.II-1
G261Vs*5 / +
Family 4
+ / +
+ / +
F4.II-2
L215Afs*11 / +
Family 5
F5.I-2
R157* / +
F5.II-3
R157* / +
Family 6
F6.I-1
F6.II-1
T424Wfs*2 / +
Necrotizing fasciitis
Pyoderma gangrenosum
Scrotal ulcer
Rosacea
Hyperinflammatory symptoms
Inflammatory bowel disease

Figure S1. Pedigrees of *NFKB1* variant carriers and clinical manifestations in patients, related to Figure 1, Tables S1 and S2. Patients with clinical diagnosis are highlighted. +/+, absence of mutation; variant/+, mutation carrier. The clinical manifestation of patients: necrotizing fasciitis in patient F1.II-5; necrotizing fasciitis in patient F2.II-1 which led to leg amputation; retroauricular necrotizing tissue inflammation at the site of attachment of vacuum pump in the newborn (F4.II-2); necrotizing ocular cellulitis and postoperative pyoderma gangrenosum in abdomen in patient F5.II-3.

Figure S2

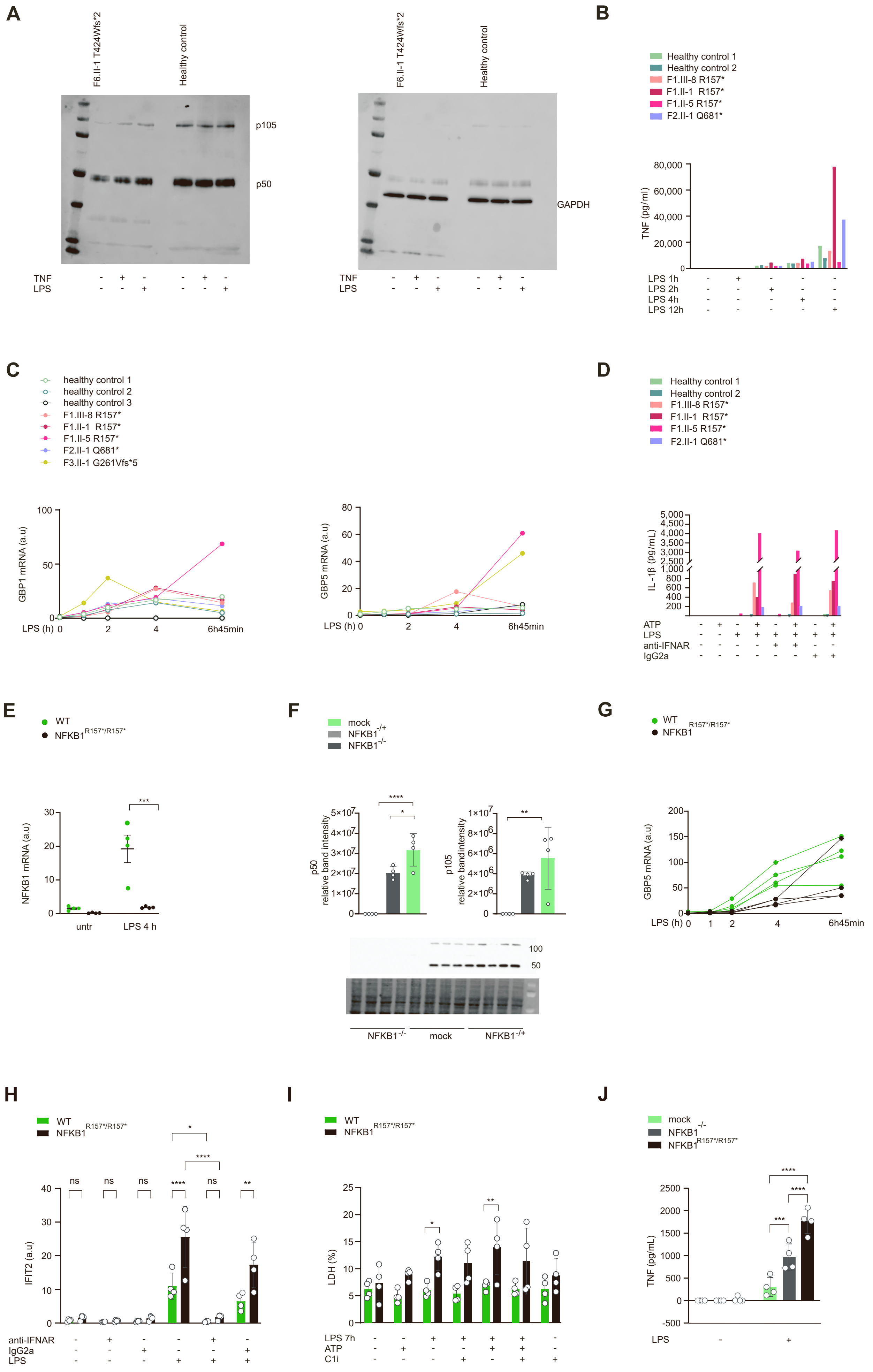
A
F6.II-1 T424Wfs*2
Healthy control
p105
p50
GAPDH
TNF
LPS
B
Healthy control 1
Healthy control 2
F1.III-8 R157*
F1.II-1 R157*
F1.II-5 R157*
F2.II-1 Q681*
TNF (pg/ml)
LPS 1h
LPS 2h
LPS 4h
LPS 12h
C
healthy control 1
healthy control 2
healthy control 3
F3.II-1 G261Vfs*5
GBP1 mRNA (a.u)
GBP5 mRNA (a.u)
LPS (h)
6h45min
D
IL -1β (pg/mL)
ATP
anti-IFNAR
IgG2a
E
WT
NFKB1 R157*/R157*
NFKB1 mRNA (a.u)
untr
LPS 4 h
F
mock
NFKB1 -/+
NFKB1 -/-
p50 relative band intensity
p105 relative bandintensity
G
GBP5 mRNA (a.u)
H
IFIT2 (a.u)
ns
I
LDH (%)
LPS 7h
C1i
J
TNF (pg/mL)

Figure S2. IFN-I signaling is required for enhanced expression of selected ISGs but has no effect on enhanced NLRP3 inflammasome activation, related to Figures 1 and 2.

(A) PBMCs derived from p.T424Wfs*2 variant carrier and age and gender matched healthy control were stimulated with LPS (10 ng/mL) and TNF (10 ng/mL) for 16 h and expression of p105/p50 and GAPDH were blotted. **(B)** HMDMs were activated with LPS (1 µg/ml) for indicated times and the secretion of TNF was detected from supernatants by ELISA. **(C)** HMDMs were activated with LPS (1 µg/ml) for indicated times and the expression of *GBP1* and *GBP5* was analyzed by RT-qPCR. **(D)** Anti-IFNAR2 antibody (5 µg/ml) or mouse anti-human IgG2a (mock, 5 µg/ml) were applied 30 min prior to activation of HMDMs with LPS (1 µg/ml, 6 h 45 min) followed by ATP (5 mM, for the last 45 min of the incubation). Mature IL-1β was detected from supernatants by ELISA. **(E)** *NFKB1*$^{R157*/ R157*}$ and WT THP-1 monocytes were activated with LPS (1 µg/ml, 4 h) and the expression of *NFKB1* was analyzed by RT-qPCR. 2-way ANOVA followed by Šidak’s multiple comparison test. **(F)** The protein expression of NF-κB1 was blotted from the whole cell lysates of untreated *NFKB1*$^{-/-}$ and *NFKB1*$^{-/+}$ and mock transfected THP-1 monocytes. Blot and total protein loading shown. 1-way ANOVA followed by Dunnett´s multiple comparison test. **(G)** *NFKB1*$^{R157*/ R157*}$ and WT THP-1 monocytes were activated with LPS (1 µg/ml) for indicated times and expression of *GBP5* was analyzed by RT-qPCR. 2-way ANOVA followed by Šidak’s multiple comparison test. **(H)** Anti-IFNAR2 antibody (5 µg/ml) or mouse anti human IgG2a (mock, 5 µg/ml) were applied 30 min prior to activation of *NFKB1*$^{R157*/ R157*}$ and WT THP-1 monocytes with LPS (1 µg/ml, 6 h 45 min), and the expression of *IFIT2* was detected by RT-qPCR. 2-way ANOVA followed by Šidak’s multiple comparison test. **(I)** *NFKB1*$^{R157*/ R157*}$ and WT THP-1 monocytes were stimulated with LPS (1 µg/ml, 6 h 45 min) and treated with caspase-1/4 inhibitor (Z-YVAD-FMK, 15 µM) 1 h before ATP (5 µM, for the last 45 min of the incubation). Secretion of LDH was assessed. 2-way ANOVA followed by Šidak’s multiple comparison test. **(J)** *NFKB1*$^{R157*/ R157*}$, *NFKB1* $^{-/-}$ and WT THP-1 monocytes were activated with LPS (1 µg/ml) for 6 h 45 min and secreted TNF was detected from supernatants by ELISA. 2-way ANOVA followed by Tukey’s multiple comparison test. The data is shown as mean ±SD. (A) 1 variant carrier, 1 control; (B) 4 variant carriers, 2 controls; (C) 5 variant carriers, 3 controls; (D) 4 variant carriers, 2 controls; (E-J) THP-1 n=4.

Figure S3

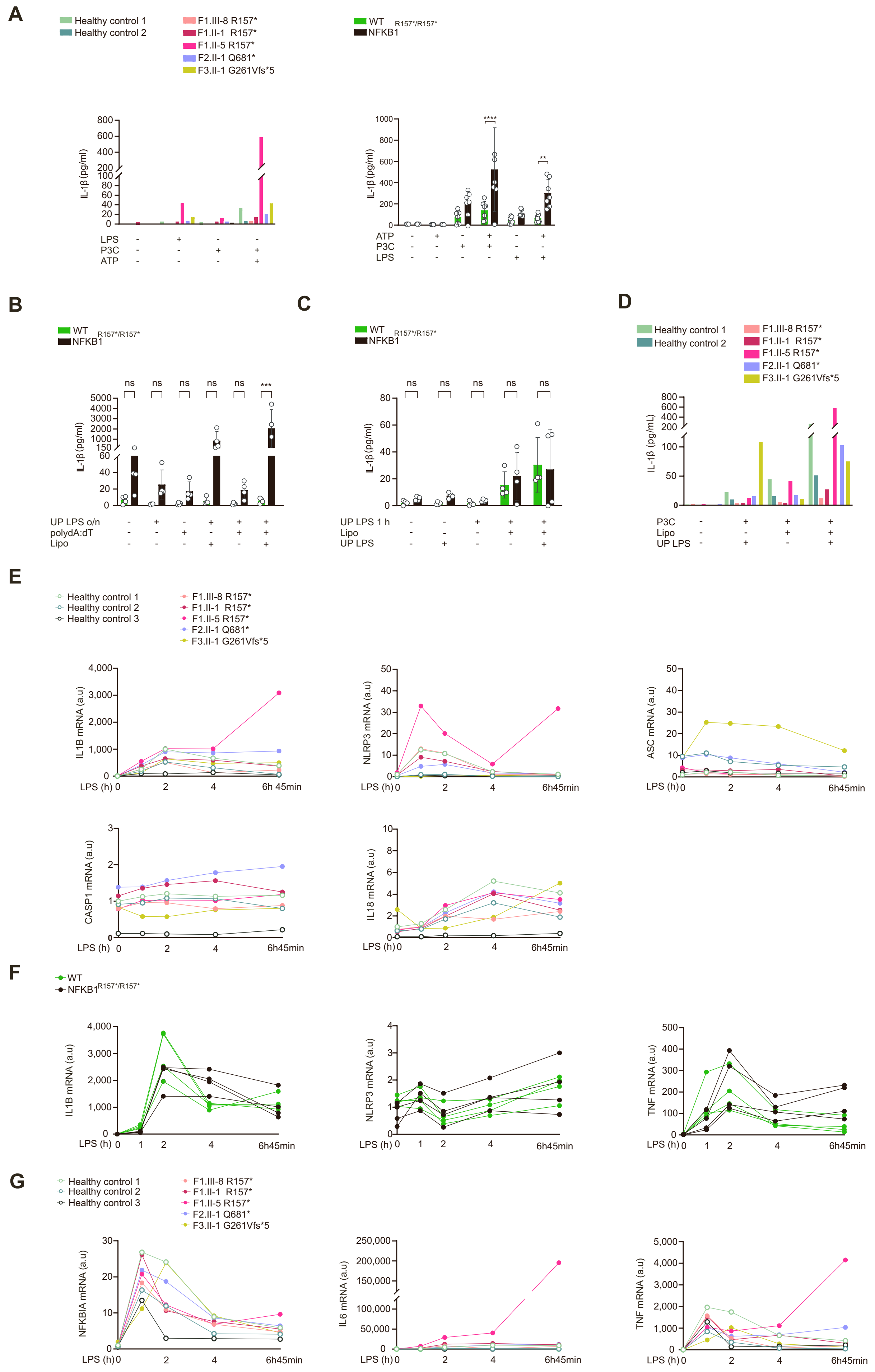
A
Healthy control 1
Healthy control 2
F1.III-8 R157*
F1.II-1 R157*
F1.II-5 R157*
F2.II-1 Q681*
F3.II-1 G261Vfs*5
WT
NFKB1 R157*/R157*
IL-1β (pg/ml)
LPS
P3C
ATP
B
UP LPS o/n
polydA:dT
Lipo
C
UP LPS 1 h
Lipo
UP LPS
D
IL-1β (pg/mL)
E
IL1B mRNA (a.u)
NLRP3 mRNA (a.u)
ASC mRNA (a.u)
CASP1 mRNA (a.u)
IL18 mRNA (a.u)
Healthy control 3
LPS (h)
6h45min
F
TNF mRNA (a.u)
G
NFKBIA mRNA (a.u)
IL6 mRNA (a.u)

Figure S3. *NFKB1* variants increase AIM2 inflammasome activation but have no effect on activation of the noncanonical inflammasome nor expression of NF-κB-regulated genes, related to Figures 2 and 4. (A) HMDMs (left panel) and *NFKB1*$^{R157*/ R157*}$ and WT THP-1 monocytes (right panel) were primed with Pam3Cys-SKKKK (1 µg/ml, 6 h 45 min) or LPS (1 µg/ml, 6h 45 min), followed by ATP (5 mM, for the last 45 min of the incubation). Mature IL-1β was detected from supernatants by ELISA. Statistical analysis by 2-way ANOVA followed by Šidak's multiple comparison test. **(B)** *NFKB1*$^{R157*/ R157*}$ and WT THP-1 monocytes were primed o/n with ultrapure LPS (UP-LPS, 1 µg/mL) and transfected with dA:dT (0,2 µg/ml, 5 h) or **(C)** primed with UP-LPS (1 µg/mL, 1 h) and transfected with UP-LPS (1 µg/mL, 5 h). IL-1β secretion was detected from the supernatants by ELISA. 2-way ANOVA followed by Šidak's multiple comparison test. **(D)** HMDMs were primed with Pam3Cys-SKKKK (1 µg/ml, 6 h) after which the cells were transfected with UP-LPS (1 µg/mL, 5 h). IL-1β secretion was detected from the supernatants by ELISA. **(E)** HMDMs were activated with LPS (1 µg/ml) for indicated times and the expression of inflammasome components *IL1B*, *NLRP3, ASC*, *CASP1*, and *IL18* was analyzed by RT-qPCR. **(F)** *NFKB1*$^{R157*/ R157*}$ and WT THP-1 monocytes were activated with LPS (1 µg/ml) for indicated times and expression of *IL1B*, *NLRP3*, and *TNF* was analyzed by RT-qPCR. Statistical analysis by 2-way ANOVA followed by Šidak's multiple comparison test. **(G)** HMDMs were activated with LPS (1 µg/ml) for indicated times and the expression of *NFKBIA*, *IL6*, and *TNF* was analyzed by RT-qPCR. The data is shown as mean ±SD. (A) HMDM 5 variant carriers, 2 controls; THP-1 n=4; (B-C) n=4, (D) 5 variant carriers, 2 controls; (E, G) 5 variant carriers, 3 controls; (F) n=4.

Figure S4

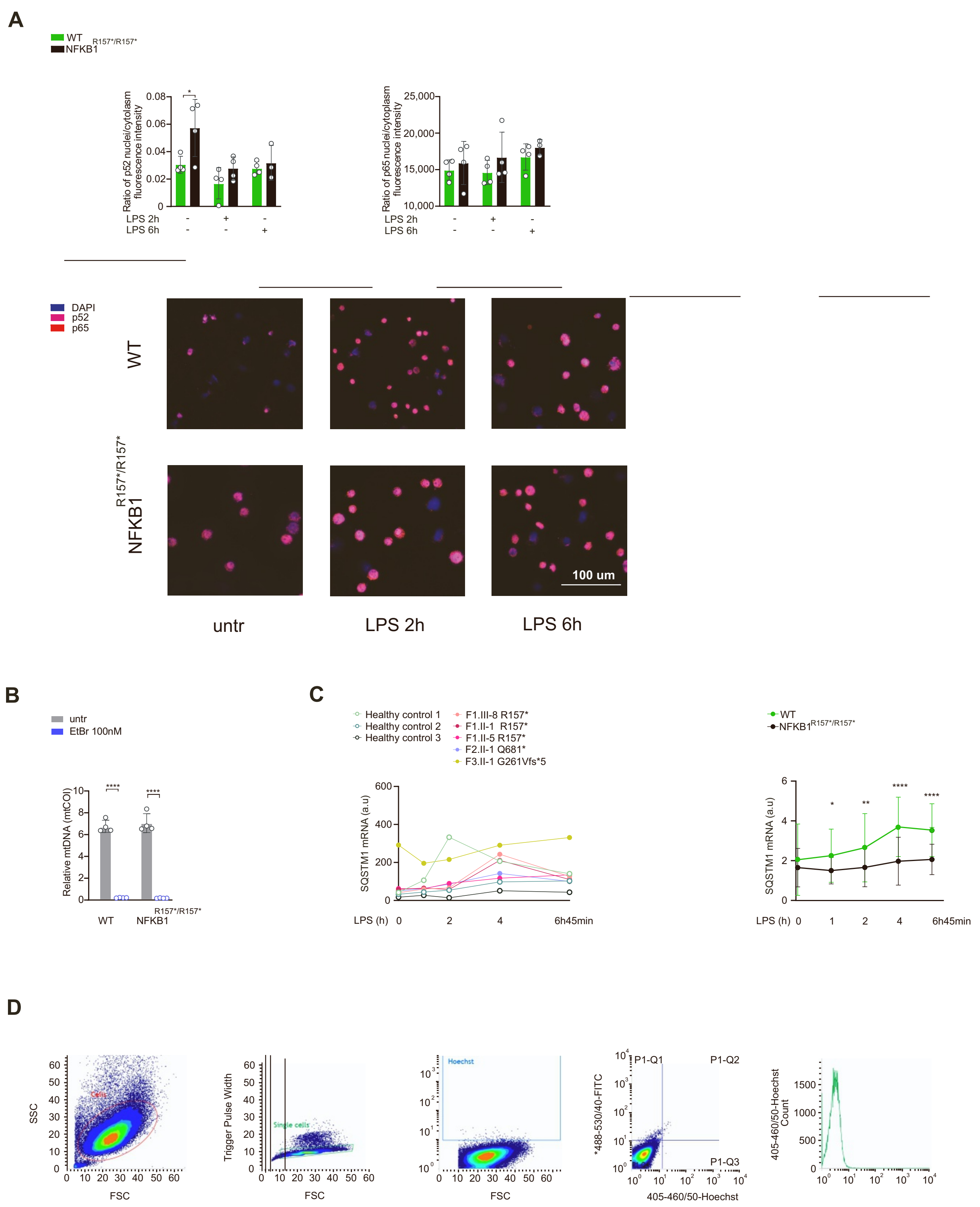
A
WT
NFKB1 R157*/R157*
Ratio of p52 nuclei/cytolasm fluorescence intensity
Ratio of p65 nuclei/cytoplasm fluorescence intensity
LPS 2h
LPS 6h
DAPI
p52
p65
WT
NFKB1 R157*/R157*
100 um
untr
LPS 2h
LPS 6h
B
untr
EtBr 100nM
Relative mtDNA (mtCOI)
WT
NFKB1 R157*/R157*
C
Healthy control 1
Healthy control 2
Healthy control 3
F1.III-8 R157*
F1.II-1 R157*
F1.II-5 R157*
F2.II-1 Q681*
F3.II-1 G261Vfs*5
SQSTM1 mRNA (a.u)
LPS (h)
6h45min
D
SSC
FSC
Trigger Pulse Width
Single cells
Hoechst
P1-Q1
P1-Q2
P1-Q3
*488-530/40-FITC
405-460/50-Hoechst
405-460/50-Hoechst Count

Figure S4. ***NFKB1*** **variants increase nuclear location of p52 but have no effect on nuclear location of p65, related to Figures 4 and 5. (A)** *NFKB1*$^{R157*/ R157*}$ and WT THP-1 monocytes were activated with LPS (1 µg/mL) for indicated times, and ratios of nuclear-to-cytoplasmic staining for p52 and p65 were determined. Scale bar 100 µm. The data is shown as mean ±SD. **(B)** Total DNA was extracted from resting ρ^0 and conventionally cultured *NFKB1*$^{R157*/ R157*}$ and WT THP-1 monocytes, and levels of mitochondrial DNA were assessed by analyzing the level of mtCOI DNA by RT-qPCR. **(C)** HMDMs (left) and *NFKB1*$^{R157*/ R157*}$ and WT THP-1 (right) monocytes were activated with LPS (1 µg/ml) for indicated times and the expression of *SQSTM1* was analyzed by RT-qPCR. **(D)** Autophagic vesicle detection strategy by flow cytometry (untreated cells depicted). Single cells positive for Hoechst and *488-530/40-FITC were gated. (A-C) 2-way ANOVA followed by Šidak's multiple comparison test. The data is shown as mean ±SD. (A-B) n=4; (C) HMDM 5 variant carriers, 3 controls; THP-1 n=8.

Table S1, *NFKB1* variant carrier information, related to STAR Methods and Figure S1

Family	Family 1	Family 2	Family 3	Family 4	Family 5	Family 6
**Variant carrier**	F1.II-1<br>F1.II-5<br>F1.III-8	F2.II-1	F3.II-1	F4.II-2	F5.II-3	F6.II-1
**Gender**	M, M, M	M	M	F	M	M
**Age of onset**	48 / 28 / N.A.	63	45	newborn	2	30
**Ancestry**	European (Finland)	European (Finland)	European (Finland)	European (Germany)	Latin American (Argentina)	European (UK)
**Genomic variant (GRCh37)**	chr4:103498094, C>T [1]	chr4:103528393, C>T [1]	chr4:103501740, insCTGTC [1]	chr4:103500102 G>GGTGC [1]	chr4:103498094, C>T [1]	Chr4:103516108, delAA [1]
**cDNA change (NM_003998.3)**	c.469C>T	c.2041C>T	c.781_782insTCCTG	c.638_641dup	c.469C>T	c.1269_1270delAA
**Amino acid change**	p.R157*	p.Q681*	p.G261Vfs*5	p.L215Afs*11	p.R157*	p.T424Wfs*2
**Mutation type**	Truncating	Truncating	Truncating	Truncating	Truncating	Truncating
**Inheritance**	AD	AD	AD	*De novo*	AD	AD
**Exon**	7	18	9	8	7	13
**Location of NF or severe tissue inflammation**	(II-1) limb<br>(II-5) limb<br>(III-8) N/A	limb	scrotum	umbilicus, retroaulicular region	orbital region, abdomen	Sternocleido-mastoid muscle
**Blood count**		recurrent leucocytosis	recurrent leucopenia, neutropenia thrombocytopenia, low NK cells	N.A.	N.A.	microcytic anemia, monocytosis, low grade eosinophilia
**Low plasma immunoglobulin**	(II-1) IgG, IgM	-	IgM, IgA, IgG		-	-
**Febrile attacks**	-	-	-	-	x	x
**Pyoderma gangrenosum**	-	-	-	x	x	-
**Other genomic variants with IEI association[2]/ gene name/ variant type/ gnomad freq/ clinical interpretation**	Chr7:15601422,G>A/ AGMO/ nonsynonymous SNV/ 0,0001779/ Likely not significant;<br>Chr16:69727480,A>G/ NFAT5/ nonsynonymous SNV/ 0,00371/ Not significant;<br>Chr1:247587425,C>T/ NLRP3/ nonsynonymous SNV/ 0,0003856/ Likely not significant	Chr1:93303114,A>G/ RPL5/ nonsynonymous SNV/ 0,004748/ Not significant;<br>Chr12:110034320,G>A/ MVK/ nonsynonymous SNV/ 0,001578/ Not significant (as heterozygous);<br>Chr2:48018236,G>T/ MSH6/ nonsynonymous SNV/ 0,0009757/ Not significant;<br>Chr22:37261097,C>A/ NCF4/ nonsynonymous SNV/ 0,00243/ Not significant;<br>Chr8:61693942,->AAAGCA/ CHD7/ nonframeshift insertion/ 0,0062/ Not significant	No other pathogenic variants fitting to phenotype were found	No other pathogenic variants fitting to phenotype were found	Chr1:154574541, C>G/ ADAR/ nonsynonymous SNV/0,00221/conflicting	No other pathogenic variants fitting to phenotype were found
**Other**	N.A.	N.A.	oral aftosis, follicular hyperplasia of lymph nodes, splenomegaly, epiglottitis	N.A.	N.A.	splenomegaly, generalised lymphadenopathy

[1] Variant is not found in GnomAD; [2] all variants are heterozygous.

Table S2 Clinical features of the affected *NFKB1* variant carriers, related to STAR Methods and Figure S1

**Variant carrier**	**F1.II-1**	**F1.II-5**	**F1.III-8**	**F2.II-1**	**F3.II-1**	**F4.II-2**	**F5.II-3**	**F6.II-1**
**Variant**	R157*	R157*	R157*	p.Q681*	p.G261Vfs*5	p.L215Afs*11	p.R157*	p.T424Wfs*2
**Auto-antibodies**	n.a.	n.a.	n.a.	No ANCA antibodies<br>ANA titers: 1) 80; 2) <80<br>No RF or BP180 ab (Pemfigoid)<br>No other skin-directed antibodies (interstitium, basement membrane)	No ANA, no RF	ANA 1:200, no ANCA, no RF	No ANA or ANCA autoantibodies	mild elevation of ANA (40-80) without other autoantibodies
**CRP/ESR during inflammatory episode**	CRP 281 mg/mL	CRP 433 mg/L	n.a.	CRP 352 mg/L	n.a.	CRP 197 mg/L	Pneumonia (age 14 yrs) P-CRP 333 mg /L	Persistently elevated CRP (20-60 mg/L) and ESR (40-70)
**WBC/neutrophils during inflammatory episode**	WBC 46.6 x 10^9 cells/L	WBC 66.8 x 10^9 cells/L	n.a.	33.7-56.7 x 10^9 /L	n.a.	WBC 25.1 x 10^9 / L, neutrophils 13.8 x 10^9 /L	WBC 47 x 10^9 cells /L	WBC 21.81 x 10^9 /L Neutrophils 14.3 x 10^9 /L
**Bacterial cultures during inflammatory episode**	No bacterial or fungal pathogens found in blood cultures or at surgical site.	No bacterial or fungal pathogens found in blood cultures or at surgical site.	n.a.	Blood (n=16), deep tissue cultures, bacterial PCR and stains negative, superficial cultures grew low counts of *Staphylococcus aureus* and S.*epidermidis*	n.a.	No bacterial or fungal pathogens found in blood cultures (every 48-72 h) or in the retroauricular area after P2.	No anaerobic or acid fast bacteria in ulcer culture or intra-abdominal liquid	No bacterial or fungal pathogens found in blood cultures or at surgical site.
**Vaccine responses**	n.a.	n.a.	Response to Pneumovax 4/10	n.a.	Impaired anti-PnP	Normal responses against protein and attenuated viruses	Normal against tetanus toxoid Ab and pneumococcal vaccine	Normal responses to HiB and pneumococcal vaccine
**Neutrophil oxidative burst**	Normal (Ref. 22)	Normal (Ref. 22)	n.a.	n.a.	n.a.	Normal/slightly increased	Normal	n.a.
**CD62L shedding**	n.a.	n.a.	n.a.	n.a.	n.a.	Normal/slightly increased	n.a.	n.a.
**Other**	Slightly reduced marginal zone ($CD27^+IgM^+IgD^+$) and class-switched B-cells ($CD27^+IgM^-IgD^-$) [1]	Slightly reduced class-switched B-cells ($CD27^+IgM^-IgD^-$) [1]	Slightly reduced class-switched B-cells ($CD27^+IgM^-IgD^-$) [1]	n.a.	Splenomeghaly, hypogammaglobulinemia, follicular hyperplasia, epiglottitis (x 2), Scrotal ulcers, complex aftaeae, constant moderate thrombocytopenia and mild neutropenia [2]	Normal/slightly increased expression of CD11/CD18	Slightly reduced class-switched B-cells ($CD27^+IgM^-IgD^-$) and memory B cells ($CD19^+ CD27^+$)	No class-switched memory B cells (CD27+IgM-IgD-)

Abbreviations: ANA, antinuclear antibody; ANCA, antineutrophil cytoplasmic antibodies; anti-PnP, anti-pneumococcal polysaccharide.

1. Santaniemi, W., Astrom, P., Glumoff, V., Pernaa, N., Tallgren, E.N., Palosaari, S., Nissinen, A., Kaustio, M., Kuismin, O., Saarela, J., et al. (2023). Inflammation and Neutrophil Oxidative Burst in a Family with NFKB1 p.R157X LOF and Sterile Necrotizing Fasciitis. J Clin Immunol *43*, 1007-1018. 10.1007/s10875-023-01461-3.
2. Tuovinen, E.A., Kuismin, O., Soikkonen, L., Martelius, T., Kaustio, M., Hamalainen, S., Viskari, H., Syrjanen, J., Wartiovaara-Kautto, U., Eklund, K.K., et al. (2023). Long-term follow up of families with pathogenic NFKB1 variants reveals incomplete penetrance and frequent inflammatory sequelae. Clin Immunol *246*, 109181. 10.1016/j.clim.2022.109181.

Table S3. BioID interactome data of NF-κB1 WT and NF-κB1 variants, related to Figure 3.

Averages of affinity purification mass spectrometry streptavidin-hemagglutinin (Strep-HA)-tag normalized log2 transformed interactor values.

Interactor	R157*	G261Vfs*5	Q681*	WT
human NFKB1	2,482	3,867	20,778	25,691
human NFKB2	N.A.	N.A.	5,772	5,336
human TF65	N.A.	N.A.	3,919	3,708
human REL	N.A.	N.A.	2,386	2,155
human RELB	N.A.	N.A.	2,031	1,538
human IKBA	N.A.	N.A.	1,325	0,739
human IKBB	N.A.	N.A.	3,122	2,065
human IKBE	N.A.	N.A.	1,369	1,144
human TNIP2	N.A.	N.A.	1,292	1,098
human TNIP1	N.A.	N.A.	N.A.	0,503
human M3K8	N.A.	N.A.	0,397	0,436
human M1IP1	N.A.	N.A.	0,264	N.A.
human FETUA	0,339	N.A.	N.A.	N.A.
human HACL2	N.A.	N.A.	N.A.	0,573
human FLOT1	N.A.	N.A.	N.A.	0,314

Table S4. Nanostring gene expression data of *NFKB1* variant carriers and healthy controls, related to Figure 4.

Nanostring gene expression raw data, mean of triplicate scans.

Gene	Healthy control	Healthy control	Healthy control	F1.II-1	F1.II-5	F2.II-1	F3.II-1	F4.II-2
CASP1	12436	12694	7832	16040	11190	6763	8036	4228
CASP5	175	199	217	292	144	260	141	146
CASP8	7724	9199	13107	7202	7186	12301	9591	35101
CXCL10	144	163	71	276	80	84	171	44
CXCL9	121	55	71	125	84	154	25	44
IDO1	186	118	71	118	85	67	25	44
IFI27	80	55	76	926	76	67	66	44
IFI44	1625	1804	1911	9528	1966	1886	2768	3114
IFI44L	1640	974	1388	19714	1655	2204	3225	1543
IFI6	603	471	694	2900	808	716	778	645
IFIH1	1017	1055	1691	1820	841	1637	1323	2311
IFIT1	279	390	135	2044	353	311	429	154
IFIT2	1574	353	310	2838	888	515	674	454
IFIT3	160	131	101	793	146	223	117	44
IFNA1	80	55	71	102	76	67	25	44
IFNA2	80	55	71	102	76	67	25	44
IFNB1	80	55	71	102	76	67	25	44
IFNG	220	411	187	508	126	579	169	44
IL18	204	320	394	538	320	405	183	262
IL1A	261	332	170	324	335	124	25	86
IL1B	10709	13652	6368	16767	17297	4895	832	2251
IL6	139	85	281	308	201	231	113	270
ISG15	2348	1620	1863	5319	2131	1809	1953	1215
JAK1	9162	7484	12413	7698	9105	9747	7817	19474
JAK2	5854	5378	5569	10913	5178	5513	6102	4929
JAK3	2275	2924	3502	2229	1958	4803	4960	6861
MX1	3504	3540	4311	19434	5747	6852	7049	6648
NFKB1	1702	1671	1983	1271	1076	1309	1347	3310
NFKB2	2220	2087	7367	2571	1814	5580	2633	13892
NFKBIA	59024	100544	94648	48068	50819	48648	33869	130706
NLRP12	1251	1366	1791	1826	1520	1222	1518	703
NLRP3	3908	3384	5985	4359	4247	5258	2115	7794
OAS1	3755	3822	2609	8276	3106	2556	4061	924
REL	4387	2482	5862	4205	4035	2792	1838	8792
RELA	3683	3363	3508	3302	2987	2972	2645	5054
RELB	1345	905	1943	1122	1121	1198	1266	7061
RSAD2	465	397	340	2709	421	592	711	453
SIGLEC1	226	219	258	5088	305	374	297	196
STAT1	13836	10215	12062	27343	8164	15033	19665	11025
STAT2	1433	1269	1586	2850	1335	1799	2056	1667
STAT3	5876	5101	7759	7221	5967	8044	6588	12998
STAT4	2544	3136	4893	2634	2477	4535	3305	14165
STAT5B	4003	4384	4607	3727	3514	5021	4863	7612
STAT6	6299	6435	14496	8653	7100	16839	10578	14103
USP18	104	115	114	465	111	154	180	174
EEF1G	59729	62326	81358	49047	59706	93062	79289	114768
GAPDH	43788	39829	38315	47927	50881	34234	42730	35782
HPRT1	3038	2892	3190	2910	2857	2923	2615	3933
OAZ1	42929	42178	42991	48993	49551	46007	48330	30167
TUBB	3111	3505	2482	3166	2467	2477	2478	2178

Table S5. Primers used in RT-PCR and PCR analysis, and guides for CRISPR-Cas9 editing, related to STAR Methods.

RT-qPCR primers

Gene	Forward	Reverse
human A20	CTGGGACCATGGCACAACTC	TCGCTGTTTTCCTGCCATTTC
human ASC	TTGGACCTCACCGACAAGC	ATGTCGCGCAGCACGTTA
human CASP1	ATCCCACAATGGGCTCTGTTT	CTCTTTCAGTGGTGGGCATCT
human CASP4	TTGAAAATGGAAGCCACAAGCA	CAAGCTGTACTAATGAAGGTGCTC
human CASP5	CTTCAAGGCCTGGGCTACAC	TCAGCACTGACTCCATATCCCTG
human CXCL10	GCAAGCCAATTTTGTCCACGTGTTG	CAGCCTCTGTGTGGTCCATCCTT
human GBP1	GGAGATTGAAGTGGAACGTGTGA	TCTGCTTTCTTTTTGAAATCCCTCT
human GBP5	GTACTATCGGGAGCCTCGGA	TTTGAATCGCCGCCAACCTT
human IL10	TACGGCGCTGTCATCGATTT	TAGAGTCGCCACCCTGATGT
human IL18	TCAACTCTCTCCTGTGAGAACAAA	GTCCTGGGACACTTCTCTGAAA
human mtCOI1	CGATGGTGCAGCCGCTATTA	ATCATTTACGGGGGAAGGCG
human NFKBIA	CGGACTGCCCTTCACCTCGC	GTATCCGGGTGCTTGGGCGG
human NLRP3	CAACTGCAACCTCACGTCAC	ACGGTCAGCTCAGGCTTTTC
human RPLP0	GAAATCCTGAGTGATGTGCAGC	TCGAACACCTGCTGGATGAC
human SQSTM1	TCGGAGGATCCGAGTGTGAAT	TTTCCCTCCGTGCTCCACAT

PCR primers

Gene name	Forward primer	Reverse primer	Melting temperature
NFKB1 R157*/R157*	ACCCGGTAAGACTTCCTCAT	TGGACCCCTTGGAGGATCAA	60 °C
NFKB1 -/- Exon 7	TGCTCCCACTCCCGTAATCAAA	TGGAAACGGTGGGGTGTTTTTG	62 °C

Off target PCR primers

Off target	Forward	Reverse	Score
1	AGGGGAGATGCTTAGCGTGTTT	AAGGCTTGAGAGAGGAGCACAG	0,88
2	GCGACATTTGCTCATCCTCGAG	TCACTGCGTCTCCTTTCCAACT	0,88
3	TATGGTTGGAGCTGGATCGCAA	CCTTGGTTTGTCACTTATATGCCCC	0,87
4	AGGCGTACAAGGTCAGTGCTTC	CCAGGACAGAAATCAATGCGCT	0,85
5	CCCCAGCTGTAACTGATGGACA	AAGCTACACATGGCTCCAGGTT	0,81

CRISPR-Cas9 guide RNA & HDR donor

<table>
<tr><th>Gene name</th><th>HDR donor template</th><th colspan="2">sgRNA</th></tr>
<tr><td>NFKB1 R157*/R157*</td><td>TGTTGTTGCTGCTGCTGTTACTGTTTTTTCTCCAGCTTCGCAAACCTGGGTATACTTCATGTGACAAAGAAAAAAGTATTTGAAACTCTGGAAGCATGAATGACTGAAGCGTGTATAAGGGGCTATAATCCTGGACTCTTGGTGCACCCTGACCTTGCCTATTTGCAAGCAGAAGGTGGAGGGGACCGGCAGCTGGGAGG</td><td colspan="2">ACTGGAAGCACGAATGACAG</td></tr>
<tr><th>Gene name</th><th></th><th>sgRNA1</th><th>sgRNA2</th></tr>
<tr><td>NFKB1 -/-
Exon 7</td><td></td><td>GAGATTTGTATAAAGCATTG</td><td>GATGAGGCTGAAAGTTGCTG</td></tr>
</table>
