# Supplementary material for: Truncating NFKB1 variants cause combined NLRP3 inflammasome activation and type I interferon signaling and predispose to necrotizing fasciitis
Source: Cell Rep Med. 2024 Apr 8;5(4):101503. doi: 10.1016/j.xcrm.2024.101503 (PMC11031424; doi:10.1016/j.xcrm.2024.101503)
Supplement: Document S2. Article plus supplemental information [file mmc4.pdf]

Cell Reports
Medicine

Article

Truncating *NFKB1* variants cause combined NLRP3 inflammasome activation and type I interferon signaling and predispose to necrotizing fasciitis

Graphical abstract

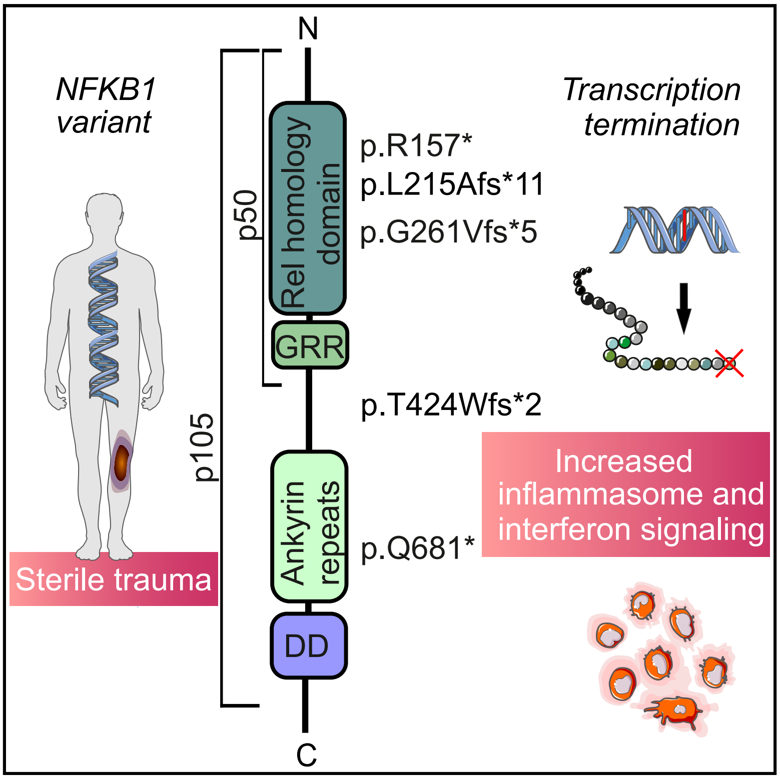

Highlights

- Patients carrying truncating *NFKB1* variants present with hyperinflammatory symptoms
- Truncation of *NFKB1* enhances inflammasome activation and type I interferon response
- Truncation of *NFKB1* impairs autophagic degradation
- Impaired autophagy leads to accumulation of NLRP3 inflammasome components and TRIF

Authors

Katariina Nurmi, Kristiina Silventoinen, Salla Keskitalo, ..., Goncalo Barreto, Mikko R.J. Seppänen, Kari K. Eklund

Correspondence

mikko.seppanen@hus.fi (M.R.J.S.), kari.eklund@hus.fi (K.K.E.)

In brief

Patients presenting with hyperinflammatory reactions to minor trauma or infection may carry predisposing genetic variants. Nurmi and Silventoinen et al. identify a mechanism of autoinflammation in which truncating variants of NF-κB1 impair autophagic degradation. Failure of autophagy increases inflammasome activation and interferon signaling, thus leading to hyperinflammation.

Nurmi et al., 2024, Cell Reports Medicine *5*, 101503
April 16, 2024 © 2024 The Authors. Published by Elsevier Inc.
https://doi.org/10.1016/j.xcrm.2024.101503

CellPress

Cell Reports Medicine

CellPress
OPEN ACCESS

Article

Truncating *NFKB1* variants cause combined NLRP3 inflammasome activation and type I interferon signaling and predispose to necrotizing fasciitis

Katariina Nurmi,[1,27] Kristiina Silventoinen,[1,27] Salla Keskitalo,[2] Kristiina Rajamäki,[1,3] Vesa-Petteri Kouri,[1] Matias Kinnunen,[2] Sami Jalil,[4] Rocio Maldonado,[4] Kirmo Wartiovaara,[4] Elma Inés Nievas,[5] Silvina Paola Denita-Juárez,[6] Christopher J.A. Duncan,[7] Outi Kuismin,[8,9] Janna Saarela,[10,11,12] Inka Romo,[13] Timi Martelius,[13] Jukka Parantainen,[1]

(Author list continued on next page)

[1]Faculty of Medicine, Clinicum, Translational Immunology Research Program, Research Program Unit (RPU), University of Helsinki (UH), 00014 Helsinki, Finland
[2]Systems Biology/Pathology Research Group, iCAN Digital Precision Cancer Medicine Flagship, Institute of Biotechnology, HiLIFE, UH, 00014 Helsinki, Finland
[3]Department of Medical and Clinical Genetics, Applied Tumor Genomics Research Program, RPU, UH, 00014 Helsinki, Finland
[4]Clinical Genetics UH and Helsinki University Hospital (HUH), 00014 Helsinki, Finland
[5]Alexander Fleming Hospital, OSEP, CP 5501 Mendoza, Argentina
[6]HEMA, Laboratory for Clinical Genetics, CP 5501 Mendoza, Argentina
[7]Translational and Clinical Research Institute, Newcastle University, Newcastle upon Tyne NE1 4HH, UK
[8]Department of Clinical Genetics, Oulu University Hospital (OUH), 90014 Oulu, Finland
[9]PEDEGO Research Unit and Medical Research Center Oulu, OUH and University of Oulu (OU), 90014 Oulu, Finland
[10]Institute for Molecular Medicine Finland, HiLIFE, UH, 00014 Helsinki, Finland
[11]Centre for Molecular Medicine Norway, University of Oslo, 0313 Oslo, Norway
[12]Department of Medical Genetics, Oslo University Hospital, 0450 Oslo, Norway
[13]Inflammation Center, Department of Infectious Disease, HUH, 00029 Helsinki, Finland
[14]Department of Internal Medicine and Rehabilitation, HUH and UH, 00029 Helsinki, Finland
[15]Department of Hematology, HUH, Comprehensive Cancer Center, UH, 00029 Helsinki, Finland
[16]Applied Tumor Genomics Research Program, RPU, Faculty of Medicine, UH, 00014 Helsinki, Finland
[17]Department of Medical and Clinical Genetics/Medicum, Faculty of Medicine, UH, 00014 Helsinki, Finland
[18]iCAN Digital Precision Cancer Medicine Flagship, UH, 00014 Helsinki, Finland
[19]HUS Diagnostic Center, HUSLAB Laboratory of Genetics, HUH, 00029 Helsinki, Finland
[20]Department of Pediatrics, Dr. von Hauner Children's Hospital, University Hospital, Ludwig-Maximilians-Universität München, 80337 Munich, Germany

(Affiliations continued on next page)

SUMMARY

In monogenic autoinflammatory diseases, mutations in genes regulating innate immune responses often lead to uncontrolled activation of inflammasome pathways or the type I interferon (IFN-I) response. We describe a mechanism of autoinflammation potentially predisposing patients to life-threatening necrotizing soft tissue inflammation. Six unrelated families are identified in which affected members present with necrotizing fasciitis or severe soft tissue inflammations. Exome sequencing reveals truncating monoallelic loss-of-function variants of *nuclear factor κ light-chain enhancer of activated B cells* (*NFKB1*) in affected patients. In patients' macrophages and in *NFKB1*-variant-bearing THP-1 cells, activation increases both interleukin (IL)-1β secretion and IFN-I signaling. Truncation of NF-κB1 impairs autophagy, accompanied by the accumulation of reactive oxygen species and reduced degradation of inflammasome receptor nucleotide-binding oligomerization domain, leucine-rich repeat-containing protein 3 (NLRP3), and Toll/IL-1 receptor domain-containing adaptor protein inducing IFN-β (TRIF), thus leading to combined excessive inflammasome and IFN-I activity. Many of the patients respond to anti-inflammatory treatment, and targeting IL-1β and/or IFN-I signaling could represent a therapeutic approach for these patients.

INTRODUCTION

Inflammation has a vital role in defense against invading pathogens, and it also initiates tissue repair after trauma, but failure to properly control and limit the inflammatory reaction leads to detrimental tissue damage and potentially to life-threatening systemic inflammation and multiorgan failure. Genetic factors play an important role in the immune responses against danger

© 2024 The Authors. Published by Elsevier Inc.
This is an open access article under the CC BY license (http://creativecommons.org/licenses/by/4.0/).

Arzu Beklen,[1] Marcelina Bilicka,[1] Sampsa Matikainen,[1] Dan C. Nordström,[1,14] Meri Kaustio,[10] Ulla Wartiovaara-Kautto,[15,16] Outi Kilpivaara,[16,17,18,19] Christoph Klein,[20] Fabian Hauck,[20] Tiina Jahkola,[21] Timo Hautala,[22] Markku Varjosalo,[2] Goncalo Barreto,[1] Mikko R.J. Seppänen,[23,24,28,*] and Kari K. Eklund[1,25,26,28,29,*]

[21]Department of Plastic Surgery, HUH, 00029 Helsinki, Finland
[22]Research Unit of Internal Medicine and Biomedicine, OU, and Infectious Diseases Clinic, OUH, 90014 Oulu, Finland
[23]Adult Immunodeficiency Unit, Infectious Diseases, Inflammation Center, HUH and UH, 00029 Helsinki, Finland
[24]Rare Disease Center, Children and Adolescents, HUH and UH, 00029 Helsinki, Finland
[25]Department of Rheumatology, HUH and UH, 00029 Helsinki, Finland
[26]Orton Orthopaedic Hospital, 00280 Helsinki, Finland
[27]These authors contributed equally
[28]These authors contributed equally
[29]Lead contact
*Correspondence: mikko.seppanen@hus.fi (M.R.J.S.), kari.eklund@hus.fi (K.K.E.)
https://doi.org/10.1016/j.xcrm.2024.101503

signals. This is exemplified by autoinflammatory diseases, which are a group of rare diseases characterized by excessive activation of the innate immune system and recurrent attacks of local or systemic sterile inflammation. In most autoinflammatory diseases, the excessive immune activation is caused by monogenic germline or somatic mutations of genes regulating key immune signaling pathways, such as those regulating inflammasome activation, nuclear factor κ light-chain enhancer of activated B cells (NF-κB), or type I interferon (IFN-I) signaling pathways.[1–3]

The NF-κB pathway is a master regulator of inflammatory gene expression. It is activated by various microbial components via Toll-like receptors (TLRs) and several cytokines such as tumor necrosis factor (TNF). NF-κB transcription factor family members p65 (also known as RelA), RelB, c-Rel, and the cleaved forms of NF-κB1 (p50) and NF-κB2 (p52) form complexes via homo- and heteromeric dimerization.[4] The p65:p50 heterodimer is the canonical inducer of proinflammatory gene expression, whereas the p50:p50 homodimer mainly functions as a transcriptional repressor of NF-κB signaling.[5] NF-κB complexes regulate the proliferation of cells and the expression of a wide variety of proinflammatory cytokines and chemokines[6]; NF-κB also induces the expression of nucleotide-binding oligomerization domain, leucine-rich repeat-containing protein 3 (NLRP3) inflammasome proteins. In addition to inducing proinflammatory signaling, NF-κB exerts negative regulation, for example by inducing the expression of several NF-κB inhibitors.[7]

In many autoinflammatory diseases, the pathogenic genetic variant causes either direct or indirect activation of the NLRP3 inflammasome. Activation of the NLRP3 inflammasome leads to proteolytic processing and secretion of interleukin (IL)-1β and IL-18.[8] Classical NLRP3 inflammasome activation involves two steps. In the priming step, provided by the activation of TLRs or cytokine receptors, NF-κB-mediated expression of inflammasome components and pro-IL-1β is induced.[9] A subsequent second stimulus induces a disruption of cellular homeostasis, such as changes of intracellular ion concentrations, lysosomal rupture, and release of mitochondrial reactive oxygen species (mtROS) and mtDNA, which then launches the assembly and activation of the NLRP3 inflammasome complex.[8] Activation of the NLRP3 inflammasome is regulated at several levels. In addition to activating signals, NF-κB provides also negative feedback regulation of inflammasome activity by inducing expression of an autophagy receptor protein *SQSTM1*.[10] Macroautophagy (from here on referred to as autophagy) represents an important negative regulator of NLRP3 inflammasome activity. Basal autophagy eliminates both the activators of the NLRP3 inflammasome, such as cytoplasmic mtROS and mtDNA,[11] and the inflammasome components to shut down inflammasome activation.[12,13]

Necrotizing fasciitis (NF) is a severe infectious disease that imposes catastrophic consequences to the patients, as the treatment often necessitates major surgical tissue revisions and limb amputations.[14] Sometimes no pathogen(s) can be identified, or the severity of the inflammation is disproportionate with respect to the bacterial load, suggesting that host factors play a significant role in the course of the disease. We have previously described a single family in which two brothers carrying a heterozygous germline truncating mutation in *NFKB1* developed severe NF after a minor trauma or surgery in the absence of significant bacterial growth.[15] Thereafter, several case reports have described cutaneous inflammatory reactions including pyoderma gangrenosum (PG),[16–18] sterile inflammation and delayed wound healing,[19] or recurrent necrotizing cellulitis[20] in patients with truncating mutations of *NFKB1*. Since our previous report,[15] we have encountered five additional unrelated families from four different countries in which patients with monoallelic truncating variants of *NFKB1* presented with severe NF or other severe soft tissue inflammation. We have now elucidated the underlying molecular mechanisms by which impaired proinflammatory NF-κB1 signaling leads to intense hyperinflammatory reactions. We describe a mechanism of autoinflammation in which loss of NF-κB1 signaling leads to impaired autophagy and thereby to IFN-I and NLRP3 inflammasome-mediated autoinflammation. Finally, we suggest that some of the patients with NF or other necrotizing soft tissue inflammation could benefit from use of anti-inflammatory therapy.

RESULTS

Patient description

Two brothers (F1.II-1, F1.II-5) of family I presented at adulthood with severe NF after minor trauma or surgery.[15,21,22] F1.III-8, son of F1.II-5, is an asymptomatic mutation carrier (36 years at the time of publication).[21,22] The index patient of family II (F2.II-1) developed severe NF after routine knee replacement surgery at the age of 63.[21] The index patient of family III (F3.II-1) presented with recurrent epiglottitis, scrotal ulcers, and common variable immunodeficiency.[21] The index patient of family IV (F4.II-2), a

CellPress
OPEN ACCESS

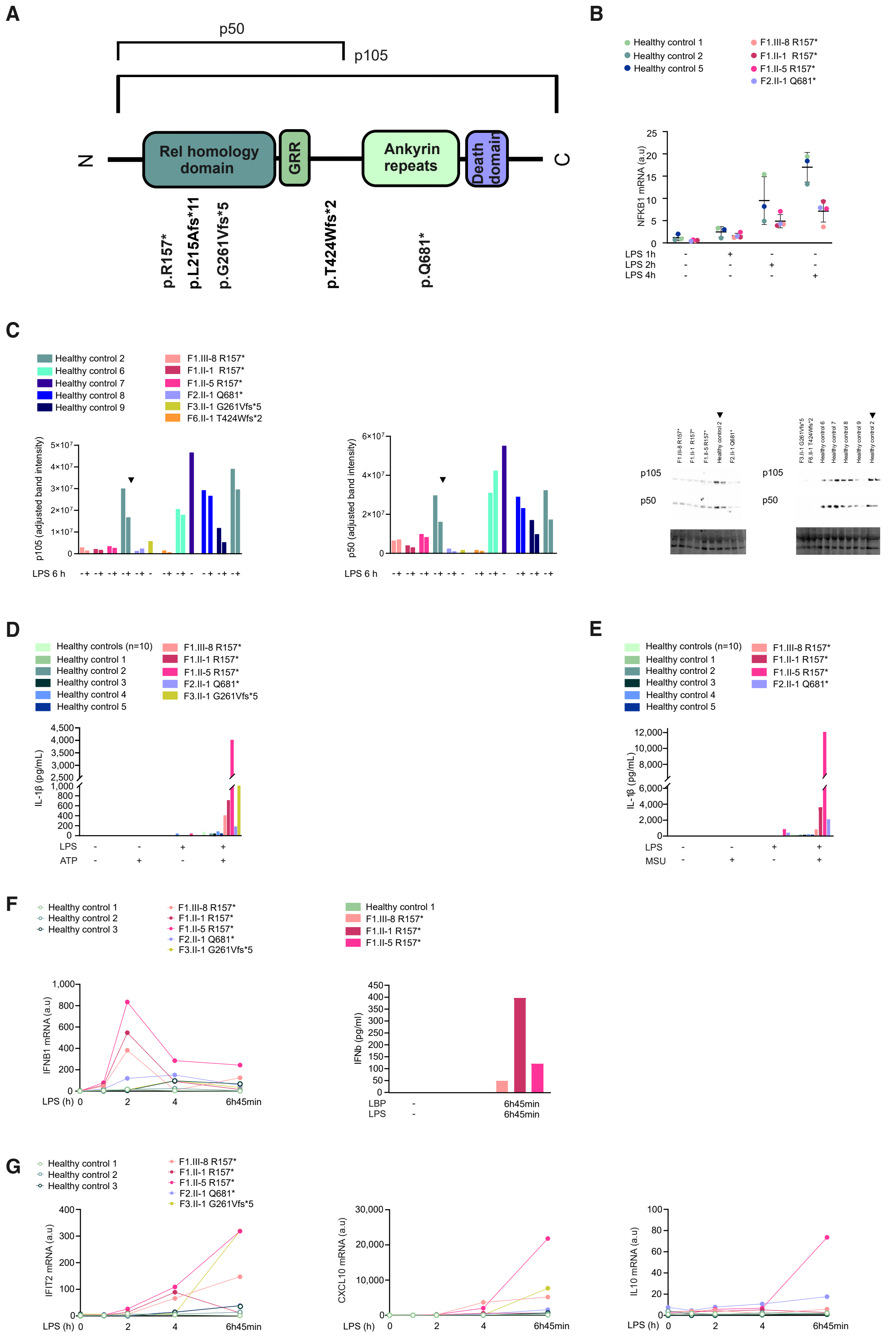

(legend on next page)

newborn baby, developed severe omphalitis and PG after vacuum-assisted vaginal delivery. The index patient of family V (F5.II-3) developed deep necrotizing ocular cellulitis at the age of 2 and later, at the age of 14, PG. The index patient of family VI (F6.II-1) presented with intermittent fever since the age of 28 and developed cellulitis and myofascitis at the age of 30. A full description of the clinical manifestations can be found in the STAR Methods. The ancestry, patients' main clinical manifestations, and *NFKB1* variant information are shown in Tables S1 and S2 and the pedigrees in Figure S1.

Patient macrophages bearing truncating variants of *NFKB1* display pronounced activation of the NLRP3 inflammasome and IFN-I signaling

The heterozygous *NFKB1* variants identified in affected patients introduced an early (p.R157*, p.L215Afs*11, p.G261Vfs*5), intermediate (p.T424Wfs*2), or late (p.Q681*) premature stop codon in the *NFKB1* transcript (Figure 1A). Truncations of *NFKB1* reduced *NFKB1* transcript levels by roughly half in human monocyte-derived macrophages (HMDMs) (Figure 1B). Along with that, protein expression of p105 and p50 was substantially reduced both in unstimulated and lipopolysaccharide (LPS)-stimulated patient-derived peripheral blood mononuclear cells (PBMCs) compared to healthy controls (Figures 1C and S2A). Patient macrophages demonstrated significantly increased secretion of IL-1β upon inflammasome activation with ATP and monosodium urate crystals (Figures 1D and 1E). Additionally, enhanced expression of IFN-β at the transcript and protein levels was observed in LPS-treated patient macrophages (Figure 1F). Macrophages of some patients also showed increased secretion of TNF (Figure S2B). Expression of the IFN-stimulated genes (ISGs) *IFIT2* and *CXCL10* was enhanced in all patients, and the expression of *IL10* was enhanced in all patients except F3.II-1 (Figure 1G). The variants did not alter the expression of all tested ISGs, as expression of guanylate-binding protein 1 (*GBP1*) and *GBP5* displayed no consistent changes (Figure S2C). Blocking IFN-I signaling by anti-IFN-α/β receptor (IFNAR) antibody only modestly reduced IL-1β secretion (Figure S2D), suggesting that IFN-I signaling did not contribute significantly to NLRP3 inflammasome activation.

Truncating mutation and knockout of *NFKB1* lead to activation of NLRP3 inflammasome and IFN-I signaling

To elucidate in more detail the impact of *NFKB1* truncation on cellular signaling, we generated THP-1 monocyte cell lines carrying the shortest truncating variant (p.R157*, $NFKB1^{R157*/R157*}$) and homozygotic ($NFKB1^{-/-}$) and heterozygotic ($NFKB1^{-/+}$) *NFKB1* knockouts (KOs) using CRISPR-Cas9 gene editing. No *NFKB1* gene expression was detected in $NFKB1^{R157*/R157*}$ THP-1 monocytes (Figure S2E), nor was there expression of p105 or p50 proteins in $NFKB1^{R157*/R157*}$ or $NFKB1^{-/-}$ THP-1 monocytes (Figure 2A), consistent with nonsense-mediated RNA decay. In $NFKB1^{-/+}$ THP-1 monocytes, the expression of p105/p50 was about half of that of the wild type (WT) (Figures 2A and S2F). Similar to the patient's cells, secretion of IL-1β (Figure 2B), and expression of *IFNB1* and ISGs, apart from *GBP5*, were highly upregulated in activated $NFKB1^{R157*/R157*}$ THP-1 monocytes (Figures 2C and S2G). Anti-IFNAR antibody completely blocked LPS-induced *IFIT2* expression both in $NFKB1^{R157*/R157*}$ and in WT THP-1 monocytes (Figure S2H), confirming its dependency on autocrine or paracrine secretion of IFN-I.

To study whether enhanced IL-1β secretion in $NFKB1^{R157*/R157*}$ THP-1 monocytes was inflammasome dependent, we assessed the activation of caspase-1. Inflammasome activation culminates in autocatalytic activation of pro-caspase-1 and formation of the active caspase-1 complex, which subsequently processes pro-IL-1β to its mature secreted form.[23] As expected, a caspase-1/4 inhibitor, Z-YVAD-FMK, abrogated the secretion of IL-1β (Figure 2D). In addition to cleavage of pro-IL-1β, active caspase-1 also cleaves gasdermin D (GSDMD), thereby causing pyroptotic cell death.[8] In concordance with enhanced IL-1β secretion, secretion of the pyroptosis marker lactate dehydrogenase (LDH) was also increased in $NFKB1^{R157*/R157*}$ THP-1 monocytes compared to WT, and this was modestly inhibited by the caspase-1/4 inhibitor (Figure S2I). To verify the dependency of IL-1β secretion on the NLRP3 inflammasome, we studied the interaction of NLRP3 with apoptosis-associated spec-like protein containing a caspase activation and recruitment domain (ASC) by co-immunoprecipitation assay and the inhibition of IL-1β secretion using a specific inhibitor of NLRP3, MCC950. While MCC950 inhibited secretion of IL-1β to a similar extent in $NFKB1^{R157*/R157*}$ and WT THP-1 monocytes (Figure 2E), the interaction of ASC with NLRP3 was more pronounced in $NFKB1^{R157*/R157*}$ THP-1 monocytes compared to WT (Figure 2F). Enhanced secretion of IL-1β was observed also in $NFKB1^{-/-}$ THP-1 monocytes (Figure 2G), suggesting that the phenotype of $NFKB1^{R157*/R157*}$ cells is comparable to the full KO of *NFKB1*. Secretion of IL-1β in $NFKB1^{-/+}$

Figure 1. Truncating mutations of *NFKB1* cause enhanced NLRP3 inflammasome activation and aberrant gene expression in patient-derived macrophages

(A) Schematic representation of p105/p50 protein domains and sites of mutations. Conserved Rel homology domain includes sequences needed for nuclear location, binding to DNA, and dimer formation with Rel family proteins and inhibitor proteins. GRR, glycine-rich region.

(B and C) HMDMs (B) or PBMCs (C) were stimulated with LPS for indicated times and *NFKB1* (B) mRNA or (C) protein expression of p105 and p50 was analyzed by RT-qPCR or western blot. Quantifications, blots, and total protein loading (TPL) are shown. The data are shown as mean ± SD. Healthy control 2 (indicated with the arrowhead) is included twice to enable comparison between the blots.

(D and E) HMDMs were stimulated with (D) LPS and ATP or (E) monosodium urate crystals (MSUs) and secretion of mature IL-1β was detected by ELISA. Average of 10 separately processed healthy volunteers is also shown.

(F and G) HMDMs were activated with LPS or LPS and LPS-binding protein (LBP) for indicated times. Gene expression and secretion of IFN-β were analyzed by RT-qPCR (left) and ELISA (right) (F) and expressions of *IFIT2*, *CXCL10*, and *IL10* were analyzed by RT-qPCR (G).

(B) 3 controls, 4 variant carriers; (C) 5 controls, 6 variant carriers; (D) 15 controls, 5 variant carriers; (E) 15 controls, 4 variant carriers; (F) RT-qPCR: 3 controls, 5 variant carriers, ELISA: 1 control, 3 variant carriers; (G) 3 controls, 5 variant carriers.

See also Figures S1 and S2.

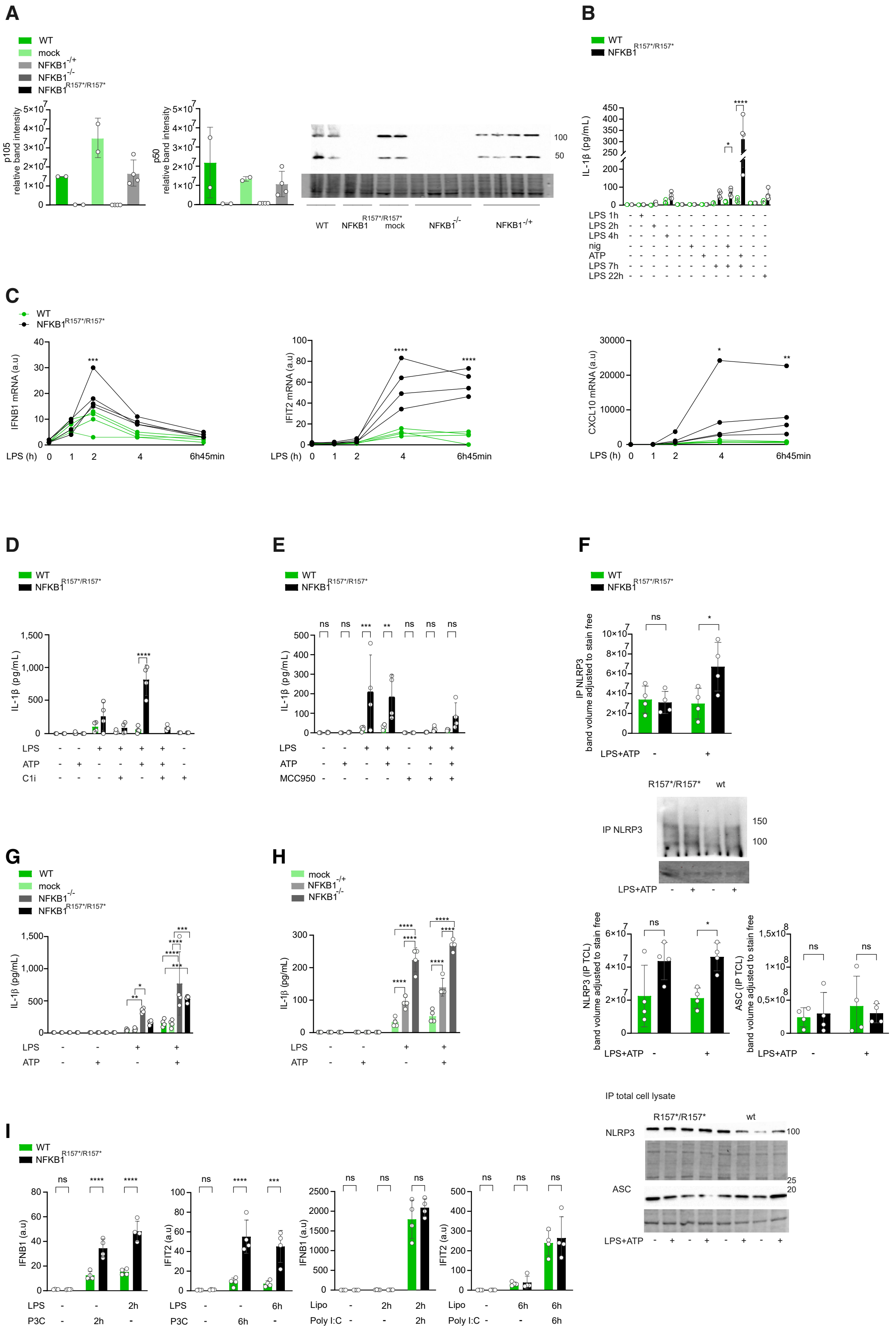

(legend on next page)

THP-1 monocytes was intermediate between cells transfected with mock guide and full KO (*NFKB1*$^{-/-}$) THP-1 monocytes (Figure 2H), suggesting that the secretion of IL-1β is inversely proportional to the level of *NFKB1*. In response to LPS activation, both *NFKB1*$^{R157*/R157*}$ and *NFKB1*$^{-/-}$ THP-1 monocytes secreted significantly more TNF compared to WT, with the secretion being even more pronounced in *NFKB1*$^{R157*/R157*}$ compared to *NFKB1*$^{-/-}$ (Figure S2J).

TLR engagement signals downstream via two major adaptors, TIRAP-MyD88 and TRAM-TRIF.[24] We used Pam_3Cys-SKKKK, a TLR1:2 ligand that signals via TIRAP-MyD88 and passes Toll/IL-1 receptor domain-containing adaptor protein inducing IFN-β (TRIF) activation, to study whether enhanced NLRP3 inflammasome activation in cells carrying truncating variants of *NFKB1* was dependent on TRAM/TRIF signaling. Stimulation of patient macrophages with Pam_3Cys-SKKKK induced more vigorous IL-1β release after adding ATP to stimulate the NLRP3 inflammasome in patients F1.II-5, F2.II-1, and F3.II-1, but not in F1.II-1 or F1.III-8, compared to healthy controls (Figure S3A). In THP-1 monocytes, Pam_3Cys-SKKKK consistently induced stronger IL-1β secretion after ATP stimulation compared to LPS, and again, we observed more vigorous IL-1β secretion in *NFKB1*$^{R157*/R157*}$ compared to WT, suggesting that the enhanced NLRP3 activation was not restricted to TLR4-mediated priming (Figure S3A). Of the other inflammasome activators, we studied the activation of the AIM2 inflammasome and noncanonical caspase-4/5 inflammasome. Transfection of poly (dA:dT), but not of LPS, resulted in increased IL-1β secretion in *NFKB1*$^{R157*/R157*}$ THP-1 monocytes compared to WT (Figures S3B and S3C). Similar to THP-1 monocytes, no consistent differences in secretion of IL-1β in response to the noncanonical inflammasome activation were observed between HMDMs derived from patients and healthy controls (Figure S3D). These results indicate that the p.R157* variant enhances the assembly and activation of the NLRP3 inflammasome after priming via TLR2 and TLR4 as well as activation of the AIM2 inflammasome but not that of the noncanonical inflammasome.

To further study the enhanced ISG response, we activated different pattern recognition receptors (PRRs), TLR2, TLR4, and RIG-I/MDA-5, all upstream of IFN-I induction, and analyzed expression of *IFNB1* and *IFIT2*.[25] Both LPS and Pam_3Cys-SKKKK resulted in a significantly higher IFN-I response in *NFKB1*$^{R157*/R157*}$ THP-1 monocytes compared to WT, but there was no difference in expression between the *NFKB1*$^{R157*/R157*}$ and WT in monocytes when transfecting with low-molecular-weight poly(I:C), an activator of RIG-I/MDA-5 (Figure 2I). Our results suggest that enhanced IFN-I response follows activation of TLR2 or TLR4 but not RIG-I/MDA5.

Truncations of NF-κB1 disrupt the protein-protein interactions of NF-κB1 with its target proteins

Through an unbiased approach, we studied the interactomes of NF-κB1 variants and WT protein by analyzing the stable and proximate interactions of the tagged constructs by quantitative affinity purification mass spectrometry and BioID (Figure 3A).[26] Only WT NF-κB1 and the late truncating construct p.Q681* yielded stable interactomes with NF-κB family proteins. In the shorter truncating variants of NF-κB1 (p.R157*, p.G261Vfs*5), the stop codon is located within the Rel homology domain (RHD), likely rendering them unable to form interactions with NF-κB proteins and to produce p105 protein, which is responsible for interactions with non-NF-κB family proteins.[27] As expected, the interactions of p.R157* and p.G261Vfs*5 variants were lost to the point of preventing meaningful analysis (Figure 3B). Interactions of the p.Q681* variant were largely comparable to WT: the p.Q681* variant formed less interactions with p50, marginally more interactions with p65, and slightly reduced interactions with M3K8. Interactions with TNIP1, HACL2, and FLOT1 were lost, but interactions with several NF-κB family proteins were slightly increased. In addition, an interaction with M1IP1 was detected for the p.Q681* variant (Figures 3B; Table S3). Figure 3C presents the interactome-based estimated localizations of WT and p.Q681* NF-κB1 proteins. The highest localization signal for WT NF-κB1 was in intermediate filaments but was also noted in the nucleoplasm. p.Q681* showed reduced localization in filaments, and the highest scores were detected in the nucleoplasm. In addition, there was a localization to the Golgi, likely reflecting the sorting of misfolded proteins in the Golgi apparatus to other organelles for degradation or secretion.[28]

To gain an overview of the impact of the p.R157* variant on cellular functions, we performed global proteomics analysis on

Figure 2. NLRP3 inflammasome activation is proportional to expression of *NFKB1*

(A) NF-κB1 basal expression in *NFKB1*$^{R157*/R157*}$, *NFKB1*$^{-/-}$, or *NFKB1*$^{-/+}$ and WT or mock THP-1 monocyte lysates. Blot and TPL are shown.

(B) *NFKB1*$^{R157*/R157*}$ and WT THP-1 monocytes were stimulated with LPS and ATP or nigericin. Secreted mature IL-1β was detected by ELISA.

(C) *NFKB1*$^{R157*/R157*}$ and WT THP-1 monocytes were activated with LPS for indicated times. Expression of *IFNB1*, *IFIT2*, and *CXCL10* was analyzed by RT-qPCR.

(D) *NFKB1*$^{R157*/R157*}$ and WT THP-1 monocytes were stimulated with LPS and treated with caspase-1/4 inhibitor (Z-YVAD-FMK) 1 h before ATP. Secreted mature IL-1β was detected by ELISA.

(E) *NFKB1*$^{R157*/R157*}$ and WT THP-1 monocytes were stimulated with LPS and treated with specific NLRP3 inhibitor (MCC950) 1 h before ATP. Secreted mature IL-1β was detected by ELISA.

(F) Immunoblot analysis of NLRP3 immunoprecipitated with ASC and immunoblots of ASC and NLRP3 from lysates of *NFKB1*$^{R157*/R157*}$ and WT THP-1 monocytes primed with LPS and activated with ATP or left untreated. Quantifications, representative blots, and TPL are shown.

(G and H) *NFKB1*$^{R157*/R157*}$, *NFKB1*$^{-/-}$, mock, and WT THP-1 monocytes (G) or *NFKB1*$^{-/-}$, *NFKB1*$^{-/+}$, and mock THP-1 monocytes (H) were activated with LPS and ATP. Secreted mature IL-1β was detected by ELISA.

(I) *NFKB1*$^{R157*/R157*}$ and WT THP-1 monocytes were stimulated with LPS or Pam3Cys-SKKKK or transfected with low-molecular-weight poly(I:C) to activate RIG-I/MDA-5. Expressions of *IFNB1* and *IFIT2* were analyzed by RT-qPCR.

Two-way ANOVA (B–E and G–I) or two-way repeated measures (RM) ANOVA (F) followed by Tukey's (G and H) or Šidak's (B–F and I) tests. The data are shown as mean ± SD. *$p < 0.05$, **$p < 0.01$, ***$p < 0.001$, ****$p < 0.0001$. (A) WT, *NFKB1*$^{-/-}$, and mock $n = 2$, *NFKB1*$^{-/-}$ and *NFKB1*$^{-/+}$ $n = 4$; (B–I) $n = 4$.

See also Figures S2 and S3.

CellPress
OPEN ACCESS

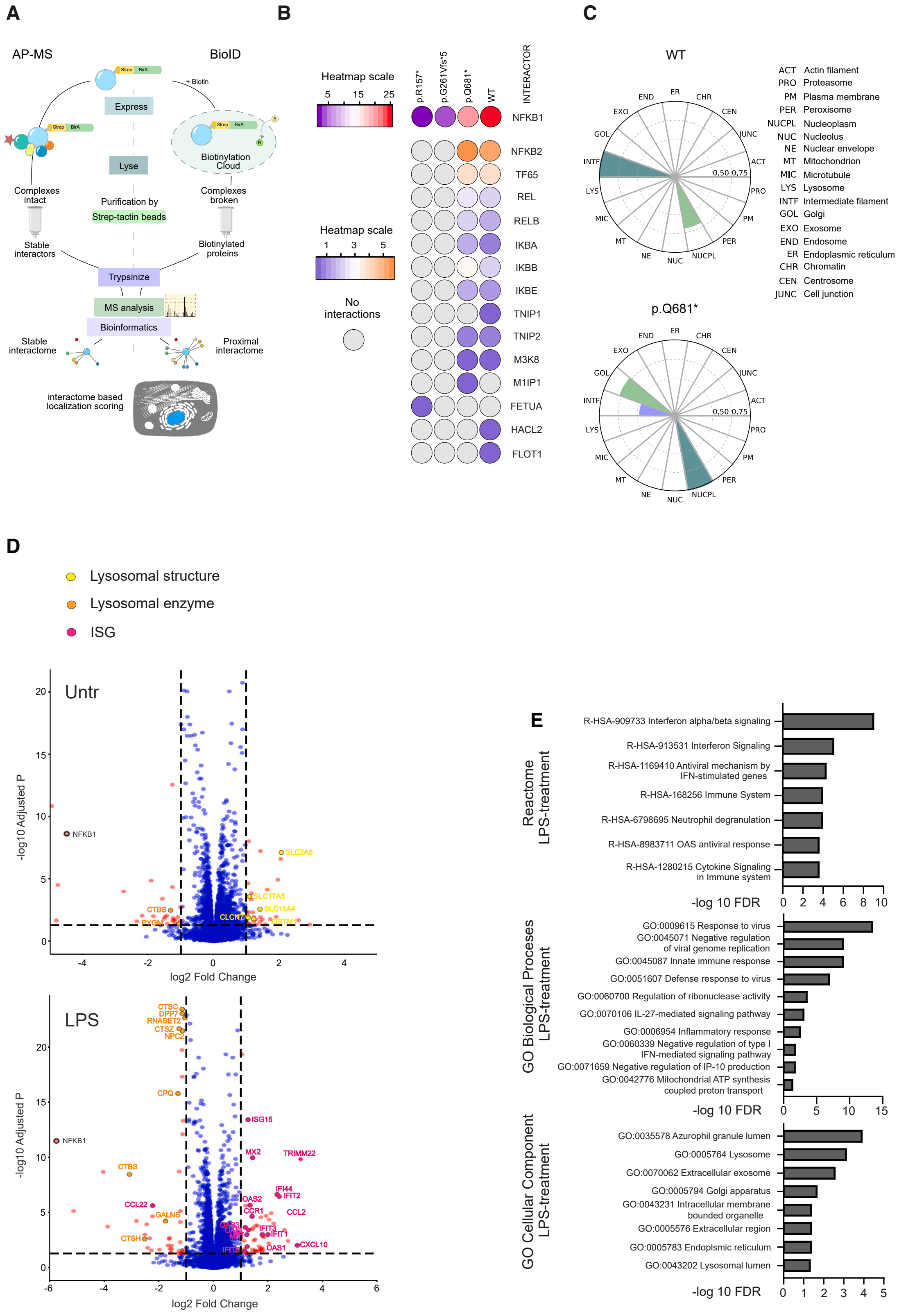

(legend on next page)

THP-1 monocytes carrying the p.R157* variant and WT. In untreated cells, we detected 79 significantly differentially expressed proteins ([log fold] > 1 and $p < 0.05$), of which 35 were downregulated and 44 upregulated (Figure 3D; Data S1). Our results demonstrate differential regulation of Gene Ontology (GO) Biological Process (BP) cellular oxidant detoxification, referring to a group of ROS-responding genes upregulated in p.R157*-variant-carrying cells (Data S2). Analysis of GO cellular components (CCs) revealed significant enrichment of exosome signaling (Data S2). LPS stimulation significantly downregulated the expression of 46 proteins and upregulated the expression of 54 proteins (Figure 3D; Data S1) in *NFKB1*$^{R157*/R157*}$ THP-1 monocytes compared to WT. Among the most significantly increased proteins ([log fold] > 2), 8 of 11 proteins were encoded by *IFNB*-regulated genes (Interferome: https://www.interferome.org). p.R157*-variant-carrying cells were enriched for GO-BP and Reactome terms that co-occur with IFN-β response, and GO CCs were enriched for terms related to lysosome and secretory vesicle trafficking (Figure 3E; Data S2), indicating that the p.R157* variant causes significant differences in IFN-I signaling and, interestingly, downregulates the expression of several lysosomal enzymes. Of the total of 100 differentially regulated proteins, 39 were putative regulators of autophagic flux in LPS-stimulated THP-1 monocytes (Data S1).

Truncations of NF-κB1 have no significant impact on the expression of NLRP3 inflammasome-related genes

To study the impact of *NFKB1* variants on transcriptional regulation of NF-κB, inflammasome, and IFN signaling pathways, we assessed the baseline expression levels in patients' PBMCs using a direct mRNA analysis. No consistent differences were observed in genes related to NF-κB, JAK/STAT, or inflammasome pathways (Figures 4A and 4B; Table S4). However, in individual patients, a marked increase in expression of those pathways as well as of ILs and ISGs was observed. Expression of inflammasome components is a limiting factor for the NLRP3 inflammasome activation.[9] Their expression is initiated by a so-called "priming signal" that activates NF-κB signaling, resulting in the expression of the inducible NLRP3 inflammasome components *NLRP3* and *IL1B*. Other inflammasome proteins, *ASC*, *CASP1*, and *IL18*, are expressed constitutively.[8] The kinetics of NLRP3 inflammasome component expression was determined in LPS-primed cells. Compared to WT, no significant changes were observed in the expression of *IL1B*, *NLRP3*, *ASC*, *CASP1*, or *IL18* in unstimulated or LPS-stimulated patient macrophages or *NFKB1*$^{R157*/R157*}$ THP-1 monocytes (Figures S3E and S3F), and neither did the variants of *NFKB1* have a consistent effect on the expression of selected NF-κB targets (*NFKBIA*, *IL6*, *TNF*) compared to healthy controls (Figure S3G) in patient-derived macrophages. These findings suggest that transcriptional upregulation does not play a major part in the excessive NLRP3 inflammasome activation observed in the patients' macrophages. Immunofluorescence analysis of patient macrophages showed diminished LPS-induced translocation of p65 and p50 to the nucleus in p.R157*-bearing cells, whereas cells bearing the p.Q681* variant more closely resembled the healthy controls, in line with the interactome analysis (Figure 4C). The p.Q681* truncation is outside of the p50 coding area, theoretically preserving its functions to some extent, which could explain the greater similarity to the healthy controls. In all mutation carriers, markedly more p52 was directed to the nucleus, suggesting that noncanonical NF-κB activation might compensate for the deficiency of p50 and prolong NF-κB signaling (Figure 4C).[6,29] We observed increased nuclear localization of p52 also in *NFKB1*$^{R157*/R157*}$ THP-1 monocytes, but the translocation of p65 to the nucleus showed no change (Figure S4A), consistent with the unaffected transcription of NF-κB-regulated genes.

Autophagic flux is reduced in cells with truncating mutations of *NFKB1*

The NLRP3 inflammasome is activated by disturbances of cellular homeostasis, including mitochondrial damage and subsequent release of mtROS and mtDNA.[30] We found increased levels of mtROS in untreated *NFKB1*$^{R157*/R157*}$ THP-1 monocytes compared to WT (Figure 5A). To study whether mtROS has a role in NLRP3 inflammasome activation, we used MitoTempo, a mitochondrial-specific antioxidant, to inhibit mtROS. MitoTempo reduced secretion of IL-1β in patient and healthy control macrophages and in *NFKB1*$^{R157*/R157*}$ and WT THP-1 monocytes (Figure 5B). To inhibit the release of both mtROS and mtDNA, we created ρ^0 cells by low-dose ethidium bromide treatment, which depletes mtDNA (Figure S4B).[31] In ρ^0 THP-1 monocytes, IL-1β secretion from both *NFKB1*$^{R157*/R157*}$ and WT was blocked (Figure 5C). These results suggest that in cells with truncating mutations of *NFKB1*, increased release of mtROS and mtDNA from damaged mitochondria leads to enhanced NLRP3 inflammasome activation.

Organelles, larger protein complexes, and aggregates are primarily removed by autophagy. Elimination of proinflammatory structures and compounds, including mtROS, is critical to

Figure 3. Truncation of NF-κB1 alters protein interaction and expression profiles

The interactomes of mutant and WT NF-κB1 were characterized by analyzing their N-terminal MAC tag constructs with affinity purification mass spectrometry (AP-MS) and BioID.

(A) Workflow of AP-MS and BioID protein-protein interaction analysis to identify both stable and proximal protein-protein interactomes.

(B) AP-MS identified stable interactome of WT NF-κB1 and NF-κB1 variant constructs with interaction values normalized to bait abundance. Interactions of NF-κB1 variants with NF-κB1 are displayed on a different scale than the rest of the interactors.

(C) Cellular localization of WT NF-κB1 and p.Q681* variant based on their BioID interactomes.

(D) Volcano plots of all identified proteins from untreated and LPS-stimulated (6 h 45 min) *NFKB1*$^{R157*/R157*}$ and WT THP-1 monocytes. Proteins are plotted according to their fold change and adjusted p value.

(E) Reactome pathway and GO enrichment analysis of significantly differentially expressed proteins ([log fold] > 1 and $p < 0.05$) in LPS-stimulated *NFKB1*$^{R157*/R157*}$ and WT THP-1 monocytes. Proteins are plotted according to their $-\log_{10}$ false discovery rate (FDR) values.

(B and C) $n = 2$, (D) $n = 10$.

See also Table S3 and Data S1 and S2.

CellPress
OPEN ACCESS

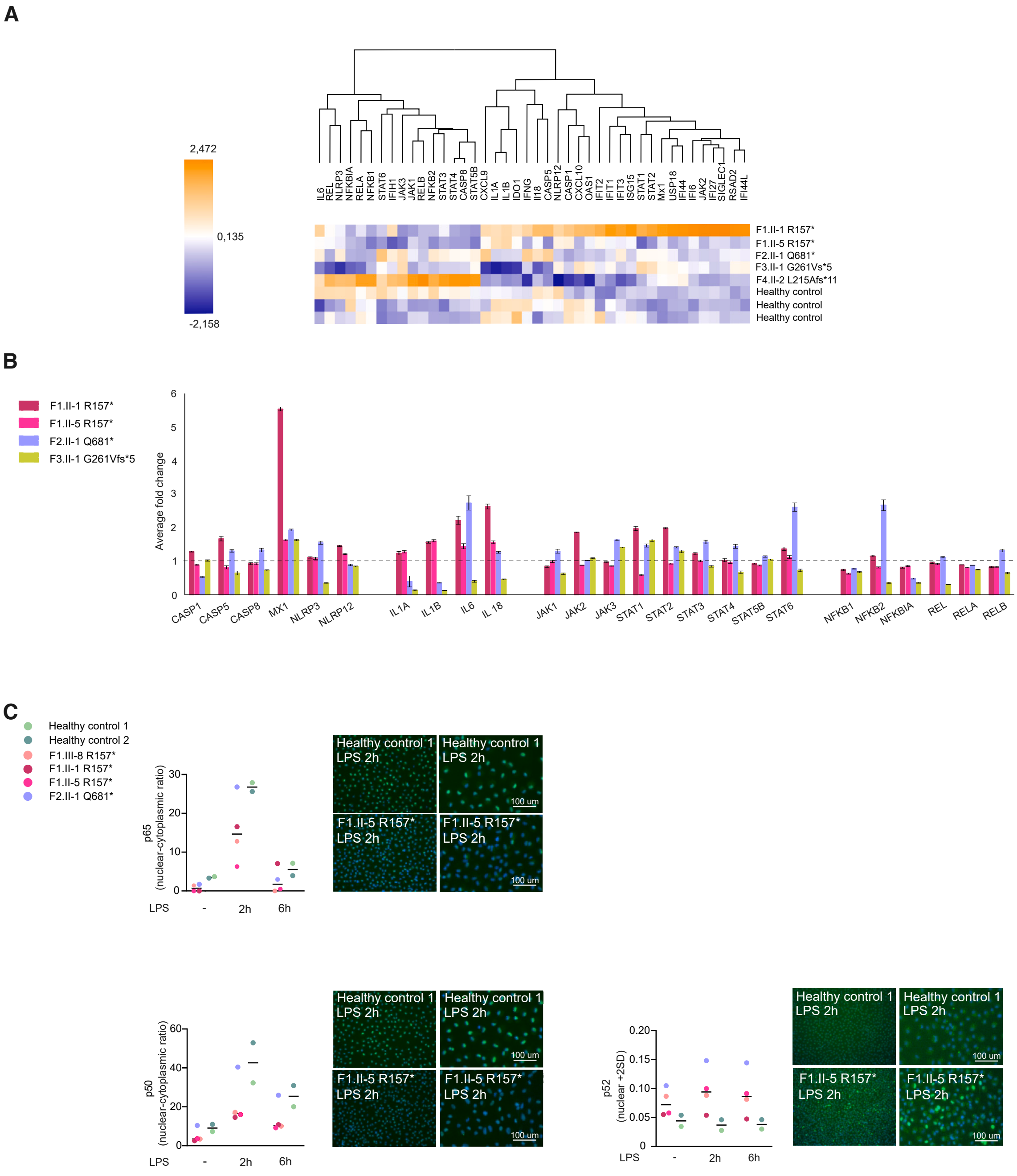

Figure 4. Aberrant signaling in *NFKB1*-variant-bearing cells

(A) A heatmap showing direct digital analysis of mRNA (NanoString) of untreated PBMCs derived from *NFKB1* variant carriers and healthy controls.

(B) Fold changes of adult *NFKB1* variant carriers' inflammasome, interleukin, JAK/STAT, and NF-κB pathway-linked genes.

(C) HMDMs were activated with LPS for indicated times and stained for p65, p50, and p52; representative stainings and quantifications of stainings (median) are shown. Scale bar 100 μm.

(A) 3 controls, 5 variant carriers (3 technical replicates); (B) 3 controls, 4 variant carriers; (C) 2 controls, 3 p.R157* variant carriers, 1 p.Q681* variant carrier. See also Figures S3 and S4 and Table S4.

maintain cellular homeostasis.[32] NF-κB activation triggers expression of autophagy receptor sequestosome 1 (SQSTM1), which is a negative feedback mechanism that targets NLRP3 inflammasome complexes and damaged mitochondria for autophagic degradation.[10] In patient cells, *NFKB1* variants had no explicit effect on LPS-induced *SQSTM1* mRNA expression, but a moderate reduction of *SQSTM1* expression was repeatedly observed in *NFKB1*$^{R157*/R157*}$ THP-1 monocytes (Figure S4C).

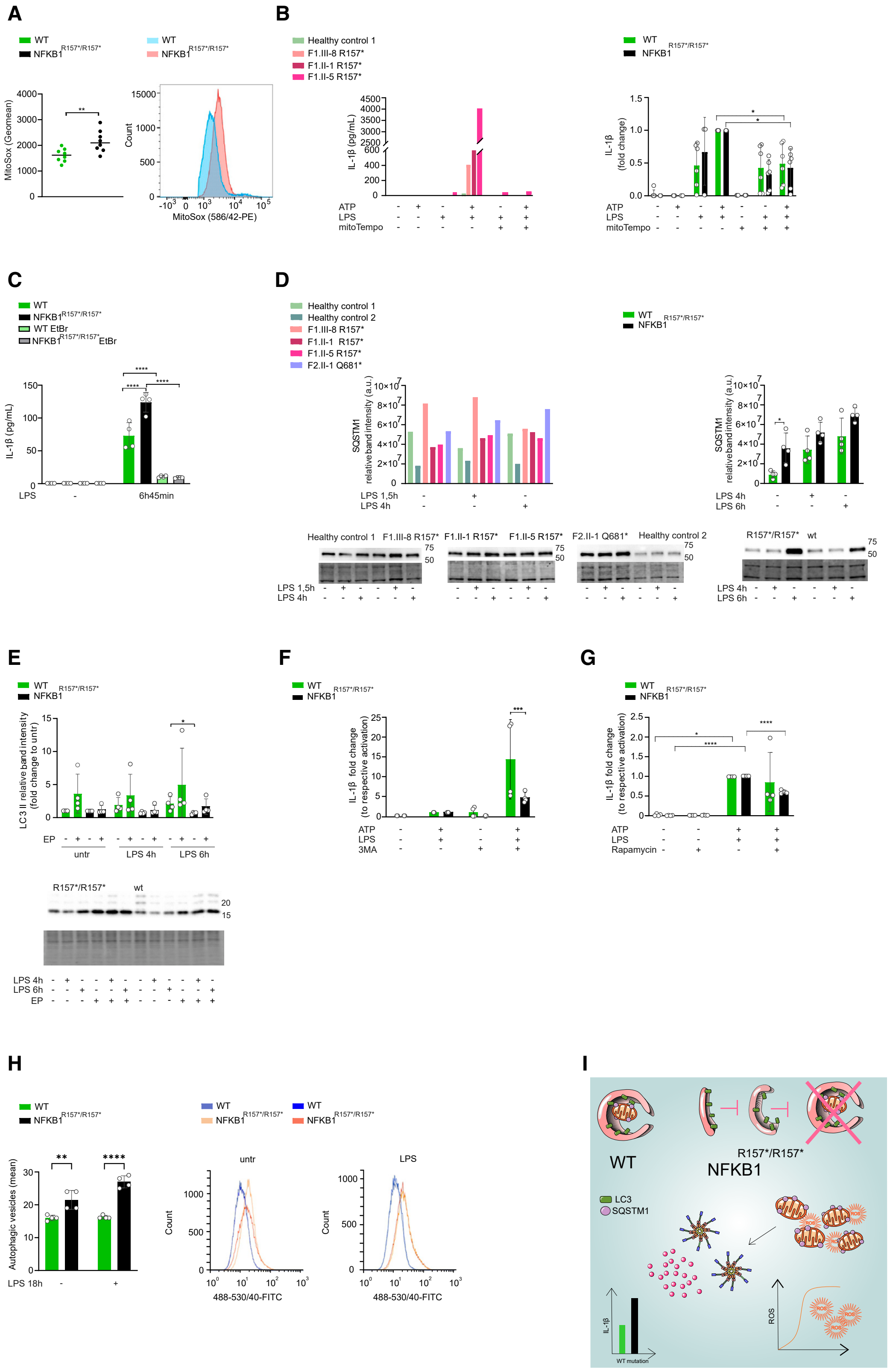

(legend on next page)

Despite reduced mRNA expression, SQSTM1 protein tended to accumulate in patient macrophages, and in *NFKB1*$^{R157*/R157*}$ THP-1 monocytes, its accumulation was statistically significant (Figure 5D), implying that deficient *NFKB1* expression reduces autophagic degradation. Autophagosome formation is recognized by the lipidation of microtubule-associated protein light chain 3-I (LC3-I) to LC3-II that participates in the formation of the phagophore membrane.[33] Selective autophagy receptors, including SQSTM1, interact with LC3-II and guide the cargo into the forming autophagosomes. Subsequently, SQSTM1 is degraded together with the cargo proteins by lysosomal proteases when autophagosomes fuse with lysosomes to form autolysosomes.[33] This flow of events is termed autophagic flux. Relatively less LC3-II was formed in *NFKB1*$^{R157*/R157*}$ THP-1 monocytes compared to WT (Figure 5E), suggesting that accumulation of SQSTM1 was caused by impaired autophagosome formation and/or reduced autolysosomal degradation. Also, when autophagic degradation was inhibited by protease inhibitors E-64d and pepstatin, a nonsignificant trend of higher LC3-II accumulation was observed in WT compared to *NFKB1*$^{R157*/R157*}$ THP-1 monocytes (Figure 5E), suggesting its higher degradation. Manipulation of autophagic flux reduced the relative differences between WT and the p.R157* variant. NLRP3 inflammasome activation in the presence of autophagy inhibitor 3-MA resulted in higher fold induction of IL-1β secretion in WT THP-1 monocytes compared to *NFKB1*$^{R157*/R157*}$ (Figure 5F). By contrast, the autophagy activator rapamycin significantly reduced the fold induction of IL-1β secretion in *NFKB1*$^{R157*/R157*}$ THP-1 monocytes, whereas only a minor inhibitory effect was observed in WT (Figure 5G). To study the rate of vesicle degradation, we quantitated the levels of fluorescently labeled autophagic vesicles. Despite their reduced formation, significantly higher levels of autophagic vesicles were detected in *NFKB1*$^{R157*/R157*}$ THP-1 monocytes compared to WT both in untreated cells and in the presence of LPS (Figures 5H and S4D), indicating that the autophagosomes accumulated, consistent with the accumulation of SQSTM1. These results suggest that truncation of NF-κB1 impairs autophagic degradation by reducing formation and degradation of autophagosomes (Figure 5I).

Impairment of autophagy underlies the activation of the NLRP3 inflammasome and type I interferonopathy in *NFKB1*-deficient cells

LPS induces expression of NLRP3 and TRIF but concomitantly increases autophagic flux to shut down inflammasome and IFN signaling.[12,13,34–36] In patient macrophages and in *NFKB1*$^{R157*/R157*}$ THP-1 monocytes, stimulation with LPS resulted in significantly higher intracellular levels of NLRP3 as compared to controls (Figure 6A). Likewise, a clear accumulation of pro-IL-1β as compared to control was observed in *NFKB1*$^{R157*/R157*}$ THP-1 monocytes, but this was ambiguous in patients' macrophages (Figure 6B). LPS stimulation decreased the level of intracellular TRIF in healthy controls and in WT THP-1 monocytes, whereas patients' macrophages and *NFKB1*$^{R157*/R157*}$ THP-1 monocytes showed a significant increase of TRIF levels (Figure 6C). Two patients (F1.II-1, FI.II-5) exhibited substantial accumulation of TRIF also without LPS stimulation. The p.R157* variant had no effect on the LPS-induced total protein secretion (Figure 6D), but the secretion of SQSTM1 was significantly reduced in *NFKB1*$^{R157*/R157*}$ THP-1 monocytes (Figure 6E). Autophagy cargo receptors are secreted via secretory autophagy; thus, the dramatically reduced levels of SQSTM1 in the cell supernatants indicate malfunction of autophagy.[37] Secretory autophagy is particularly important in the export of aggregation-prone proteins and organelles, such as mitochondria.[37,38] Although secretory autophagy is involved also in secretion of mature IL-1β, GSDMD pores and pyroptosis are the main pathways for its bulk secretion.[39] Our findings suggest that truncating mutations of *NFKB1* reduce autophagic flux, which leads to accumulation of TRIF and NLRP3 and, subsequently, to increased NLRP3 inflammasome activation and IFN-I signaling.[9,34,40]

DISCUSSION

Macrophages of patients with monoallelic truncating loss-of-function (LOF) variants of *NFKB1* and THP-1 monocytes carrying the *NFKB1* p.R157* variant exhibited a combination of significantly increased secretion of IL-1β and enhanced IFN-I signaling. In many of the patients, the hyperinflammatory

Figure 5. Truncating variants of *NFKB1* impair autophagy

(A) MitoSOX staining of untreated *NFKB1*$^{R157*/R157*}$ and WT THP-1 monocytes was analyzed by flow cytometry. Quantifications and representative plots are shown.

(B) HMDMs (left) or *NFKB1*$^{R157*/R157*}$ and WT THP-1 monocytes (right) were activated with LPS, and MitoTempo was applied 1 h before ATP. Secreted mature IL-1β was detected by ELISA.

(C) ρ^0 or conventional *NFKB1*$^{R157*/R157*}$ and WT THP-1 monocytes were activated with LPS and secreted mature IL-1β was detected by ELISA.

(D) HMDMs (left) or *NFKB1*$^{R157*/R157*}$ and WT THP-1 monocytes (right) were activated with LPS for indicated times and SQSTM1 was blotted from lysates. Quantifications, representative blots, and TPL are shown.

(E) *NFKB1*$^{R157*/R157*}$ and WT THP-1 monocytes were inhibited with E-64d and pepstatin A (abbreviated as EP) 1 h prior to LPS (for indicated times). LC3 was blotted from cell lysates. Quantifications, representative blot, and TPL are shown.

(F and G) *NFKB1*$^{R157*/R157*}$ and WT THP-1 monocytes were treated with LPS and (F) 3-MA (6 h 45 min) or (G) rapamycin (17 h 45 min) and ATP (for the last 45 min). Secreted mature IL-1β was detected by ELISA and is expressed as fold change relative to the respective cells stimulated with LPS and ATP.

(H) *NFKB1*$^{R157*/R157*}$ and WT THP-1 monocytes were activated with LPS (18 h). Cells staining positive for Hoechst (nuclear stain) and autophagic vesicle marker were analyzed by flow cytometry. Quantitation and representative plots are shown.

(I) Schematic representation of the effects of truncating *NFKB1* mutations on macroautophagy and NLRP3 inflammasome activation.

Wilcoxon test (A), one-way RM-ANOVA (B), or two-way ANOVA (C–H) followed by Dunnett's (B), Šidak's (D–G), or Tukey's test (C and H). The data are shown as mean ± SD. *$p < 0.05$, **$p < 0.01$, ***$p < 0.001$, ****$p < 0.0001$. (A) $n = 8$; (B) HMDMs: 1 control, 3 variant carriers, THP-1 $n = 4$; (C–E) THP-1 $n = 4$; (D) HMDMs: 2 controls, 4 variant carriers; (F–H) THP-1 $n = 4$.

See also Figure S4.

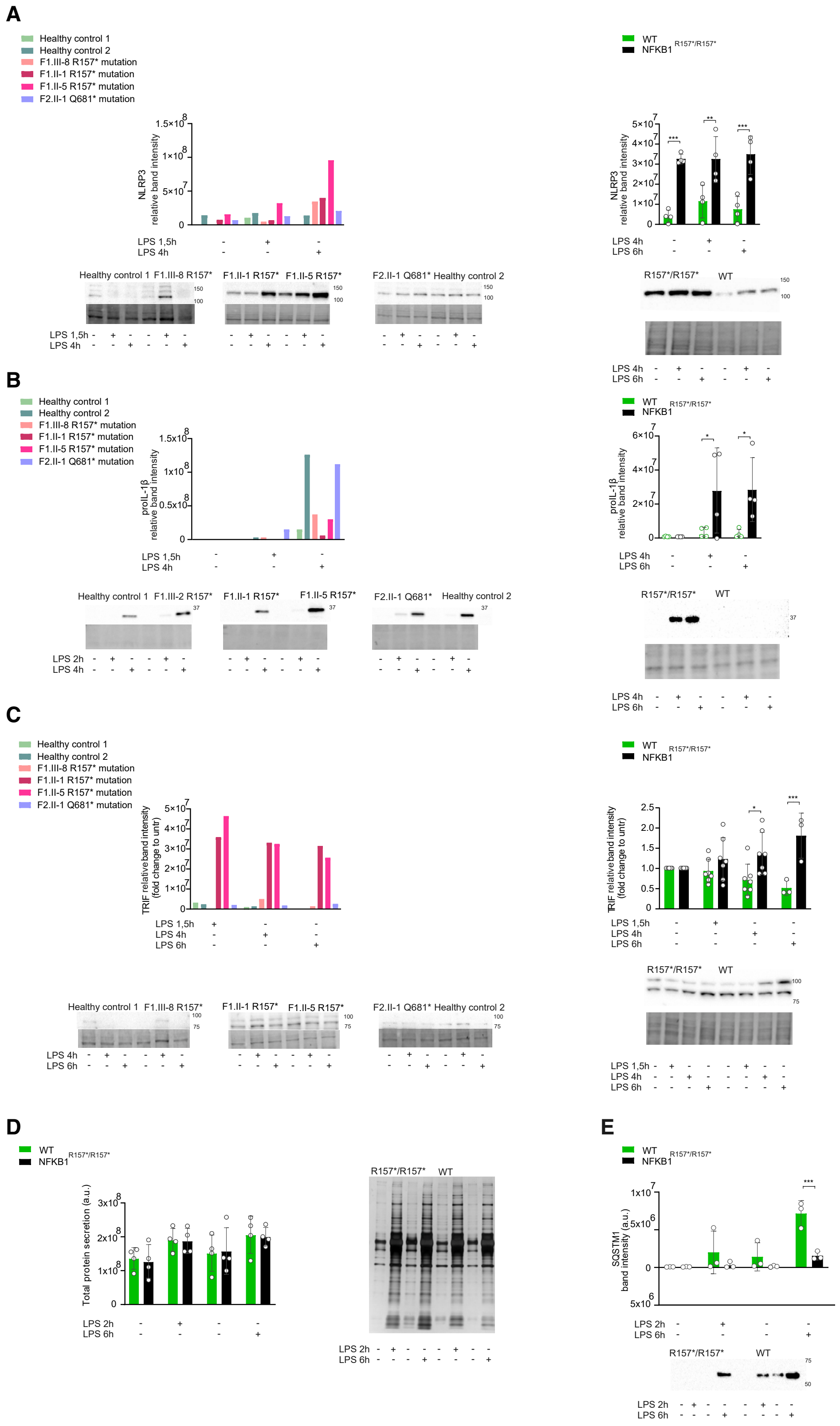

(legend on next page)

reaction was triggered by mechanical trauma or operation. Tissue injury results in release of damage-associated molecular patterns from cells and allows LPS to access the tissue, both of which activate the NLRP3 inflammasome. NLRP3 inflammasome activation is followed by IL-1β secretion, which is a primary trigger of the innate immune inflammatory response. IL-1β recruits neutrophils to the injury site and stimulates bone marrow neutrophil production,[41] compatible with the observed dense neutrophilic tissue infiltration and very high blood neutrophilia in patients. Activation of the NLRP3 inflammasome is accompanied by pyroptosis, a form of proinflammatory cell death mediated by GSDMD,[8] resulting in further release of active proinflammatory mediators. Extracellular LPS and mediators such as IL-1β and DNA released from pyroptotic cells are inducers of IFN-I production.[42] IFN-I has been previously shown to dampen activation of the NLRP3 inflammasome,[43] but blocking IFN-I signaling had no significant effect on IL-1β secretion in our experimental settings. As with the NLRP3 inflammasome, IFN-I has been linked to augmentation of proinflammatory cell death through various mechanisms including potentiating the effects of TNF and TNF-induced lethal shock in mice[44] and inducing receptor-interacting protein (RIP)1/RIP3 kinase-mediated necrotic cell death.[45] Thus, it is conceivable that in *NFKB1* variant carriers, the combined excessive activation of the NLRP3 inflammasome and IFN-I pathways triggered by trauma or minor infection predispose patients to life-threatening inflammatory responses and tissue necrosis.

NF-κB is a major hub of proinflammatory immune signaling, and therefore it is strictly controlled on several levels by post-translational modifications and inhibitory proteins.[7] LOF variants in genes that negatively regulate the activation of NF-κB, such as those encoding deubiquitinases *OTULIN* or *TNFAIP3*/A20, lead to uncontrolled inflammation, autoimmunity, and autoinflammatory diseases.[46–48] LOF variants of *NFKB1* typically lead to immune deficiency and/or autoinflammation. They represent the most frequent monogenic cause of common variable immunodeficiency, yet autoinflammation may be an underdiagnosed complication.[21,49,50] Two recent studies found that *NFKB1* is very intolerant to LOF variants disturbing the p65:p50 transcription factor function, and thus these variants are most likely pathogenic.[51,52] Lorenzini et al. described clinical manifestations of 157 patients carrying different LOF variants of *NFKB1*.[53] They found that while the most common manifestation was immunodeficiency, notably, more than half of the patients had autoimmune disease and one-third had autoinflammatory features. In our recent study, inflammatory manifestations were the dominating clinical feature in the Finnish cohort of *NFKB1* variant carriers.[21] These findings strongly suggest that NF-κB1 also activates inhibitory pathways to control proinflammatory signaling.

In mice, loss of *Nfkb1* causes multiple defects of innate and adaptive immune responses. *Nfkb1*$^{-/-}$ KO mice are more prone to bacterial and helminth infections, develop spontaneous inflammations and chronic inflammatory conditions, and show reduced numbers of myeloid progenitor cells and plasmacytoid dendritic cells.[54] Abnormal adaptive immune responses include dysfunctional B cells, aberrant immunoglobulin levels, and defective T cell responses.[54] In *Nfkb1*-deficient mice, both global p50 deletion and C-terminal (p105) deletion cause increased expression of IFN-β, ISGs, and TNF.[21,55–57] Reduced immunoglobulin levels and increased susceptibility to sterile inflammation have also been described in humans carrying *NFKB1* variants,[21,50,55,58] and here, we found increased expressions of TNF and ISGs in *NFKB1*-truncation-carrying cells. Consistent with this, increased transcription of ISGs, TNF, and other NF-κB-regulated cytokines have been previously reported in phorbol myristate acetate-differentiated *NFKB1* KO THP-1 macrophages.[59] We did not observe significant increases in expression of studied NF-κB target genes, suggesting that truncation of p50 or p105 does not increase transcription of the NLRP3 inflammasome components, which therefore cannot explain the increased NLRP3 inflammasome signaling. It is, however, possible that the reduced p50 level affects ISG expression, as p50:p50 homodimers have been shown to bind guanine-rich IFN response element-containing ISG promoters and repress their expression.[60] It is also plausible that reduced expression of M3K8, which is affected by deletion of p50 and p105, contributes to the enhanced TLR-induced IFN expression.[61]

NF-κB1 truncation selectively affected activation of different PRRs. We observed enhanced IL-1β secretion in p.R157*-variant-carrying cells following NLRP3 inflammasome priming via TLR4 and TLR1:2 and activation of the AIM2 inflammasome but not following activation of the noncanonical inflammasome. An augmented IFN-I response was apparent after TLR1:2 and -4 activation but not following activation of RIG-I/MDA-5, consistent with the data by Samie et al., who suggested that increased IFN-β expression in autophagy-deficient cells following LPS activation is primarily dependent on accumulation of TRIF.[34] Activated TLRs and RLR form signaling complexes using different components, in distinct subcellular sites, potentially explaining their different sensitivity to NF-κB1 variants.[34] Conventionally, RIG-I/MDA-5 signaling has been thought to be regulated by proteasomal degradation, and only recently was it shown that RIG-I/MDA-5 signaling can also be restricted by SQSTM1-dependent autophagy.[62] Thus, the phenotype of aberrant inflammasome and IFN-I activation required activation of specific pathways.

We observed significant accumulation of NLRP3, TRIF, and mtROS as well as increased levels of SQSTM1 and autophagosomal vesicles in patients' cells and in p.R157*-variant-carrying THP-1 monocytes, all implicating defective autophagic

Figure 6. Reduction of autophagy results in accumulation of key proteins of the NLRP3 inflammasome and interferon signaling

(A–C) HMDMs (left) and *NFKB1*$^{R157*/R157*}$ and WT THP-1 monocytes (right) were activated with LPS for indicated times, and the expression of (A) NLRP3, (B) pro-IL-1β, or (C) TRIF in cell lysates was blotted.

(D and E) *NFKB1*$^{R157*/R157*}$ and WT THP-1 monocytes were activated with LPS for indicate times and (D) total protein secretion was assessed by silver staining or (E) secretion of SQSTM1 was blotted from supernatant.

Quantifications, representative blots, and TPL (A–C) are shown. Two-way ANOVA followed by Šidak's test (A–E). The data are shown as mean ± SD. *$p < 0.05$, **$p < 0.01$, ***$p < 0.001$, ****$p < 0.0001$. (A–C) 2 controls, 4 variant carriers; THP-1 $n = 4$; (D) $n = 4$; (E) $n = 3$.

degradation. Enhanced ISG expression and dysregulated vesicular-related processes were confirmed by proteomics analysis, mainly in LPS-stimulated cells, further underlining that a stimulus is required for the exaggerated immune response. Interestingly, many lysosomal proteases were less expressed in p.R157*-variant-carrying cells, potentially impairing autophagic degradation. Secretory autophagy can compensate for impaired lysosome function,[37] but we did not observe compensational upregulation of secretory autophagy in p.R157*-variant-carrying cells; on the contrary, secretion of SQSTM1 was reduced. Selective targeting of NLRP3 and TRIF to autophagic degradation has been demonstrated as an important negative regulator of both NLRP3 inflammasome activation and IFN-I production.[12,13,34,36] Accordingly, stimulation of autophagy with rapamycin significantly reduced IL-1β secretion in *NFKB1*$^{R157\star/R157\star}$ THP-1 monocytes, whereas it had only a minor inhibitory effect on WT cells. Altogether, these results suggest that truncating variants of NF-κB1 reduce basal autophagy and impair autophagic degradation and secretory autophagy, resulting in the accumulation of principal components of the NLRP3 inflammasome and IFN pathways, thus licensing their increased activation.

Impaired autophagy has been implicated in excessive inflammatory reactions in autoinflammatory diseases.[63] In TNF receptor-associated periodic syndrome, accumulation of misfolded mutant TNFR1 protein was suggested to overload autophagy, thereby leading to the accumulation of enlarged autophagic vacuoles with undigested material.[64] In familial Mediterranean fever, pyrin has been shown to function also as an autophagy receptor that targets the inflammasome components NLRP3, NLRP1, and caspase-1 for degradation.[65] It was proposed that mutations of *MEFV* would interfere with the autophagic degradation of inflammasome components, thus leading to increased activation of the NLRP3 inflammasome.[63] In hyperimmunoglobulin D syndrome, impaired autophagic flux was suggested to result in an accumulation of damaged mitochondria and subsequent activation of the NLRP3 inflammasome.[66] Although the exact mechanisms by which loss of NF-κB1 compromises autophagy remain to be elucidated, our present results imply a significant role for NF-κB1 in the regulation of basal autophagy and autophagic degradation.

Six of the eight carriers of *NFKB1* truncating variants had developed a severe form of NF/PG, with low or absent bacterial growth in the affected tissue, which was grossly disproportional to the inflammatory reaction. The age of onset of patients' symptoms varied from newborn to more advanced age. We have previously identified several asymptomatic mutation carriers among patients' family members, indicating incomplete/variable penetrance.[21] However, a triggering event, such as trauma or surgery, often preceded the disease onset, which could explain the varying age and incomplete penetrance; thus, it is possible that asymptomatic mutation carriers have not encountered a triggering event. In tertiary healthcare, NF is not very uncommon, with reported incidence of 21 and 40 per million in the UK and US, respectively,[67,68] and therefore based on previously published case reports and our findings, it is important to establish possible genetic predisposition in its etiology. This is particularly important because patients have responded to immune-modulating therapies but not to antibiotics. Several patients described here have been successfully treated with glucocorticoids, IL-1β blockers, and JAK inhibitor. In patients with severe fasciitis with no or low bacterial burden and in patients with recurrent disease, genetic testing is warranted. In fasciitis patients with a known underlying truncating *NFKB1* mutation, anti-inflammatory therapies, including glucocorticoids and/or IL-1β targeting therapies or JAK inhibitors, could be beneficial in reducing excessive inflammation and the need for surgical revisions and amputations.

Limitations of the study

The main limitation of this study is the rather small number of patients with truncating *NFKB1* variants and limited patient sample availability. Small cohort size does not allow for the estimation of the significance of factors, such as the contribution of other genetic factors or lifestyle in the pathogenesis and penetrance of hyperinflammation, or the prevalence of truncating *NFKB1* variants in NF. We thoroughly studied the effect of the p.R157* variant and NF-κB1 p50 KO in the monocytic cell line but not the effects of NF-κB1 p105 KO.

STAR★METHODS

Detailed methods are provided in the online version of this paper and include the following:

- KEY RESOURCES TABLE
- RESOURCE AVAILABILITY
  - Lead contact
  - Materials availability
  - Data and code availability
- EXPERIMENTAL MODEL AND STUDY PARTICIPANT DETAILS
  - Ethics statement
  - Patient descriptions
- METHOD DETAILS
  - Genetic analyses
  - Culture of human monocyte-derived macrophages
  - Culture of THP-1 monocytes
  - Stimulation of human monocyte-derived macrophages and THP-1 monocytes
  - CRISPR-Cas9-mediated genome editing
  - Global proteomics analysis
  - Detection of secreted cytokines, LDH, mtROS, and autophagic vesicles
- QUANTITATIVE REAL-TIME PCR
- NANOSTRING ANALYSIS
  - Immunofluorescence analysis
  - Immunoblot and immunoprecipitation analysis
  - Interaction analysis
- QUANTIFICATION AND STATISTICAL ANALYSIS

SUPPLEMENTAL INFORMATION

Supplemental information can be found online at Mendeley Data: https://doi.org/10.1016/j.xcrm.2024.101503.

ACKNOWLEDGMENTS

This study was supported by grants from the Jane and Aatos Erkko Foundation (6347-6aed0 to K.K.E.); the Academy of Finland (322638 to K.K.E.); the

CellPress
OPEN ACCESS

Yrjö Jahnsson Foundation (20217434 and 20207306 to K.N.); the Päivikki and Sakari Sohlberg Foundation (K.N.); Finska Läkaresällskapet (K.K.E., D.C.N.); the Maud Kuistila Memorial Foundation (2020-0312C to K.N.); the Orion Research Foundation sr (K.S.); The Finnish Medical Foundation sr (K.S.); the Maire Lisko Foundation (K.R., K.N., K.S.); The Research Foundation of Rheumatic Diseases (K.R., K.N.); the Paulo Foundation (K.R.); the Stockmann's Foundation (K.K.E.); the Sigrid Jusélius Foundation; Finnish Foundation for Pediatric Research and Pediatric Research Center, Helsinki University Hospital (M.R.J.S.); the Care-for-Rare Foundation (C4R, 160073 to F.H.); Else Kröner-Fresenius Stiftung (EKFS, 2017_A110 to F.H.); and the German Federal Ministry of Education and Research (BMBF, 01GM1910C to F.H.). C.J.A.D. was funded by a Wellcome Clinical Research Career Development Fellowship (211153/Z/18/Z) and UK Medical Research Council Clinician Scientist Fellowship (MRX001598/1). We are grateful to the following projects and research service core facilities: the Genomics of Rare Immune Disorders (GRID) Project for diagnostic testing in patient F6.II-1, the Institute of Molecular Medicine Finland Technology Center, the UH Biomedicum Imaging Unit, the UH Biomedicum Flow Cytometry Core, and the UH Library for funding of open access.

AUTHOR CONTRIBUTIONS

Conceptualization, K.N., K.S., S.K., M.V., G.B., M.R.J.S., and K.K.E.; writing – original draft, K.N., K.S., K.R., G.B., M.R.J.S., and K.K.E.; methodology, validation, formal analysis, data curation, and visualization, K.N., K.S., S.K., K.R., S.J., R.M., M. Kaustio, M. Kinnunen, and G.B.; investigation, K.N., K.S., S.K., K.R., V.-P.K., S.P.D.-J., C.J.A.D., J.S., J.P., A.B., M.B., M. Kaustio, M. Kinnunen, C.K., F.H., O. Kilpivaara, and G.B.; resources, K.W., E.I.N., S.P.D.-J., C.J.A.D., O. Kuismin, J.S., I.R., T.M., S.M., D.C.N., U.W.-K., C.K., F.H., T.J., T.H., M.V., M.R.J.S., and K.K.E.; supervision and project administration, K.N., G.B., and K.K.E. All authors critically reviewed and edited the manuscript.

DECLARATION OF INTERESTS

C.J.A.D. has provided consultative advice to Synairgen on behalf of Newcastle University.

Received: December 14, 2022
Revised: January 4, 2024
Accepted: March 18, 2024
Published: April 8, 2024

REFERENCES

1. Manthiram, K., Zhou, Q., Aksentijevich, I., and Kastner, D.L. (2017). The monogenic autoinflammatory diseases define new pathways in human innate immunity and inflammation. Nat. Immunol. *18*, 832–842. https://doi.org/10.1038/ni.3777.
2. de Jesus, A.A., Hou, Y., Brooks, S., Malle, L., Biancotto, A., Huang, Y., Calvo, K.R., Marrero, B., Moir, S., Oler, A.J., et al. (2020). Distinct interferon signatures and cytokine patterns define additional systemic autoinflammatory diseases. J. Clin. Invest. *130*, 1669–1682. https://doi.org/10.1172/JCI129301.
3. Pathak, S., McDermott, M.F., and Savic, S. (2017). Autoinflammatory diseases: update on classification diagnosis and management. J. Clin. Pathol. *70*, 1–8. https://doi.org/10.1136/jclinpath-2016-203810.
4. Oeckinghaus, A., and Ghosh, S. (2009). The NF-kappaB family of transcription factors and its regulation. Cold Spring Harb. Perspect. Biol. *1*, a000034. https://doi.org/10.1101/cshperspect.a000034.
5. Zhang, Q., Lenardo, M.J., and Baltimore, D. (2017). 30 Years of NF-kappaB: A Blossoming of Relevance to Human Pathobiology. Cell *168*, 37–57. https://doi.org/10.1016/j.cell.2016.12.012.
6. Liu, T., Zhang, L., Joo, D., and Sun, S.C. (2017). NF-kappaB signaling in inflammation. Signal Transduct. Target. Ther. *2*, 17023. https://doi.org/10.1038/sigtrans.2017.23.
7. Afonina, I.S., Zhong, Z., Karin, M., and Beyaert, R. (2017). Limiting inflammation-the negative regulation of NF-kappaB and the NLRP3 inflammasome. Nat. Immunol. *18*, 861–869. https://doi.org/10.1038/ni.3772.
8. Christgen, S., Place, D.E., and Kanneganti, T.D. (2020). Toward targeting inflammasomes: insights into their regulation and activation. Cell Res. *30*, 315–327. https://doi.org/10.1038/s41422-020-0295-8.
9. Bauernfeind, F.G., Horvath, G., Stutz, A., Alnemri, E.S., MacDonald, K., Speert, D., Fernandes-Alnemri, T., Wu, J., Monks, B.G., Fitzgerald, K.A., et al. (2009). Cutting edge: NF-kappaB activating pattern recognition and cytokine receptors license NLRP3 inflammasome activation by regulating NLRP3 expression. J. Immunol. *183*, 787–791. https://doi.org/10.4049/jimmunol.0901363.
10. Zhong, Z., Umemura, A., Sanchez-Lopez, E., Liang, S., Shalapour, S., Wong, J., He, F., Boassa, D., Perkins, G., Ali, S.R., et al. (2016). NF-kappaB Restricts Inflammasome Activation via Elimination of Damaged Mitochondria. Cell *164*, 896–910. https://doi.org/10.1016/j.cell.2015.12.057.
11. Deretic, V., and Levine, B. (2018). Autophagy balances inflammation in innate immunity. Autophagy *14*, 243–251. https://doi.org/10.1080/15548627.2017.1402992.
12. Shi, C.S., Shenderov, K., Huang, N.N., Kabat, J., Abu-Asab, M., Fitzgerald, K.A., Sher, A., and Kehrl, J.H. (2012). Activation of autophagy by inflammatory signals limits IL-1beta production by targeting ubiquitinated inflammasomes for destruction. Nat. Immunol. *13*, 255–263. https://doi.org/10.1038/ni.2215.
13. Harris, J., Hartman, M., Roche, C., Zeng, S.G., O'Shea, A., Sharp, F.A., Lambe, E.M., Creagh, E.M., Golenbock, D.T., Tschopp, J., et al. (2011). Autophagy controls IL-1beta secretion by targeting pro-IL-1beta for degradation. J. Biol. Chem. *286*, 9587–9597. https://doi.org/10.1074/jbc.M110.202911.
14. Stevens, D.L., and Bryant, A.E. (2017). Necrotizing Soft-Tissue Infections. N. Engl. J. Med. *377*, 2253–2265. https://doi.org/10.1056/NEJMra1600673.
15. Kaustio, M., Haapaniemi, E., Göös, H., Hautala, T., Park, G., Syrjänen, J., Einarsdottir, E., Sahu, B., Kilpinen, S., Rounioja, S., et al. (2017). Damaging heterozygous mutations in NFKB1 lead to diverse immunologic phenotypes. J. Allergy Clin. Immunol. *140*, 782–796. https://doi.org/10.1016/j.jaci.2016.10.054.
16. Fang, R., Wang, J., Jiang, X.Y., Wang, S.H., Cheng, H., and Zhou, Q. (2021). Case Report: A Novel Mutation in NFKB1 Associated With Pyoderma Gangrenosum. Front. Genet. *12*, 673453. https://doi.org/10.3389/fgene.2021.673453.
17. Fliegauf, M., Bryant, V.L., Frede, N., Slade, C., Woon, S.T., Lehnert, K., Winzer, S., Bulashevska, A., Scerri, T., Leung, E., et al. (2015). Haploinsufficiency of the NF-kappaB1 Subunit p50 in Common Variable Immunodeficiency. Am. J. Hum. Genet. *97*, 389–403. https://doi.org/10.1016/j.ajhg.2015.07.008.
18. Sun, Q., Gehlhausen, J.R., Freudzon, M., Kibbi, N., Bale, A., Choate, K., Tomayko, M., Odell, I., and Ramachandran, S. (2021). A novel NFkB1 mutation linking pyoderma gangrenosum and common variable immunodeficiency. JAAD Case Rep. *18*, 61–63. https://doi.org/10.1016/j.jdcr.2021.10.015.
19. Nguyen, D.T.I., Grimes, A., Mahoney, D., Faro, S., Shearer, W.T., Miller, A.L., and Rider, N.L. (2021). Case Report: Post-Partum Complications of NFkappaB1 Deficiency Underscore a Need to Better Understand Primary Immunodeficiency Management During Pregnancy. Front. Pediatr. *9*, 648022. https://doi.org/10.3389/fped.2021.648022.
20. Bergbreiter, A., Jaeger, T., Karle, A., Bitzinger, D., Ettl, T., Spanier, G., Jägle, H., Neu, R., Söder, Y., Evert, M., et al. (2021). Recurrent necrotizing cellulitis, multi-organ autoimmune disease and humoral immunodeficiency due to a novel NFKB1 frameshift mutation. Eur. J. Med. Genet. *64*, 104144. https://doi.org/10.1016/j.ejmg.2021.104144.
21. Tuovinen, E.A., Kuismin, O., Soikkonen, L., Martelius, T., Kaustio, M., Hämäläinen, S., Viskari, H., Syrjänen, J., Wartiovaara-Kautto, U., Eklund, K.K., et al. (2023). Long-term follow up of families with pathogenic

NFKB1 variants reveals incomplete penetrance and frequent inflammatory sequelae. Clin. Immunol. *246*, 109181. https://doi.org/10.1016/j.clim.2022.109181.

22. Santaniemi, W., Åström, P., Glumoff, V., Pernaa, N., Tallgren, E.N., Palosaari, S., Nissinen, A., Kaustio, M., Kuismin, O., Saarela, J., et al. (2023). Inflammation and Neutrophil Oxidative Burst in a Family with NFKB1 p.R157X LOF and Sterile Necrotizing Fasciitis. J. Clin. Immunol. *43*, 1007–1018. https://doi.org/10.1007/s10875-023-01461-3.
23. Lamkanfi, M., and Dixit, V.M. (2014). Mechanisms and functions of inflammasomes. Cell *157*, 1013–1022. https://doi.org/10.1016/j.cell.2014.04.007.
24. Takeda, K., and Akira, S. (2004). TLR signaling pathways. Semin. Immunol. *16*, 3–9. https://doi.org/10.1016/j.smim.2003.10.003.
25. Oosenbrug, T., van de Graaff, M.J., Haks, M.C., van Kasteren, S., and Ressing, M.E. (2020). An alternative model for type I interferon induction downstream of human TLR2. J. Biol. Chem. *295*, 14325–14342. https://doi.org/10.1074/jbc.RA120.015283.
26. Liu, X., Salokas, K., Tamene, F., Jiu, Y., Weldatsadik, R.G., Öhman, T., and Varjosalo, M. (2018). An AP-MS- and BioID-compatible MAC-tag enables comprehensive mapping of protein interactions and subcellular localizations. Nat. Commun. *9*, 1188. https://doi.org/10.1038/s41467-018-03523-2.
27. Pereira, S.G., and Oakley, F. (2008). Nuclear factor-kappaB1: regulation and function. Int. J. Biochem. Cell Biol. *40*, 1425–1430. https://doi.org/10.1016/j.biocel.2007.05.004.
28. De Tito, S., Hervás, J.H., van Vliet, A.R., and Tooze, S.A. (2020). The Golgi as an Assembly Line to the Autophagosome. Trends Biochem. Sci. *45*, 484–496. https://doi.org/10.1016/j.tibs.2020.03.010.
29. Mitchell, S., Vargas, J., and Hoffmann, A. (2016). Signaling via the NFkappaB system. Wiley Interdiscip. Rev. Syst. Biol. Med. *8*, 227–241. https://doi.org/10.1002/wsbm.1331.
30. Dan Dunn, J., Alvarez, L.A., Zhang, X., and Soldati, T. (2015). Reactive oxygen species and mitochondria: A nexus of cellular homeostasis. Redox Biol. *6*, 472–485. https://doi.org/10.1016/j.redox.2015.09.005.
31. Nakahira, K., Haspel, J.A., Rathinam, V.A.K., Lee, S.J., Dolinay, T., Lam, H.C., Englert, J.A., Rabinovitch, M., Cernadas, M., Kim, H.P., et al. (2011). Autophagy proteins regulate innate immune responses by inhibiting the release of mitochondrial DNA mediated by the NALP3 inflammasome. Nat. Immunol. *12*, 222–230. https://doi.org/10.1038/ni.1980.
32. Anding, A.L., and Baehrecke, E.H. (2017). Cleaning House: Selective Autophagy of Organelles. Dev. Cell *41*, 10–22. https://doi.org/10.1016/j.devcel.2017.02.016.
33. Klionsky, D.J., Abdel-Aziz, A.K., Abdelfatah, S., Abdellatif, M., Abdoli, A., Abel, S., Abeliovich, H., Abildgaard, M.H., Abudu, Y.P., Acevedo-Arozena, A., et al. (2021). Guidelines for the use and interpretation of assays for monitoring autophagy *17*, 1–382, 17. https://doi.org/10.1080/15548627.2020.1797280.
34. Samie, M., Lim, J., Verschueren, E., Baughman, J.M., Peng, I., Wong, A., Kwon, Y., Senbabaoglu, Y., Hackney, J.A., Keir, M., et al. (2018). Selective autophagy of the adaptor TRIF regulates innate inflammatory signaling. Nat. Immunol. *19*, 246–254. https://doi.org/10.1038/s41590-017-0042-6.
35. Mehto, S., Jena, K.K., Nath, P., Chauhan, S., Kolapalli, S.P., Das, S.K., Sahoo, P.K., Jain, A., Taylor, G.A., and Chauhan, S. (2019). The Crohn's Disease Risk Factor IRGM Limits NLRP3 Inflammasome Activation by Impeding Its Assembly and by Mediating Its Selective Autophagy. Mol. Cell *73*, 429–445.e7. https://doi.org/10.1016/j.molcel.2018.11.018.
36. Saitoh, T., Fujita, N., Jang, M.H., Uematsu, S., Yang, B.G., Satoh, T., Omori, H., Noda, T., Yamamoto, N., Komatsu, M., et al. (2008). Loss of the autophagy protein Atg16L1 enhances endotoxin-induced IL-1beta production. Nature *456*, 264–268. https://doi.org/10.1038/nature07383.
37. Solvik, T.A., Nguyen, T.A., Tony Lin, Y.H., Marsh, T., Huang, E.J., Wiita, A.P., Debnath, J., and Leidal, A.M. (2022). Secretory autophagy maintains proteostasis upon lysosome inhibition. J. Cell Biol. *221*, e202110151. https://doi.org/10.1083/jcb.202110151.
38. Gonzalez, C.D., Resnik, R., and Vaccaro, M.I. (2020). Secretory Autophagy and Its Relevance in Metabolic and Degenerative Disease. Front. Endocrinol. *11*, 266. https://doi.org/10.3389/fendo.2020.00266.
39. de Vasconcelos, N.M., and Lamkanfi, M. (2020). Recent Insights on Inflammasomes, Gasdermin Pores, and Pyroptosis. Cold Spring Harb. Perspect. Biol. *12*, a036392. https://doi.org/10.1101/cshperspect.a036392.
40. Stutz, A., Kolbe, C.C., Stahl, R., Horvath, G.L., Franklin, B.S., van Ray, O., Brinkschulte, R., Geyer, M., Meissner, F., and Latz, E. (2017). NLRP3 inflammasome assembly is regulated by phosphorylation of the pyrin domain. J. Exp. Med. *214*, 1725–1736. https://doi.org/10.1084/jem.20160933.
41. Dinarello, C.A. (2011). A clinical perspective of IL-1beta as the gatekeeper of inflammation. Eur. J. Immunol. *41*, 1203–1217. https://doi.org/10.1002/eji.201141550.
42. Aarreberg, L.D., Wilkins, C., Ramos, H.J., Green, R., Davis, M.A., Chow, K., and Gale, M., Jr. (2018). Interleukin-1beta Signaling in Dendritic Cells Induces Antiviral Interferon Responses. mBio *9*, e00342–18. https://doi.org/10.1128/mBio.00342-18.
43. Mayer-Barber, K.D., and Yan, B. (2017). Clash of the Cytokine Titans: counter-regulation of interleukin-1 and type I interferon-mediated inflammatory responses. Cell. Mol. Immunol. *14*, 22–35. https://doi.org/10.1038/cmi.2016.25.
44. Huys, L., Van Hauwermeiren, F., Dejager, L., Dejonckheere, E., Lienenklaus, S., Weiss, S., Leclercq, G., and Libert, C. (2009). Type I interferon drives tumor necrosis factor-induced lethal shock. J. Exp. Med. *206*, 1873–1882. https://doi.org/10.1084/jem.20090213.
45. Thapa, R.J., Nogusa, S., Chen, P., Maki, J.L., Lerro, A., Andrake, M., Rall, G.F., Degterev, A., and Balachandran, S. (2013). Interferon-induced RIP1/RIP3-mediated necrosis requires PKR and is licensed by FADD and caspases. Proc. Natl. Acad. Sci. USA *110*, E3109–E3118. https://doi.org/10.1073/pnas.1301218110.
46. Lork, M., Verhelst, K., and Beyaert, R. (2017). CYLD, A20 and OTULIN deubiquitinases in NF-kappaB signaling and cell death: so similar, yet so different. Cell Death Differ. *24*, 1172–1183. https://doi.org/10.1038/cdd.2017.46.
47. Zhou, Q., Wang, H., Schwartz, D.M., Stoffels, M., Park, Y.H., Zhang, Y., Yang, D., Demirkaya, E., Takeuchi, M., Tsai, W.L., et al. (2016). Loss-of-function mutations in TNFAIP3 leading to A20 haploinsufficiency cause an early-onset autoinflammatory disease. Nat. Genet. *48*, 67–73. https://doi.org/10.1038/ng.3459.
48. Zhou, Q., Yu, X., Demirkaya, E., Deuitch, N., Stone, D., Tsai, W.L., Kuehn, H.S., Wang, H., Yang, D., Park, Y.H., et al. (2016). Biallelic hypomorphic mutations in a linear deubiquitinase define otulipenia, an early-onset autoinflammatory disease. Proc. Natl. Acad. Sci. USA *113*, 10127–10132. https://doi.org/10.1073/pnas.1612594113.
49. Fliegauf, M., and Grimbacher, B. (2018). Nuclear factor kappaB mutations in human subjects: The devil is in the details. J. Allergy Clin. Immunol. *142*, 1062–1065. https://doi.org/10.1016/j.jaci.2018.06.050.
50. Tuijnenburg, P., Lango Allen, H., Burns, S.O., Greene, D., Jansen, M.H., Staples, E., Stephens, J., Carss, K.J., Biasci, D., Baxendale, H., et al. (2018). Loss-of-function nuclear factor kappaB subunit 1 (NFKB1) variants are the most common monogenic cause of common variable immunodeficiency in Europeans. J. Allergy Clin. Immunol. *142*, 1285–1296. https://doi.org/10.1016/j.jaci.2018.01.039.
51. Li, J., Lei, W.T., Zhang, P., Rapaport, F., Seeleuthner, Y., Lyu, B., Asano, T., Rosain, J., Hammadi, B., Zhang, Y., et al. (2021). Biochemically deleterious human NFKB1 variants underlie an autosomal dominant form of common variable immunodeficiency. J. Exp. Med. *218*, e20210566. https://doi.org/10.1084/jem.20210566.
52. Fliegauf, M., Kinnunen, M., Posadas-Cantera, S., Camacho-Ordonez, N., Abolhassani, H., Alsina, L., Atschekzei, F., Bogaert, D.J., Burns, S.O.,

CellPress
OPEN ACCESS

Church, J.A., et al. (2022). Detrimental NFKB1 missense variants affecting the Rel-homology domain of p105/p50. Front. Immunol. *13*, 965326. https://doi.org/10.3389/fimmu.2022.965326.

53. Lorenzini, T., Fliegauf, M., Klammer, N., Frede, N., Proietti, M., Bulashevska, A., Camacho-Ordonez, N., Varjosalo, M., Kinnunen, M., de Vries, E., et al. (2020). Characterization of the clinical and immunologic phenotype and management of 157 individuals with 56 distinct heterozygous NFKB1 mutations. J. Allergy Clin. Immunol. *146*, 901–911. https://doi.org/10.1016/j.jaci.2019.11.051.

54. Cartwright, T., Perkins, N.D., and L Wilson, C. (2016). NFKB1: a suppressor of inflammation, ageing and cancer. FEBS J. *283*, 1812–1822. https://doi.org/10.1111/febs.13627.

55. Ishikawa, H., Claudio, E., Dambach, D., Raventós-Suárez, C., Ryan, C., and Bravo, R. (1998). Chronic inflammation and susceptibility to bacterial infections in mice lacking the polypeptide (p)105 precursor (NF-kappaB1) but expressing p50. J. Exp. Med. *187*, 985–996. https://doi.org/10.1084/jem.187.7.985.

56. Sha, W.C., Liou, H.C., Tuomanen, E.I., and Baltimore, D. (1995). Targeted disruption of the p50 subunit of NF-kappa B leads to multifocal defects in immune responses. Cell *80*, 321–330. https://doi.org/10.1016/0092-8674(95)90415-8.

57. Ben-Addi, A., Mambole-Dema, A., Brender, C., Martin, S.R., Janzen, J., Kjaer, S., Smerdon, S.J., and Ley, S.C. (2014). IkappaB kinase-induced interaction of TPL-2 kinase with 14-3-3 is essential for Toll-like receptor activation of ERK-1 and -2 MAP kinases. Proc. Natl. Acad. Sci. USA *111*, E2394–E2403. https://doi.org/10.1073/pnas.1320440111.

58. Fliegauf, M., Krüger, R., Steiner, S., Hanitsch, L.G., Büchel, S., Wahn, V., von Bernuth, H., and Grimbacher, B. (2021). A Pathogenic Missense Variant in NFKB1 Causes Common Variable Immunodeficiency Due to Detrimental Protein Damage. Front. Immunol. *12*, 621503. https://doi.org/10.3389/fimmu.2021.621503.

59. Somma, D., Kok, F.O., Kerrigan, D., Wells, C.A., and Carmody, R.J. (2021). Defining the Role of Nuclear Factor (NF)-kappaB p105 Subunit in Human Macrophage by Transcriptomic Analysis of NFKB1 Knockout THP1 Cells. Front. Immunol. *12*, 669906. https://doi.org/10.3389/fimmu.2021.669906.

60. Cheng, C.S., Feldman, K.E., Lee, J., Verma, S., Huang, D.B., Huynh, K., Chang, M., Ponomarenko, J.V., Sun, S.C., Benedict, C.A., et al. (2011). The specificity of innate immune responses is enforced by repression of interferon response elements by NF-kappaB p50. Sci. Signal. *4*, ra11. https://doi.org/10.1126/scisignal.2001501.

61. Kaiser, F., Cook, D., Papoutsopoulou, S., Rajsbaum, R., Wu, X., Yang, H.T., Grant, S., Ricciardi-Castagnoli, P., Tsichlis, P.N., Ley, S.C., and O'Garra, A. (2009). TPL-2 negatively regulates interferon-beta production in macrophages and myeloid dendritic cells. J. Exp. Med. *206*, 1863–1871. https://doi.org/10.1084/jem.20091059.

62. Du, Y., Duan, T., Feng, Y., Liu, Q., Lin, M., Cui, J., and Wang, R.F. (2018). LRRC25 inhibits type I IFN signaling by targeting ISG15-associated RIG-I for autophagic degradation. EMBO J. *37*, 351–366. https://doi.org/10.15252/embj.201796781.

63. Hua, Y., Shen, M., McDonald, C., and Yao, Q. (2018). Autophagy dysfunction in autoinflammatory diseases. J. Autoimmun. *88*, 11–20. https://doi.org/10.1016/j.jaut.2017.10.012.

64. Bachetti, T., Chiesa, S., Castagnola, P., Bani, D., Di Zanni, E., Omenetti, A., D'Osualdo, A., Fraldi, A., Ballabio, A., Ravazzolo, R., et al. (2013). Autophagy contributes to inflammation in patients with TNFR-associated periodic syndrome (TRAPS). Ann. Rheum. Dis. *72*, 1044–1052. https://doi.org/10.1136/annrheumdis-2012-201952.

65. Kimura, T., Jain, A., Choi, S.W., Mandell, M.A., Schroder, K., Johansen, T., and Deretic, V. (2015). TRIM-mediated precision autophagy targets cytoplasmic regulators of innate immunity. J. Cell Biol. *210*, 973–989. https://doi.org/10.1083/jcb.201503023.

66. van der Burgh, R., Ter Haar, N.M., Boes, M.L., and Frenkel, J. (2013). Mevalonate kinase deficiency, a metabolic autoinflammatory disease. Clin. Immunol. *147*, 197–206. https://doi.org/10.1016/j.clim.2012.09.011.

67. Bodansky, D.M.S., Begaj, I., Evison, F., Webber, M., Woodman, C.B., and Tucker, O.N. (2020). A 16-year Longitudinal Cohort Study of Incidence and Bacteriology of Necrotising Fasciitis in England. World J. Surg. *44*, 2580–2591. https://doi.org/10.1007/s00268-020-05559-2.

68. Arif, N., Yousfi, S., and Vinnard, C. (2016). Deaths from necrotizing fasciitis in the United States, 2003-2013. Epidemiol. Infect. *144*, 1338–1344. https://doi.org/10.1017/S0950268815002745.

69. Zudaire, E., Gambardella, L., Kurcz, C., and Vermeren, S. (2011). A computational tool for quantitative analysis of vascular networks. PLoS One *6*, e27385. https://doi.org/10.1371/journal.pone.0027385.

70. Wang, K., Li, M., and Hakonarson, H. (2010). ANNOVAR: functional annotation of genetic variants from high-throughput sequencing data. Nucleic Acids Res. *38*, e164. https://doi.org/10.1093/nar/gkq603.

71. Li, H., and Durbin, R. (2010). Fast and accurate long-read alignment with Burrows-Wheeler transform. Bioinformatics *26*, 589–595. https://doi.org/10.1093/bioinformatics/btp698.

72. Van der Auwera, G.A., Carneiro, M.O., Hartl, C., Poplin, R., Del Angel, G., Levy-Moonshine, A., Jordan, T., Shakir, K., Roazen, D., Thibault, J., et al. (2013). From FastQ data to high confidence variant calls: the Genome Analysis Toolkit best practices pipeline. Curr. Protoc. Bioinformatics *43*, 11.10.1–11 10 33. https://doi.org/10.1002/0471250953.bi1110s43.

73. reneshbedre/bioinfokit: Bioinformatics data analysis and visualization toolkit (2.0.9). Zenodo. https://doi.org/10.5281/zenodo.3964972.

74. Lazar, C., Gatto, L., Ferro, M., Bruley, C., and Burger, T. (2016). Accounting for the Multiple Natures of Missing Values in Label-Free Quantitative Proteomics Data Sets to Compare Imputation Strategies. J. Proteome Res. *15*, 1116–1125. https://doi.org/10.1021/acs.jproteome.5b00981.

75. Chen, S., Francioli, L.C., Goodrich, J.K., Collins, R.L., Kanai, M., Wang, Q., Alföldi, J., Watts, N.A., Vittal, C., Gauthier, L.D., et al. (2022). A genome-wide mutational constraint map quantified from variation in 76,156 human genomes. Preprint at bioRxiv. https://doi.org/10.1101/2022.03.20.485034.

76. Jaganathan, K., Kyriazopoulou Panagiotopoulou, S., McRae, J.F., Darbandi, S.F., Knowles, D., Li, Y.I., Kosmicki, J.A., Arbelaez, J., Cui, W., Schwartz, G.B., et al. (2019). Predicting splicing from primary sequence with deep learning. Cell *176*, 535–548.e24. https://doi.org/10.1016/j.cell.2018.12.015.

77. Cingolani, P., Platts, A., Wang, L.L., Coon, M., Nguyen, T., Wang, L., Land, S.J., Lu, X., and Ruden, D.M. (2012). A program for annotating and predicting the effects of single nucleotide polymorphisms, SnpEff: SNPs in the genome of Drosophila melanogaster strain w1118. Fly (Austin) *6*, 80–92. https://doi.org/10.4161/fly.19695 iso-2; iso-3. .

78. Li, Q., and Wang, K. (2017). InterVar: Clinical Interpretation of Genetic Variants by the 2015 ACMG-AMP Guidelines. Am. J. Hum. Genet. *100*, 267–280. https://doi.org/10.1016/j.ajhg.2017.01.004.

79. Richards, S., Aziz, N., Bale, S., Bick, D., Das, S., Gastier-Foster, J., Grody, W.W., Hegde, M., Lyon, E., Spector, E., et al. (2015). Standards and guidelines for the interpretation of sequence variants: a joint consensus recommendation of the American college of medical genetics and genomics and the association for molecular pathology. Genet. Med. *17*, 405–424. https://doi.org/10.1038/gim.2015.3.

80. Simeoni, I., Shamardina, O., Deevi, S.V., Thomas, M., Megy, K., Staples, E., Whitehorn, D., Duarte, D., Mapeta, R., Ouwehand, W.H., et al. (2019). GRID – Genomics of Rare Immune Disorders: a highly sensitive and specific diagnostic gene panel for patients with primary immunodeficiencies. Preprint at bioRxiv, 431544. https://doi.org/10.1101/431544.

81. Wartiovaara-Kautto, U., Hirvonen, E.A.M., Pitkänen, E., Heckman, C., Saarela, J., Kettunen, K., Porkka, K., and Kilpivaara, O. (2018). Germline alterations in a consecutive series of acute myeloid leukemia.

Leukemia *32*, 2282–2285. https://doi.org/10.1038/s41375-018-0049-5.

82. Scanu, A., Oliviero, F., Gruaz, L., Sfriso, P., Pozzuoli, A., Frezzato, F., Agostini, C., Burger, D., and Punzi, L. (2010). High-density lipoproteins downregulate CCL2 production in human fibroblast-like synoviocytes stimulated by urate crystals. Arthritis Res. Ther. *12*, R23. https://doi.org/10.1186/ar2930.

83. Xu, L., Liu, Y., and Han, R. (2019). BEAT: A Python Program to Quantify Base Editing from Sanger Sequencing. CRISPR J. *2*, 223–229. https://doi.org/10.1089/crispr.2019.0017.

84. Meier, F., Brunner, A.D., Koch, S., Koch, H., Lubeck, M., Krause, M., Goedecke, N., Decker, J., Kosinski, T., Park, M.A., et al. (2018). Online Parallel Accumulation-Serial Fragmentation (PASEF) with a Novel Trapped Ion Mobility Mass Spectrometer. Mol. Cell. Proteomics *17*, 2534–2545. https://doi.org/10.1074/mcp.TIR118.000900.

85. Meier, F., Brunner, A.D., Frank, M., Ha, A., Bludau, I., Voytik, E., Kaspar-Schoenefeld, S., Lubeck, M., Raether, O., Bache, N., et al. (2020). diaPASEF: parallel accumulation-serial fragmentation combined with data-independent acquisition. Nat. Methods *17*, 1229–1236. https://doi.org/10.1038/s41592-020-00998-0.

86. Demichev, V., Messner, C.B., Vernardis, S.I., Lilley, K.S., and Ralser, M. (2020). DIA-NN: neural networks and interference correction enable deep proteome coverage in high throughput. Nat. Methods *17*, 41–44. https://doi.org/10.1038/s41592-019-0638-x.

87. Demichev, V., Szyrwiel, L., Yu, F., Teo, G.C., Rosenberger, G., Niewienda, A., Ludwig, D., Decker, J., Kaspar-Schoenefeld, S., Lilley, K.S., et al. (2022). dia-PASEF data analysis using FragPipe and DIA-NN for deep proteomics of low sample amounts. Nat. Commun. *13*, 3944. https://doi.org/10.1038/s41467-022-31492-0.

88. Keskitalo, S., Haapaniemi, E., Einarsdottir, E., Rajamäki, K., Heikkilä, H., Ilander, M., Pöyhönen, M., Morgunova, E., Hokynar, K., Lagström, S., et al. (2019). Novel TMEM173 Mutation and the Role of Disease Modifying Alleles. Front. Immunol. *10*, 2770. https://doi.org/10.3389/fimmu.2019.02770.

89. Moritz, C.P. (2017). Tubulin or Not Tubulin: Heading Toward Total Protein Staining as Loading Control in Western Blots. Proteomics *17*. https://doi.org/10.1002/pmic.201600189.

90. O'Connell, K.L., and Stults, J.T. (1997). Identification of mouse liver proteins on two-dimensional electrophoresis gels by matrix-assisted laser desorption/ionization mass spectrometry of in situ enzymatic digests. Electrophoresis *18*, 349–359. https://doi.org/10.1002/elps.1150180309.

CellPress
OPEN ACCESS

STAR★METHODS

KEY RESOURCES TABLE

REAGENT or RESOURCE	SOURCE	IDENTIFIER
Antibodies		
p65	Abcam	Cat#ab7970; RRID: AB_306184
p65	Abcam	Cat#ab140751; RRID: AB_2922817
p50	Biosite	Cat#616702; RRID: AB_315856
p52	Abcam	Cat#ab31409; RRID: AB_881289
LC3	Novus Biologicals	Cat# NB100-2331; RRID: AB_10001955
NF-κB1 (p105/p50)	Cell Signaling Technology	Cat#3035; RRID: AB_330564
TICAM-1	Nordic Biosite	Cat#657102; RRID: AB_2562543
IL-1β	Santa Cruz Biotechnology	Cat#sc-7884; RRID: AB_2124476
NLRP3	AdipoGen	Cat#AG-20B-0014; RRID: AB_2490202
ASC	Adipogen	AL177, Cat#AG-25B0006-C100; RRID: AB_2885200
SQSTM1	Abcam	Cat#ab56416; RRID: AB_945626
GAPDH	Cell Signaling Technology	Cat#5174; RRID: AB_10622025
IFNAR2	Millipore	Cat#MAB1155; RRID: AB_2122758
anti-mouse Alexa IgG 488	Invitrogen	Cat#A11029; RRID: AB_2534088
anti-rabbit Alexa IgG 488	Invitrogen	Cat#A11008; RRID: AB_143165
Mouse IgG1	Dako	Cat#X0931; RRID: AB_2889134
Normal rabbit IgG control	R&D Systems	Cat#AB-105-C; RRID: AB_354266
Biological samples		
Peripheral blood from individuals	N/A	N/A
Experimental models: Cell Lines		
THP1-Dual Cells	Invivogen	Cat#thpd-nfis, RRID:CVCL_X599
Chemicals, peptides, and recombinant proteins		
10x Lysis buffer	Cell Signaling	Cat#9803S
cOmplete EASYpack Protease Inhibitor Coctail	Roche	Cat#04693116001
Phosphatase Inhibitor	Thermo Scientific	Cat#A32957
4x Laemmli Sample Buffer	Bio-Rad	Cat#161-0747
N,N,N′,N′-Tetramethyl-ethylenediamine (Temed)	Sigma Aldrich	Cat#T9281
Ammonium Persulfate (APS)	Thermo Scientific	Cat#17874
10x Tris/Glycine/SDS Buffer	Bio-Rad	Cat#1610772
Clarity Western ECL Substrate	Bio-Rad	Cat#170-5061
Silver nitrate	Merk	Cat#101512
Sodium tiosulfate	Merk	Cat#1065160500
Potassium carbonate	Merk	Cat#1049280500
Formaldehyde solution	J.T. Baker	Cat#02-002-899
Trizma base	Sigma	Cat#T1503
Bovine Serum Albumin	BioWest	Cat#P6154
Bovine Serum Albumin	Sigma Aldrich	Cat#A7030
Re-Blot Plus Strong Solution	Millipore	Cat#2504
RPMI 1640	EuroClone	Cat#ECB9006L
GlutaMAX	Gibco	Cat#35050-038
HEPES buffer 1M	Fisher bioreagents	Cat#BP299-100

(*Continued on next page*)

Continued

REAGENT or RESOURCE	SOURCE	IDENTIFIER
MEM NEAA (100X)	Gibco	Cat#11140-035
Penicillin Streptomycin	Gibco	Cat#15140-122
Sodium pyruvate	Gibco	Cat#11360-039
2-Mercaptoethanol	Gibco	Cat#31350-010
Human AB serum	Sigma Aldrich	Cat#H5667
Dimethyl sulfoxide (DMSO)	MP Bio	Cat#196055
Macrophage serum free media	Gibco	Cat#12065-074
Human GM-CSF	Miltenyi	Cat#130-093-862
HYPure Cell Culture Grade water	Cytiva	Cat#SH30529.02
Lipopolysaccharide O111:B4	Sigma	Cat# L3012
LPS-EB Ultrapure O111:B4	Invivogen	Cat#tlrl-3pelps
ATP	Sigma	Cat#A6419
Nigericin	Sigma	Cat#N7143
Pam3Cys-SKKKK	EMC Microcollections	Cat#L2000
Uric acid	Sigma	Cat#U2625
Low Molecular Weight Poly(I:C)	Invivogen	Cat#tlrl-picw
Poly dA:dT	Invivogen	Cat#tlrl-patn
IFN α/β-receptor	Millipore	Cat#MAB1155
MitoTEMPO	Sigma Aldrich	Cat#SML0703
Z-YVAD-FMK	R&D	Cat#FMK005
UltraPure Ethidium Bromide	Thermo Fischer	Cat#15585011
3-Methyladenine	Calbiochem	Cat#189490
Rapamycin	Millipore	Cat#553210
E−64d	Tocris	Cat#4545
Pepstatin from Streptomyces species, PEPS-RO	Roche	Cat#10253286001
Proteinase K	Qiagen	Cat#19133
PCR direct	Nordic Biosite	Cat#250-302-C
Agarose	Fisher bioreagents	Cat#BP160-100
Midori Green Advance DNA Stain	NIPPON Genetics	Cat#MG 04
Paraformaldehyde (PFA)	Santa Cruz	Cat#sc-281692
Triton X-100	Applichem	Cat#A1388 0500
Urea	Sigma-Aldrich	Cat#U5378-500G
Ammonium bicarbonate (NH4HCO3)	Sigma-Aldrich	Cat#A6141
Benzonase	Santa Cruz Biotechnology	Cat#sc-202391
DL-Dithiothreitol (DTT)	Sigma-Aldrich	Cat#D9779
Iodoacetamide	Acros Organics	Cat#122271000
Trifluoroaceticacid (TFA)	VWR	Cat#85049.051
Acetonitrile	VWR	Cat#83640.320
HPLC grade water	Fischer Scientific	Cat#10505904
Sequencing grade modified trypsin	Promega	Cat#10202904
BioPureSPN PROTO 300 C18 Mini columns	Nets Group	#HUM S18V
Lab-Tek II Chamber Slide w/Cover	Nunc	Cat#154534
DAPI	Sigma-Aldrich	Cat#D9542
Alt-R Cas9 HDR Enhancer	IDT	Cat#1081072
Alt-R Cas9 Nuclease V3	IDT	Cat#1081058
Alt-R Cas9 Elec Enhancer	IDT	Cat#1075916

(*Continued on next page*)

CellPress
OPEN ACCESS

Continued

REAGENT or RESOURCE	SOURCE	IDENTIFIER
Critical commercial assays		
RNeasy Plus Mini Kit (250)	QIAGEN	Cat#74136
iScript	Bio-Rad	Cat#170-8891
Lightcycler 480 SYBR Green Master	Roche	Cat# 4707516001
Ficoll Paque PLUS	Cytiva	Cat#17-1440-03
human IL-1β/IL1F2 DuoSet Elisa	R&D	Cat#DY201
Human total IL-18 DuoSet Elisa	R&D	Cat#DY318-05
human IFN-β DuoSet Elisa	R&D	Cat#DY814-05
human TNF-α DuoSet Elisa	R&D	Cat#DY210
nCounter Custom CodeSet	NanoString Technologies	N/A
MitoSox	Invitrogen	Cat#M36008
Neon transfection system	Invitrogen	Cat#MPK10096
LAL Chromogenic Endotoxin Quantitation kit	Pierce	Cat#88282
Cytotoxicity detection kit	Roche	Cat#11644793001
Autophagy detection kit	Abcam	Cat#139484
Protein Assay Dye	Bio-Rad	Cat#5000006
Amicon Ultra-4 Centrifugal filters 10 kDa	Millipore	Cat#UFC801008
TGX Stain-Free FastCast Acrylamide kit 10%	Bio-Rad	Cat#161-0183
TGX Stain-Free FastCast Acrylamide kit 7.5%	Bio-Rad	Cat#161-0181
Trans-Blot Turbo RTA Transfer Kit	Bio-Rad	Cat#1704272
SureSelectXT human all exon V7 Post-cap	Agilent Technologies	Cat#5191-4005
SureSelect XT Human All Exon V6+UTR kit	Agilent Technologies	Cat# G9707 A-M
Oligonucleotides		
Nontargeting sgRNA	IDT	N/A
NFKB1 mutation sgRNA	IDT	N/A
NFKB1 KO sgRNA	IDT	N/A
Software and algorithms		
Graphpad Prism vs. 9	Graphpad	https://www.graphpad.com/
FlowJo vs. 10.4.2	FlowJo	RRID: SCR_008520
ImageJ	(Zudaire et al., 2011)[69]	https://imagej.nih.gov/ij/
Columbus^{TM} Image Data Storage and Analysis, Version 2.8	PerkinElmer	N/A
Image Lab 6.0	BioRad	RRID: SCR_014210
nSolver 4.0 Analysis Software	nanoString Technologies	RRID: SCR_003420
ICE web tool	SYNTHEGO	https://ice.synthego.com/#/
CRISPR gRNA design	Benchling	https://www.benchling.com/
ANNOVAR	(Wang et al. 2010)[70]	https://annovar.openbioinformatics.org/en/latest/
Burrow-Wheeler Aligner (BWA-mem)	(Li et al. 2010)[71]	https://github.com/lh3/bwa
Genome Analysis ToolKit (GATK)	(Van der Auwera et al. 2013)[72]	https://gatk.broadinstitute.org/hc/en-us
Bioinformatics data analysis and visualization toolkit	(Bedre 2022)[73]	http://doi.org/10.5281/zenodo.3698145
Package 'ímputeLCMD'	(Lazar et al. 2016)[74]	https://cran.r-project.org/web/packages/imputeLCMD/imputeLCMD.pdf
gnomAD	(Chen et al. 2022)[75]	https://gnomad.broadinstitute.org/
Variant prediction tool	Mutation Taster	https://www.mutationtaster.org/
Variant prediction tool	CADD	https://cadd.gs.washington.edu/
SpliceAI, variant prediction tool	(Jaganathan et al. 2019)[76]	https://spliceailookup.broadinstitute.org/
Variant prediction tool	PolyPhen-2	http://genetics.bwh.harvard.edu/pph2/

(*Continued on next page*)

Continued

REAGENT or RESOURCE	SOURCE	IDENTIFIER
Variant prediction tool	SIFT	https://sift.bii.a-star.edu.sg/
Variant prediction tool	SnpEff/SnpSift	https://pcingola.github.io/SnpEff/
Variant prediction tool	InterVar	https://wintervar.wglab.org/
Variant prediction tool	Variant Effect Predictor (VEP 89)	https://www.ensembl.org/info/docs/tools/vep/script/vep_download.html
Deposited data		
Original blots and cytometry histograms	This paper, Mendeley	https://doi.org/10.17632/spgdgkwbtw.1

RESOURCE AVAILABILITY

Lead contact

Further information and requests for resources and reagents should be directed to and will be fulfilled by the lead contact, Kari K. Eklund (kari.eklund@hus.fi).

Materials availability

Cell lines created in this study, p.R157* mutation carrying cell line, *NFKB1*$^{-/+}$, and *NFKB1*$^{-/-}$ are limitedly available through lead contact (kari.eklund@hus.fi) due to lack of an external centralized repository for its distribution and our need to maintain the stock. Cell lines are subjected to technical constrains and completion of a material transfer agreement. This study did not generate new unique reagents, all reagents are commercially available.

Data and code availability

- The original blots and cytometry histograms have been deposited to Mendeley Data and are publicly available as of the date of publication (https://doi.org/10.17632/spgdgkwbtw.1). DOI is listed in the key resources table.
- This publication did not generate original code or structures. Used software are listed with citations in STAR Methods.
- The genome sequencing data, mass spectrometry raw data, and RNA expression data is not publicly available due to the Finnish personal data protection legislation, which limits sending patient data or samples to other research centers without permission of the local Ethics Committee, and because of limitations associated to informed consent. Additional information required to reanalyze the data, including microscopy data, mass spectrometry raw data, and RNA expression data reported in this paper is available from the lead contact (kari.eklund@hus.fi) upon request. Genome sequencing data can only be made available on request. To request access to the data, please contact the lead contact, who will connect you with the responsible researcher at each of the centers. Data will be accessible only with the permission the Ethics Committee of each center where the data were collected. Therefore, the requester must describe the project for which data access is requested, detailing the objectives and data management plan. Data access will be considered for research purposes and non-commercial use only. To ensure patient privacy, access to personally identifiable information or sensitive clinical information (including medical histories) will not be provided, and requests for data access must rigorously adhere to the consent agreements established with study participants. Additional terms and conditions for accessing data by collaborating institutions may apply, as defined by the institutional Ethics Committee.

EXPERIMENTAL MODEL AND STUDY PARTICIPANT DETAILS

Ethics statement

The study was conducted in accordance with the declaration of Helsinki and was approved by the Coordinating Ethics Committee of the Hospital District of Helsinki and Uusimaa (permit no. 138/13/03/00/2013, HUS/404/2020). All patients, or in case of minors their legal guardians, signed an informed consent. All studies were approved by local institutional review and/or Ethics Committees of treating hospitals. All the authors assure the accuracy and completeness of the reported data.

Patient descriptions

In the original **family I,** two brothers (**F1.II-1** and **F1.II-5**) from non-consanguineous parents presented with fever, neutrophilia, deep necrotizing cellulitis with abscesses and high inflammatory markers after elective surgery. The patients' inflammatory symptoms required prolonged intensive care and multiple surgical revisions. No pathogenic bacteria could be cultured from the affected sites. Both patients had normal immunoglobulin levels and no increased susceptibility for infections. Analysis of their whole-exome sequencing (WES) data analysis targeted to 463 genes reported previously to cause Inherited Errors of Immunity disorders (IEI; list available from M.K. or lead contact) identified a pathogenic stop-gain variant of *NFKB1* NM_003998.3:c.469C>T p.R157*, also two likely not

significant variants reported previously to cause IEI disorders were recognized: AGMO (NM_001004320:exon1:c.C49T:p.R17C, dbSNP: rs201065025, nonsynonymous SNV) and NLRP3 (NM_001243133:exon3:c.C674T:p.A225V, dbSNP: rs180177493, nonsynonymous SNV) were found. NGS methods and analyses for **F1.II-1** and **F1.II-5** have been previously described by Kaustio et al.[15] Since that, **F1.II-5** has been successfully treated with anakinra for soft tissue inflammation on the dorsal side of his hand. Several family members are p.R157* variant carriers but the penetrance varies. The mother of the brothers is the first known carrier of the p.R157* variant, she has not suffered of infection susceptibility, autoimmunity or autoinflammation by the age of 91. Also, **F1.II-4** is asymptomatic variant carrier by the age of 66, he has been diagnosed with asthma, but has no history of recurrent infections, autoimmune or autoinflammatory symptoms. Two variant carriers, besides **F1.II-1** and **F1.II-5**, have presented with autoimmune dysregulation. **F1.II-7** had multiple uncomplicated respiratory infections before the age of 7 but has not suffered from frequent infections since. She has had several uneventful operations: appendectomy, and operations for umbilical hernia, protrusion of intervertebral disc, and breast cancer. At the age of 64 she was operated for minor skin lesion, after which there was a suspicion of non-necrotizing wound inflammation, for which she received anakinra (Kineret) 100 mg for three consecutive days. **F1.III-11** is a p.R157* variant carrier and he has been diagnosed with type 1 diabetes. A blood sample from one asymptomatic mutation carrier by the age of 36 **(F1.III-8)**, son of the patient **F1.II-5**, was available for this study.

Index patient of the **family 2 (F2.II-1)** has been previously described by us.[21] The patient is the first child of non-consanguineous parents. He has suffered from alopecia since childhood, and he developed gluteal abscess after intramuscular injection of non-steroidal anti-inflammatory drug for pharyngeal abscess. Shin abrasion at the age of 22 resulted in extensive post-traumatic soft tissue damage and surgical debridement. Two years later, he suffered from recurrent abscesses. He received two dental implants without complications. At the age of 63 he underwent an elective knee arthroplasty, and 3.5 days after the surgery he developed severe neutrophilic leukocytosis (33.7–56.7 x 10^9/L). Regardless of repeated revisions he developed necrotizing fasciitis with high fever and remained septic and hemodynamically unstable. 13 days after arthroplasty his knee prosthesis was extirpated. Neutrophilia peaked on the 20th day after arthroplasty. Extensively repeated blood (n = 16) and deep tissue cultures as well as bacterial PCR and stains remained negative, while superficial cultures grew low counts of *Staphylococcus aureus* and *S. epidermidis*. Despite repeated (n = 9) surgical debridement, he postoperatively quickly developed repeated necrotic deep tissue inflammation along fascial planes, which prompted the use of high-dose oral corticosteroids and above-knee amputation had to be performed. Skin biopsy suggested a neutrophilic dermatosis compatible with pyoderma gangrenosum. Exome sequencing and analysis were performed at the Institute for Molecular Medicine Finland, Helsinki, Finland. The SeqCap EZ MedExome target enrichment kit (Roche) was used for exome capture and sequencing was performed with 101 bp read length on the HiSeq1500 sequencing platform (Illumina). Read mapping and variant calling were performed on genome version GRCh37 using an in-house pipeline and variant annotation was performed with ANNOVAR.[70] Variant filtering was performed for rare (gnomAD,[75] population frequency <0.01), exonic, nonsynonymous, and loss-of-function variants, and analysis targeted to 463 genes reported previously to cause IEI disorders. List available from M.K. or lead contact. WES revealed a heterozygous truncating *NFKB1* mutation NM_003998.3:c.2041C>T p.Q681*. Two other likely benign monoallelic heterozygous mutations were also detected: mutation of mevalonate kinase *MVK* (NM_001114185:c.1129G>A p.V377I, dbSNP: rs28934897, nonsynonymous SNV), which is known to be disease causing when biallelic but not significant as heterozygous), and mutation of *MEFV* (NM_000243:c.2149C>T p.R717C, dbSNP: rs104895192, nonsynonymous SNV). Of his two siblings, **F2.II-2** is asymptomatic carrier of *NFKB1* p.Q681* by middle age.

The proband of **family 3 (F3.II-1)** is a male child of non-consanguineous parents. He presented with symptoms of immune dysregulation at age of 10 when he developed epiglottitis which was treated in hospital. He has had oral aphthae since the age of 35. At age of 44 he developed thrombocytopenia and leukopenia with low NK cell numbers. Bone marrow showed no remarkable changes. At the age of 45 he developed scrotal ulcer which did not respond to antibiotic therapy, and in the same year he also developed recurrent epiglottitis. Common variable immunodeficiency was diagnosed with low immunoglobulin levels and immunoglobulin substitution was initiated. WES revealed a heterozygous truncating frameshift mutation of *NFKB1* NM_003998.3:c.778_779insCTGTC p.G261Vfs*5, no other pathogenic variants fitting to phenotype were found. Exome sequencing and analysis were performed at the Institute for Molecular Medicine Finland, Helsinki, Finland. The SeqCap EZ MedExome target enrichment kit (Roche) was used for exome capture and sequencing was performed with 101 bp read length on the HiSeq1500 sequencing platform (Illumina). Read mapping and variant calling were performed on genome version GRCh37 using an in-house pipeline and variant annotation was performed with ANNOVAR.[70] Variant filtering was performed for rare (gnomAD,[75] population frequency <0.01), exonic, nonsynonymous, and loss-of-function variants, gene variants were analyzed with special focus on genes associated with immunological functions and hematological diseases.

Index patient of **family 4** was a second female child (**F4.II-2**) of non-consanguineous parents. She underwent a vacuum-assisted vaginal delivery due to abnormal cardiotocography. On day P2, she suffered from transient muscular hypotonia. Severe omphalitis due to *Enterococcus faecalis* was diagnosed in addition to progredient swelling behind the right ear, at the site of attachment of the vacuum pump. Despite prompt wide spectrum antibiotic therapies, she continued to suffer from fever with leukocytosis (WBC 25.1x10^9/L, neutrophils 13.8x10^9/L). On day P10, retroauricular swelling extended both extra- and intracranially in brain MRI, with narrow organized subdural hematoma. The next day, purulent, inflamed tissue was extirpated, followed by dura and bone reconstruction. Despite broad spectrum antimicrobial therapy, retroauricular inflammation remained unabated. On P13, revision surgery was performed, and emphysema was drained. Blood cultures every 48–72 h showed no bacterial or fungal growth, and extensive microbiological investigations revealed no pathogens in the wound area after P2. Sutures were removed, while antimicrobial therapy

against yeasts and herpesviruses was initiated on P17. She remained clinically stable with unresponsive purulent deep tissue lesions behind the right ear (6 × 3 cm) and around umbilicus (8 × 4 mm) without high spiking fevers. Pyoderma gangrenosum was diagnosed based on clinical findings, which were supported by biopsy findings with perifollicular inflammation and intradermal abscess formation followed by epidermal and superficial dermal necrosis with an underlying mixed cell infiltrate.

She lacked serum IgA at the birth, but her IgM and IgG concentrations were normal, and her lymphocyte, B and T cell subsets, expression of CD11/CD18, neutrophil oxidative burst after phorbol myristate acetate (PMA) stimulation and CD62L shedding appeared normal or slightly increased. An autoinflammatory disease was suspected, and genetic testing found a monoallelic truncating *de novo NFKB1* mutation NM_003998.3:c.638_641dup p.L215Afs*11. No other pathogenic variants relevant/fitting to phenotype were found.

Trio exome sequencing (for patient and their healthy parents) was performed at the Dr. von Hauner Children's hospital NGS facility (Germany). SureSelect XT Human All Exon V6+UTR kit (Agilent Technologies) was used for exome capture. Sequencing was performed on the NextSeq 500 sequencing platform (Illumina) with 2 x 150 bp read length and 90x average sequencing depth. Read mapping and variant calling were performed on genome version GRCh37 using Burrows-Wheeler Aligner (BWA 0.7.15) and Genome Analysis ToolKit (GATK 3.6).[72] Variant Effect Predictor (VEP 89) and public (e.g., GnomAD,[75] ExAC and GME) and in-house variant frequency databases were utilized in variant filtering and pathogenicity estimation. High-dose intravenous prednisolone therapy was initiated on P19, after which the wound tapered slowly with topical wound care and tacrolimus ointment, resulting in wound closure on P75. Later, her IgA serum levels normalized and vaccine responses against protein and attenuated viruses were tested and found to be normal. She has remained healthy for six years now and has no clinical or laboratory signs of antibody deficiency.

The symptoms of index patient of the **family 5** (**F5.II-3**) begun at age of two when he presented with deep necrotizing ocular cellulitis together with fever, neutrophilia, and increased inflammatory markers. During the next eight years he received several courses of oral glucocorticoids due to pathergy after minimal trauma and developed clinically evident secondary hypercortisolism. No other inflammatory or infectious complications were noted. At the age of 14, he developed acute pneumonia, pleural effusion with abdominal pain, severe neutrophilic leukocytosis (WBC 47x10^9/L) with increased acute phase reaction (P-CRP 333 mg/L). His pleural effusion needed oxygen supplementation despite early intravenous antibiotics. Attempted thoracocentesis resulted in diaphragm damage and stomach puncture. After gastrorrhaphy, he clinically developed posttraumatic pyoderma gangrenosum together with pathergy phenomena at puncture sites. Chest CT scan showed nodules, consolidation, bilateral pleural effusion and ground glass opacity, while his fecal calprotectin levels were normal. Neither hypogammaglobulinemia nor vasculitis-associated autoantibodies were noted, and he responded normally to vaccines and had normal neutrophil oxidative burst after PMA stimulation. T cell subsets were normally distributed. Exome sequencing and analysis was performed at Macrogen (Soul, Korea). SureSelectXT human all exon V7 Post-cap (Agilent) was used for exome capture and sequencing was performed on the Illumina sequencing platform with 150bp read length to 100X average sequencing depth. Read mapping and variant calling were performed on genome version GRCh38 using Burrows-Wheeler Aligner[71] (BWA-mem) and GATK.[72] Variant annotation was performed with SnpEff/SnpSift,[77] ANNOVAR,[70] and Intervar[78] using B-platform software. Variants were filtered for rare (population frequency <0.01, depth of coverage>20x), exonic, nonsynonymous, and loss-of-function variants, and analysis targeted to 54 genes reported previously to cause Inherited Errors of Immunity disorders (gene list available from lead contact). Variant pathogenicity was evaluated according the ACMG guidelines[79] and using the following online tools: Mutation Taster, CADD, spliceAI,[76] SIFT, and PolyPhen-2. In WES, a heterozygous nonsense variant of *NFKB1* NM_003998.3:c.469C>T p.R157* was found and confirmed by sanger sequencing. Also, a heterozygous variant of *ADAR* (ADAR -1:154574541 -rs145588689 -p.Pro236Ala-c.706C>G) was found. High dose intravenous glucocorticoid therapy was tapered gradually after 6 weeks, and long-term subcutaneous canakinumab 4 mg/kg every four weeks was initiated, with complete resolution of stomach fistula, skin pathergy, and skin and pulmonary inflammation in 18 months follow up. The mother of the index patient (**F5.I-2**) carries the same variant, and she has suffered from severe skin disease diagnosed as acne rosacea.

The index patient of **Family 6** (**F6.II-1**) is male of non-consanguineous UK parentage who first presented with intermittent episodes of fever of unknown origin at the age of 28. From 2009 onwards he has suffered from fever episodes and persistently elevated CRP and ESR (40–70 mm/h), microcytic anemia, low grade eosinophilia and monocytosis. No infectious or malignant cause for the fever was identified on extensive investigations including PET-CT and labeled white cell scan, capsule endoscopy and colonoscopy. Progressive splenomegaly to 17 cm was noted associated with mild generalised lymphadenopathy. Multiple lymph node and bone marrow biopsies have shown only reactive changes. There is a family history of mild intermittent febrile illness in his mother, who has not sought medical attention, and his maternal aunt has IgA nephropathy and inflammatory bowel disease. His father and two children are well. In 2011 the proband was hospitalised with cellulitis and myofasciitis of the sternocleidomastoid muscle with cervical adenitis, which settled following surgical debridement and broad-spectrum antibiotic therapy. Diagnostic splenectomy, done to exclude splenic lymphoma in 2014, revealed reactive changes with normal germinal centers. The acute febrile episodes respond rapidly to corticosteroid therapy leading to normalisation of CRP/ESR. Therapy with colchicine and anakinra had no impact on inflammatory markers or frequency of clinical episodes. Immunological workup revealed mild elevation of ANA (1:40 - 1:80) without other autoantibodies, normal immunoglobulin concentrations and B and T cell numbers, although, class-switched memory B cells (CD27+IgM-IgD-) were absent from peripheral blood. Vaccine responses to HiB and pneumococcal vaccine were normal. Suspecting an autoinflammatory disorder, exome sequencing targeted for primary immune disorders (GRID)[80] identified a heterozygous truncating variant of *NFKB1* NM_003998.3:c.1269_1270delAA p.T424Wfs*2 in the proband, which was confirmed by Sanger

sequencing and also identified in his mother. Immunoblotting of PBMC lysates from the proband confirmed reduced expression of p50/105 (Figure S2A), in keeping with *NFKB1* haploinsufficiency. He started on JAK inhibitor therapy during the fall 2023, soon after which his CRP and ESR levels dropped down roughly by 30%–50%.

METHOD DETAILS

Genetic analyses

Whole-genome sequencing (WGS) from venous blood samples of F1.II-1, F1.II-5 (p.R157* variant) were performed at Karolinska Institutet (Sweden) and re-analyzed at the Institute for molecular Medicine Finland (FIMM). The son of F1.II-5, F1.III-2, was Sanger sequenced for p.R157* variant at FIMM. WES for F2.II-1 (p.Q681* variant) and F3.II-1 (p.G261Vfs*5 variant, previously described in[81]) were performed at FIMM and confirmed by Sanger sequencing. For p.L215Afs*11 variant carrier (F4.II-2), WES was performed in Dr. von Hauner Children's hospital NGS facility (Germany). WES for family 5 variant carrier (F5.II-3) was performed at Macrogen (Seoul, South Korea). Genomic testing via targeted panel sequencing for patient F6.II-1 was initially undertaken through the Genomics of Rare Immune Disorders (GRID) research project and confirmed by Sanger sequencing in the clinical diagnostic laboratory. Full details of sequencing for each patient can be found in Patient descriptions.

Culture of human monocyte-derived macrophages

Peripheral blood mononuclear cells (PBMC) derived from variant carriers and healthy controls were isolated from heparinized whole blood by density gradient centrifugation in Ficoll-Paque PLUS (GE Healthcare). After cell plating, monocytes were allowed to adhere for 1 h at 37°C, washed three times with PBS, and differentiated into macrophages for 7 days in Macrophage serum free medium (SFM) supplemented with 100 U/ml penicillin and 100 μg/mL streptomycin (both from Gibco) and 10 ng/mL recombinant human granulocyte macrophage colony stimulating factor (GM-CSF, Miltenyi Biotec) at 37°C. In the experiments controls matched for age- and self-reported gender were processed simultaneously with variant carriers' HMDMs. An additional larger set of HMDMs (n = 10) derived from volunteers that were not matched for the age or the gender of the patients, and were processed separately from patient samples, is shown in Figure 1 panels C and D. HMDM experiments were performed in SFM supplemented with GM-CSF, 100 U/ml penicillin and 100 μg/mL streptomycin.

Culture of THP-1 monocytes

THP1-Dual (InvivoGen, thpd-nfis) cells were cultured at 37°C in RPMI 1640 supplemented with 100 U/ml penicillin, 100 μg/mL streptomycin, 25 mM HEPES (all from Lonza), 2 mM Glutamax and 10% fetal bovine serum (FBS) (both from Gibco). THP-1 experiments were carried out in monocytes to avoid studying the effects of the mutation on monocyte-to-macrophage differentiation that is induced chemically with Phorbol Myristate Acetate. THP-1 experiments were performed in experiment media; RPMI 1640 supplemented with 100 U/ml penicillin, 100 μg/m streptomycin, 25 mM HEPES, 2 mM Glutamax at 37°C.

Stimulation of human monocyte-derived macrophages and THP-1 monocytes

For *in vitro* inflammasome activation, HMDMs were first primed with 1 μg/mL lipopolysaccharides (LPS) from *E.coli* O111:B4 (Sigma) or with 1 μg/mL Pam3Cys-SKKKK (EMC microcollections) for 6 h before NLRP3 inflammasome activation. After priming, NLRP3 inflammasome was activated by addition of 5 mM ATP (Sigma; neutralized stock solution) for 45 min or 0.2 μg/mL monosodium urate crystals (MSU; prepared as previously described[82]) for 16 h. Noncanonical inflammasome was activated in Pam3Cys-SKKKK-primed (1 μg/mL, 6 h) cells by transfection of ultrapure LPS (1 μg/mL, 5h; Invivogen, tlrl3-pelps) using Lipofectamine 2000 (5 μL/mL) after priming.

THP-1 monocytes were stimulated *in vitro* with LPS (1 μg/mL) or Pam3Cys-SKKKK (1 μg/mL), both applied 6 h before stimulation with the NLRP3 inflammasome activator. Incubation times differing from this are indicated separately. In THP-1 monocytes 5 mM ATP for 45 min or 4 μM nigericin for 1 h (Sigma) were used for NLRP3 inflammasome activation. Transfection with low molecular weight Poly(I:C) (Invivogen tlrl-picw) for 2 h or 6 h was used for gene expression analysis in THP-1 monocytes. To activate the noncanonical inflammasome THP-1 monocytes were primed with ultrapure LPS (1 μg/mL, 1 h) and transfected with ultrapure LPS (1 μg/mL, 5 h). To activate AIM2 inflammasome THP-1 monocytes were primed o/n with ultrapure LPS and transfected with poly dA:dT (0.2 μg/mL, 5 h; Invivogen tlrl-patn). Lipofectamine 2000 (5 μL/mL) was used as the transfection reagent. In THP-1 monocytes LPS treatment (1 μg/mL, 6 h 45 min) was used also for inflammasome activation.

HMDMs and THP-1 monocytes were incubated o/n in the presence of IFN α/β-receptor inhibitor (IFNAR2 IgG2a, Millipore MAB1155) 5 μg/mL, and LPS priming and/or activations were carried out on the next day. Specific inhibitor of mitochondrial reactive oxygen species (MitoTEMPO, Sigma SML0703) 200 μM, and NLRP3 inflammasome (MCC950, Invivogen inf-mcc) 10 μM, were applied 1 h before ATP activation (45 min) for HMDMs and THP-1 monocytes. In experiments with THP-1 monocytes the following inhibitors were used: caspase-1/4 inhibitor (Z-YVAD-FMK, R&D), 15 μM, applied 1 h prior to NLRP3 inflammasome activation; autophagy inhibitor 3-MA, 5 mM, applied simultaneously with LPS priming (6 h) and continued during ATP activation (5 mM, 45 min); autolysosomal proteolysis inhibitors E−64d 10 μg/mL in combination with pepstatin A 10 μg/mL (Selleckchem) were applied simultaneously with LPS-activation (4 h or 6 h). Autophagy activator rapamycin, 200 nM, was applied simultaneously with LPS-activation (17 h) and continued during ATP activation (5 mM, 45 min).

THP-1 monocytes devoid of mitochondrial DNA, ρ_0 cells, were generated to study the contribution of oxidized mtDNA and mitochondrial dysfunction in inflammasome activation as previously described.[31] To gain ρ_0 cells, *NFKB1*$^{R157*/R157*}$ and WT THP-1 monocytes were cultured for 16 days in the presence of 100 nM ethidium bromide (Thermo Fischer) and left to rest for one day before activations. THP-1 monocytes and ρ_0 monocytes were activated with LPS (1 μg/mL) for 6 h 45 min, after which the supernatants were collected, and total DNA was extracted. DNA was extracted by suspending 1x10^6 cells to 1:1 PCR Direct (Nordic Biosite) and BPC grade H_2O (Sigma-Aldrich), after which Proteinase K (Qiagen) was added 1:10. Cells were digested o/n at + 55°C, followed by 1 h at + 85°C. Level of mtCOI and *RPLP0* as housekeeping gene (HKG) were analyzed by qPCR.

CRISPR-Cas9-mediated genome editing

NFKB1$^{R157*/R157*}$, *NFKB1*$^{-/-}$ and *NFKB1*$^{-/+}$ were established using the CRISPR-Cas9 system delivered as ribonucleoprotein (RNP) complexes. Before transfection, THP1-Dual monocytes were cultured for 20–24 h in RPMI supplemented with penicillin-streptomycin, 1% sodium puryvate, 1% NEAA, 50 μM b-mercaptoethanol (all from Gibco), and 20% heat inactivated human AB serum (Sigma-Aldrich). The single base mutation p.R157* (located in exon 7) of *NFKB1* was introduced to monocytic THP-1 cell line using RNP complex of Cas9 and 2-part sgRNAs (Table S5; crRNA:tracerRNA-ATTO 550), and p.R157* mutation carrying ssDNA homology directed repair (HDR) template (all from IDT). For creating the KO lines, we induced a 506 base pair deletion in exon 7 by targeting the locus with two sgRNAs (Table S5). All RNP complexes were delivered by electroporation (2 pulses, 10 ms, 1400 V) with Neon transfection system (MPK5000, Invitrogen) in R buffer. After electroporation, the cells that received the p.R157* RNP were cultured for 4 h with 25 μM HDR enhancer (IDT) in RPMI supplemented with penicillin-streptomycin, 1% sodium puryvate, 1% NEAA, 50 μM b-mercaptoethanol and 20% heat inactivated human AB serum, after which the cells were changed to media without HDR enhancer. The cells that received the KO RNP were not treated with HDR enhancer but received the same media as p.R157*RNP transfected cells. One day after transfection the ATTO 550 positive single cells were sorted in 96-wells with BD Influx cell sorter at Biomedicum Flow Cytometry Unit, Helsinki University. Cells were cultured in RPMI supplemented with 100 U/ml penicillin, 100 μg/mL streptomycin, 1% sodium puryvate, 1% NEAA, 50 μM 2-mercaptoethanol and gradually lowered concentrations of heat inactivated human AB serum (from 20% to 2%) at 37°C until there were enough cells to be analyzed for mutation or deletion and for the five most probable off target mutations (Table S5) by Sanger sequencing (Eurofins genomics). All sequences were analyzed with ICE web tool (Synthego).[83]

Due to low editing efficiency in THP-1 monocyte cell line, p.R157* variant carrying cell line was established from a single cell. For experiments with NFKB1$^{-/-}$ and NFKB1$^{-/+}$ four cell lines derived from single cells were used. Five most probable *in silico* predicted off-target mutations were analyzed from all the CRISPR edited cell lines.

Global proteomics analysis

The snap frozen cell pellet was homogenized in 100 μL of 8.0 M urea (#U5378-500G, Sigma Aldrich) in 100 mM ammonium biocarbonate (NH4HCO3, #A6141, Sigma Aldrich) and 1 μL benzonase (Santa Cruz Biotechnology; #sc-202391) was added to each sample. Total protein concentration of the homogenates was measured with Bio-Rad Protein Assay Dye (#5000006, Bio-Rad Laboratories). 20 μg of total protein was taken for global proteomics analysis. The urea concentration was diluted to 1 M with 100 mM Tris-HCl, pH 8.0. Proteins were reduced with 5 mM DL-dithiothreitol (DTT; Sigma-Aldrich, #D9779) for 30 min at 50°C, alkylated with 10 mM iodoacetamide (#122271000, Acros Organics) for 30 min in the dark at room temperature, and trypsin-digested at 37°C for 16 h using Sequencing Grade Modified Trypsin (V5113, Promega).After digestion, samples were acidified with 10% trifluoroacetic acid (TFA, #85049.051, VWR) and desalted with BioPureSPN PROTO 300 C18 Mini columns (#HUM S18V, Nest Group) according to manufacturer's instructions. After desalting the samples were dried in a centrifuge concentrator (Concentrator Plus, Eppendorf). The dried peptides were reconstituted in 30 μL buffer A (0.1% (v/v) TFA, 1% (v/v) acetonitrile (#83640.320, VWR) in HPLC grade water (#10505904, Fisher Scientific).

The desalted samples were analyzed using the Evosep One liquid chromatography system coupled to a hybrid trapped ion mobility quadrupole TOF mass spectrometer (Bruker timsTOF Pro, Bruker Daltonics)[84] via a CaptiveSpray nano-electrospray ion source (Bruker Daltonics). An 8 cm × 150 μm column with 1.5 μm C18 beads (EV1109, Evosep) was used for peptide separation with the 60 samples per day methods (21 min gradient time). Mobile phases A and B were 0.1% formic acid in water and 0.1% formic acid in acetonitrile, respectively. The MS analysis was performed in the positive-ion mode with dia-PASEF method[84,85] with sample optimized data independent analysis (dia) scan parameters. For the DIA analysis the resuspended peptides were further diluted 1:50 in buffer A1 (1% formic acid in HPLC water) and 20 μL was loaded into an Evotip (Evosep) following manufacturer's instructions.

To analyze diaPASEF data, the raw data (.d) were processed with DIA-NN v1.8.1[86,87] utilizing spectral library generated from the UniProt human proteome (UP000005640). During library generation following settings were used, fixed modifications: carbamidomethyl (C); variable modifications: acetyl (protein N-term), oxidation (M); enzyme:Trypsin/P; maximun missed cleavages:1; mass accuracy fixed to 1.5e−05 (MS2) and 1.5e−05 (MS1); Fragment m/z set to 100–1700; peptide length set to 7–30; precursor m/z set to 300–1600; Precursor changes set to 2–4; protein inference not performed. All other settings were left to default.

The input file to further DIA data analysis was the DIA-NN Report.pg_matrix. For data pre-processing an in-house R-script was utilized. Raw intensity values were log2 transformed and median-normalized. Afterward, missing values were imputed using QRILC imputation. Volcano plots were generated with bioinfokit using q-value threshold of 0.05 and log2 intensity fold change thresholds of 1 and -1.

Detection of secreted cytokines, LDH, mtROS, and autophagic vesicles

The mature, cleaved form of IL-1β, TNF, and IFN-β, and release of LDH were detected from cell culture supernatants using Human IL-1β/IL-1F2, TNF, or IFN-β DuoSet ELISA (all from R&D Systems) or cytotoxicity detection kit (Roche) according to the manufacturer's recommendations. ELISA results were analyzed with BMG Labtech Omega FluoStar. For detection of mtROS THP-1 monocytes were washed with PBS and incubated for 15 min in the presence of 5 μM MitoSox (Invitrogen M36008) at 37°C, which was removed by PBS washes prior to flowcytometric analysis (FACS Verse, BD Biosciences, FlowJo software version 10.4.2, Becton Dickinson). Autophagic vesicles were detected from THP-1 monocytes stimulated with LPS (1 μg/mL, 18h) or left untreated using autophagy detection kit (Abcam, ab139484) according to manufacturer's recommendations and staining was analyzed by flow cytometry (Influx, BD Biosciences; analysis was performed in Biomedicum Flow Cytometry Unit, Faculty of Medicine, University of Helsinki).

QUANTITATIVE REAL-TIME PCR

RNA was isolated using RNeasy Plus Mini Kit (Qiagen), followed by cDNA synthesis with iScript kit (Bio-Rad). Quantitative real-time PCR (qPCR) was performed from 10 ng of cDNA per reaction using LightCycler480 SYBR Green I master (Roche) and LightCycler96 instrument (Roche). See Table S5 for the primer sequences. Relative gene expression was calculated using the 2(-ΔΔCt) method using *RPLP0* as HKG.

NANOSTRING ANALYSIS

Direct digital detection of mRNA levels of selected genes from patient PBMC was performed using nCounter Analysis System (NanoString Technologies). The custom gene set contained 50 genes including 45 IFN-regulated, inflammasome-related, JAK/STAT and NF-κB signaling pathway genes. Elongation factor 1-gamma (*EEF1G*), glyceraldehyde-3-phosphate dehydrogenase (*GAPDH*), hypoxanthine-guanine phosphoribosyltransferase (*HPRT1*), ornithine decarboxylase antizyme 1 (*OAZ1*), and tubulin beta class 1 (*TUBB*) were used as HKGs. Sample preparation and analysis were performed as previously described.[88]

Immunofluorescence analysis

The HMDMs were fixed in 48-wells with 4% PFA (Santa Cruz, sc-281692), washed, permeabilized using 0.3% Triton X-100 (Applichem, A1388) in PBS or 0.1% Tween in 1% BSA-PBS, blocked with 5% BSA (Sigma, A7030) in PBS, and incubated over night at +4°C with primary antibodies against p65 (Abcam, ab7970), p50 (Biosite #616702), or p52 (Abcam ab31409) diluted in 0.1% BSA-PBS. After incubation for 1 h at RT with Alexa Fluor 488 -conjugated secondary antibodies (Invitrogen, A11008 and A11029), the nuclei were stained with DAPI and the samples were imaged using Evos (Thermo Fisher) wide field epifluorescence microscope (Biomedicum Imaging Unit, Helsinki University) and analyzed using the ImageJ software.[69] THP-1 monocytes were fixed and stained according to the same protocols, except for the antibodies used for double staining of the cells: the primary antibodies used for p52 (Abcam, ab31409) and p65 (Abcam, ab140751) were detected with secondary antibodies, Alexa 568 (Invitrogen, A11036) for p52 and Alexa Fluor Plus 647 (Invitrogen, A32933) for p65. Samples were imaged using the PerkinElmer Opera Phenix microscope and were analyzed with PerkinElmer Columbus software for cell segmentation and feature extraction (FIMM High Content Imaging and Analysis Unit, Helsinki University).

Immunoblot and immunoprecipitation analysis

HMDMs and THP-1 monocytes were scraped and nonadherent cells were collected by centrifugation into ice-cold cell lysis buffer (Cell Signaling Technology) supplemented with 1x protease inhibitor and 1x phosphatase inhibitor cocktails (both from Roche) at pH 7.0. The lysates were homogenized by bath sonication for 3 × 15 s on ice. Thereafter the whole-cell extracts for immunoblot were mixed with SDS-PAGE Laemmli loading buffer (Biorad) reduced with DTT. For staining with LC3 antibody (Novus Biologicals NB100-2331) THP-1 monocytes were centrifuged and lyzed in sucrose lysis buffer (250 mM Sucrose, 1 mM EDTA, and 1x protease inhibitor).

For immunoprecipitation, whole-cell extracts were prepared using Immunoprecipitation kit (Abcam, ab206996) as per recommended by the manufacturer. Briefly, 200 μg of protein was incubated in nondenaturing lysis buffer and anti-ASC antibody over night at +4°C (1:200, AL177, Adipogen AG-25B0006-C100) followed by 1 h at +4°C incubation with A/G Sepharose beads (30 μL/sample). After incubation beads were washed for 3 times and immunoprecipitates were eluted in 2 x SDS loading buffer reduced with 40 mM DTT.

The proteins were transferred onto PVDF membranes using Transblot Turbo Transfer System and reagents (BioRad), and blocked membranes were incubated over night at +4°C with primary antibodies against NF-κB1 (Cell Signaling Technology #3035), TRIF (TICAM-1, Nordic Biosite #657102), IL-1β (Santa Cruz Biotechnology sc-7884), NLRP3 (AdipoGen AG-20B-0014), SQSTM1 (Adipogen ab56416), LC3 (Novus Biologicals NB100-2331), ASC (AL177, Adipogen AG-25B0006-C100), and GAPDH (Cell Signaling Technology #5174). HRP-conjugated secondary antibodies anti-rabbit P0448 or anti-mouse P0447 (both from Dako) were applied for 1 h at RT, followed by washes and detection using Clarity Western ECL Substrate (BioRad) and ChemiDoc MP Imaging System (BioRad). SDS-PAGE was run using TGX Stain-Free FastCast Acrylamide Kit 10% (BioRad) gels, and stain-free protein imaging was performed for loading control. The used stain free technology enables detection of broad loading range, and it is less affected by biological variation and treatments compared to conventional HKPs.[89] In Figure 1C healthy control 2 sample was applied on both of the blots, to

enable unambiguous comparison of NF-κB1 p50 and p105 protein levels across the blots. Healthy control 2 sample is indicated with an arrowhead in the Figure 1C.

To detect secreted proteins, equal volumes of cell culture media were concentrated using Amicon centrifugal concentrators with 10 kDa cut-off (Millipore), after which their volumes were equalized with the flow through. Equal volumes of concentrated media were run on 10% SDS-PAGE and transferred on PVDF for blotting or silver-stained.[90]

Interaction analysis

Cloning, cell line generation and data analysis was done as previously described.[26] Two cell pellets were processed side-by-side for all studied constructs and both samples were analyzed twice for technical replicates.

QUANTIFICATION AND STATISTICAL ANALYSIS

Statistical significance was assessed using GraphPad Prism vs. 9 software. The used statistical tests are indicated in the figure legends. Statistical analysis for two groups was performed with Wilcoxon test, statistical tests for multiple groups were performed with one-way ANOVA or one-way RM-ANOVA, for groups containing two elements two-way ANOVA or two-way RM-ANOVA were used. Dunnett's, Šidak's or Tukey's testes were used for multiple comparisons. All data is shown as mean ± SD, statistical significance was set at $p < 0.05$ (*$p < 0.05$, **$p < 0.01$, ***$p < 0.001$, ****$p < 0.0001$). *NFKB1* WT and *NFKB1* variant carrying cells were randomly distributed to the treatment groups for *in vitro* stimulations. The number of individuals carrying the same *NFKB1* variant and available for the study was small, and therefore no statistical evaluation was performed in experiments with human primary cells. Separately cultured cells derived from individual variant carriers represent one repeat. In experiments carried out in cell lines, sample size of 3–4 per study group was estimated to yield sufficient statistical power based on preliminary NLRP3 inflammasome stimulations. For cell lines each biological repeat (sample size, n) is an independent experiment performed with cells grown and studied separately. The number of biological repeats is indicated in the figure legends and repeats are shown as separate datapoints in the graphs. Each THP-1 monocyte experiment was carried out with two technical replicates, except for the blots for which technical replicates were pooled; two technical replicates were also used in most of the patient macrophage experiments.

Cell Reports Medicine, Volume *5*

Supplemental information

Truncating *NFKB1* variants cause combined NLRP3 inflammasome activation and type I interferon signaling and predispose to necrotizing fasciitis

Katariina Nurmi, Kristiina Silventoinen, Salla Keskitalo, Kristiina Rajamäki, Vesa-Petteri Kouri, Matias Kinnunen, Sami Jalil, Rocio Maldonado, Kirmo Wartiovaara, Elma Inés Nievas, Silvina Paola Denita-Juárez, Christopher J.A. Duncan, Outi Kuismin, Janna Saarela, Inka Romo, Timi Martelius, Jukka Parantainen, Arzu Beklen, Marcelina Bilicka, Sampsa Matikainen, Dan C. Nordström, Meri Kaustio, Ulla Wartiovaara-Kautto, Outi Kilpivaara, Christoph Klein, Fabian Hauck, Tiina Jahkola, Timo Hautala, Markku Varjosalo, Goncalo Barreto, Mikko R.J. Seppänen, and Kari K. Eklund

Figure S1

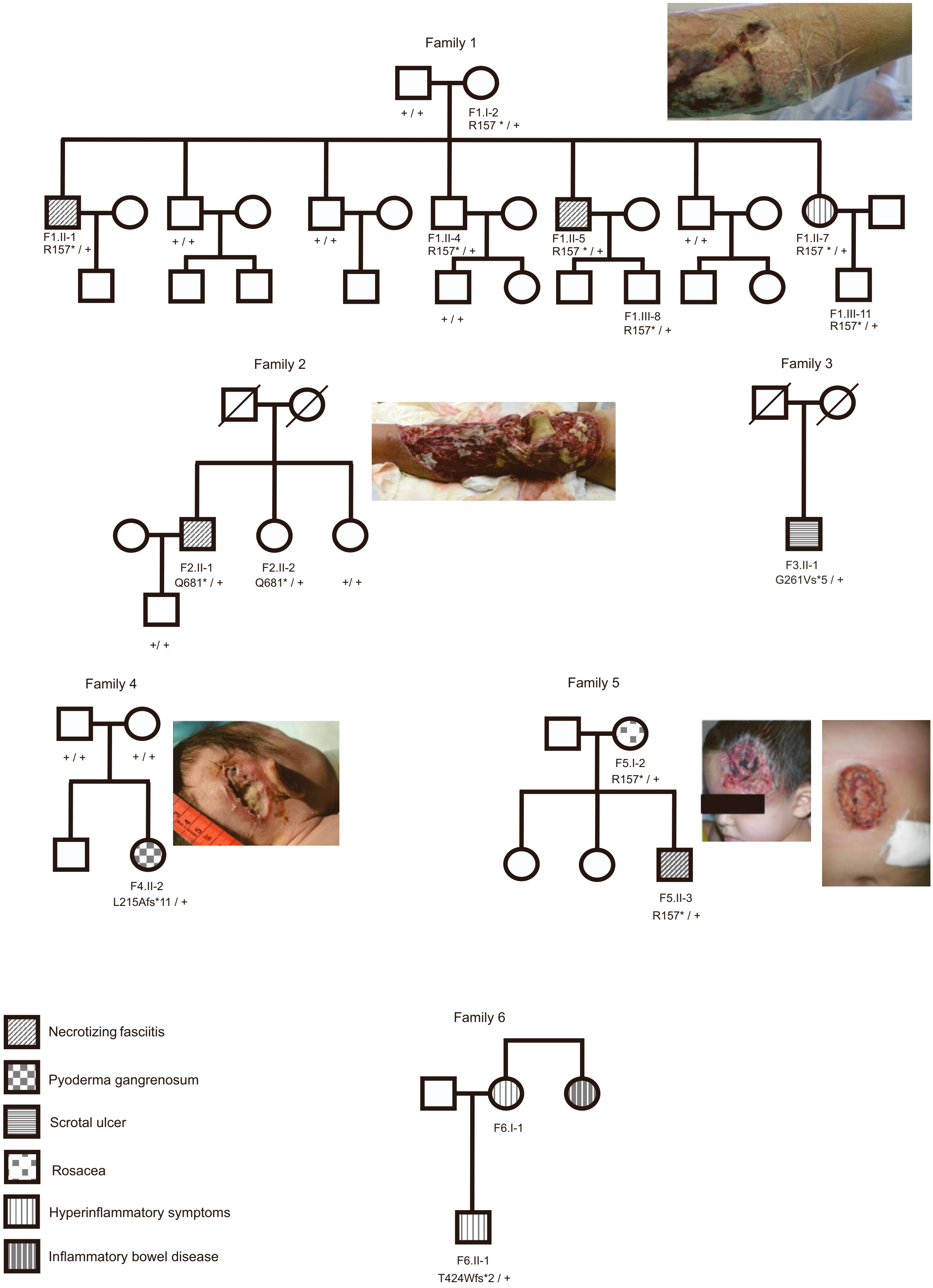

Family 1
+ / +
F1.I-2
R157 * / +
F1.II-1
R157* / +
+ / +
+ / +
F1.II-4
R157* / +
F1.II-5
R157 * / +
+ / +
F1.II-7
R157 * / +
+ / +
F1.III-8
R157* / +
F1.III-11
R157* / +
Family 2
F2.II-1
Q681* / +
F2.II-2
Q681* / +
+/ +
+/ +
Family 3
F3.II-1
G261Vs*5 / +
Family 4
+ / +
+ / +
F4.II-2
L215Afs*11 / +
Family 5
F5.I-2
R157* / +
F5.II-3
R157* / +
Necrotizing fasciitis
Pyoderma gangrenosum
Scrotal ulcer
Rosacea
Hyperinflammatory symptoms
Inflammatory bowel disease
Family 6
F6.I-1
F6.II-1
T424Wfs*2 / +

Figure S1. Pedigrees of *NFKB1* variant carriers and clinical manifestations in patients, related to Figure 1, Tables S1 and S2. Patients with clinical diagnosis are highlighted. +/+, absence of mutation; variant/+, mutation carrier. The clinical manifestation of patients: necrotizing fasciitis in patient F1.II-5; necrotizing fasciitis in patient F2.II-1 which led to leg amputation; retroauricular necrotizing tissue inflammation at the site of attachment of vacuum pump in the newborn (F4.II-2); necrotizing ocular cellulitis and postoperative pyoderma gangrenosum in abdomen in patient F5.II-3.

Figure S2

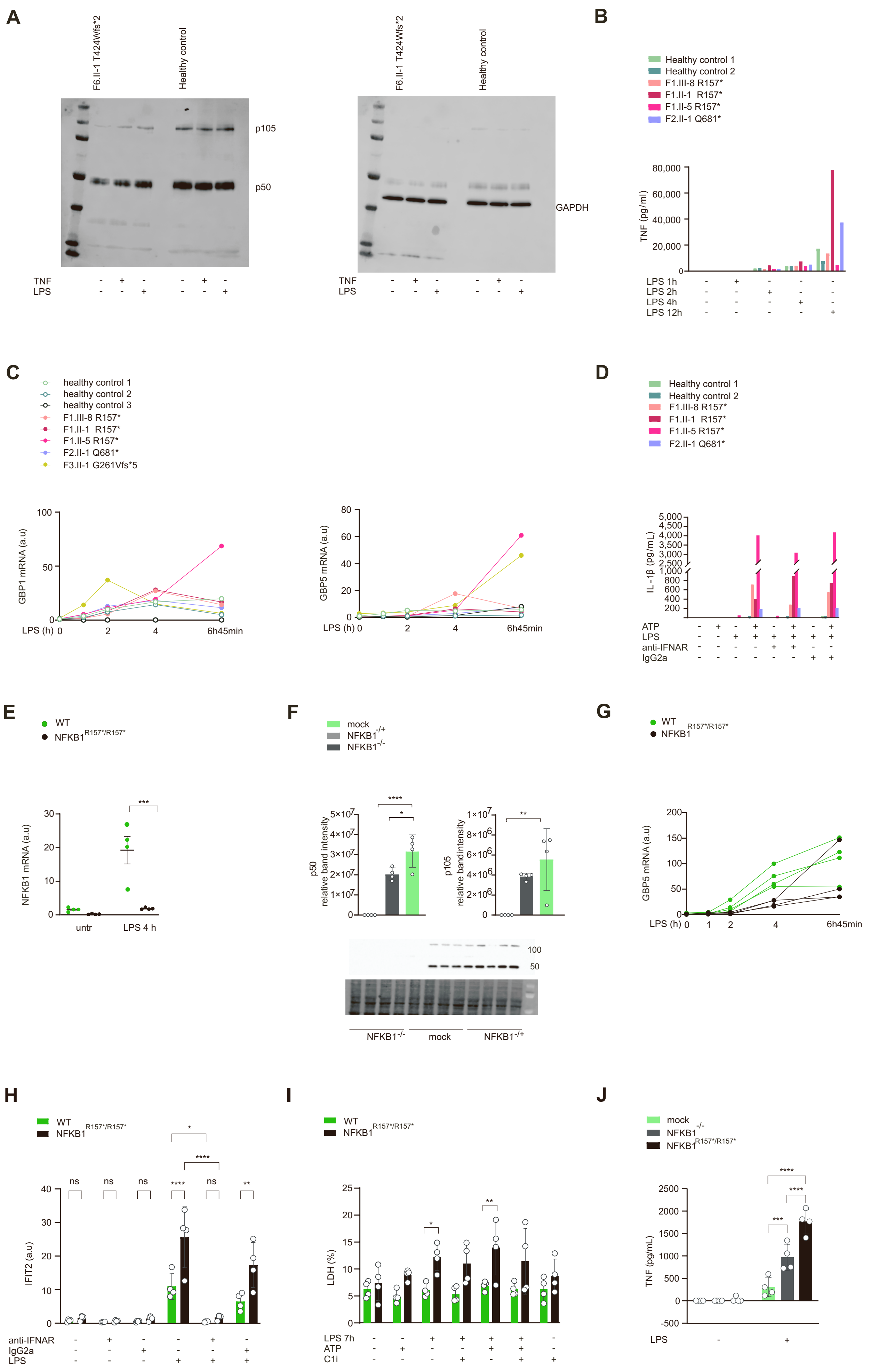
A
F6.II-1 T424Wfs*2
Healthy control
p105
p50
GAPDH
TNF
LPS
B
Healthy control 1
Healthy control 2
F1.III-8 R157*
F1.II-1 R157*
F1.II-5 R157*
F2.II-1 Q681*
TNF (pg/ml)
LPS 1h
LPS 2h
LPS 4h
LPS 12h
C
healthy control 1
healthy control 2
healthy control 3
F1.III-8 R157*
F1.II-1 R157*
F1.II-5 R157*
F2.II-1 Q681*
F3.II-1 G261Vfs*5
GBP1 mRNA (a.u)
GBP5 mRNA (a.u)
LPS (h)
6h45min
D
IL-1β (pg/mL)
ATP
LPS
anti-IFNAR
IgG2a
E
WT
NFKB1R157*/R157*
NFKB1 mRNA (a.u)
untr
LPS 4 h
F
mock
NFKB1-/+
NFKB1-/-
p50 relative band intensity
p105 relative bandintensity
100
50
G
GBP5 mRNA (a.u)
H
IFIT2 (a.u)
I
LPS 7h
C1i
LDH (%)
J
TNF (pg/mL)

Figure S2. IFN-I signaling is required for enhanced expression of selected ISGs but has no effect on enhanced NLRP3 inflammasome activation, related to Figures 1 and 2.

(A) PBMCs derived from p.T424Wfs*2 variant carrier and age and gender matched healthy control were stimulated with LPS (10 ng/mL) and TNF (10 ng/mL) for 16 h and expression of p105/p50 and GAPDH were blotted. **(B)** HMDMs were activated with LPS (1 µg/ml) for indicated times and the secretion of TNF was detected from supernatants by ELISA. **(C)** HMDMs were activated with LPS (1 µg/ml) for indicated times and the expression of *GBP1* and *GBP5* was analyzed by RT-qPCR. **(D)** Anti-IFNAR2 antibody (5 µg/ml) or mouse anti-human IgG2a (mock, 5 µg/ml) were applied 30 min prior to activation of HMDMs with LPS (1 µg/ml, 6 h 45 min) followed by ATP (5 mM, for the last 45 min of the incubation). Mature IL-1β was detected from supernatants by ELISA. **(E)** $NFKB1^{R157*/\ R157*}$ and WT THP-1 monocytes were activated with LPS (1 µg/ml, 4 h) and the expression of *NFKB1* was analyzed by RT-qPCR. 2-way ANOVA followed by Šidak's multiple comparison test. **(F)** The protein expression of NF-κB1 was blotted from the whole cell lysates of untreated $NFKB1^{-/-}$ and $NFKB1^{-/+}$ and mock transfected THP-1 monocytes. Blot and total protein loading shown. 1-way ANOVA followed by Dunnett´s multiple comparison test. **(G)** $NFKB1^{R157*/\ R157*}$ and WT THP-1 monocytes were activated with LPS (1 µg/ml) for indicated times and expression of *GBP5* was analyzed by RT-qPCR. 2-way ANOVA followed by Šidak's multiple comparison test. **(H)** Anti-IFNAR2 antibody (5 µg/ml) or mouse anti human IgG2a (mock, 5 µg/ml) were applied 30 min prior to activation of $NFKB1^{R157*/\ R157*}$ and WT THP-1 monocytes with LPS (1 µg/ml, 6 h 45 min), and the expression of *IFIT2* was detected by RT-qPCR. 2-way ANOVA followed by Šidak's multiple comparison test. **(I)** $NFKB1^{R157*/\ R157*}$ and WT THP-1 monocytes were stimulated with LPS (1 µg/ml, 6 h 45 min) and treated with caspase-1/4 inhibitor (Z-YVAD-FMK, 15 µM) 1 h before ATP (5 µM, for the last 45 min of the incubation). Secretion of LDH was assessed. 2-way ANOVA followed by Šidak's multiple comparison test. **(J)** $NFKB1^{R157*/\ R157*}$, $NFKB1^{-/-}$ and WT THP-1 monocytes were activated with LPS (1 µg/ml) for 6 h 45 min and secreted TNF was detected from supernatants by ELISA. 2-way ANOVA followed by Tukey's multiple comparison test. The data is shown as mean ±SD. (A) 1 variant carrier, 1 control; (B) 4 variant carriers, 2 controls; (C) 5 variant carriers, 3 controls; (D) 4 variant carriers, 2 controls; (E-J) THP-1 n=4.

Figure S3

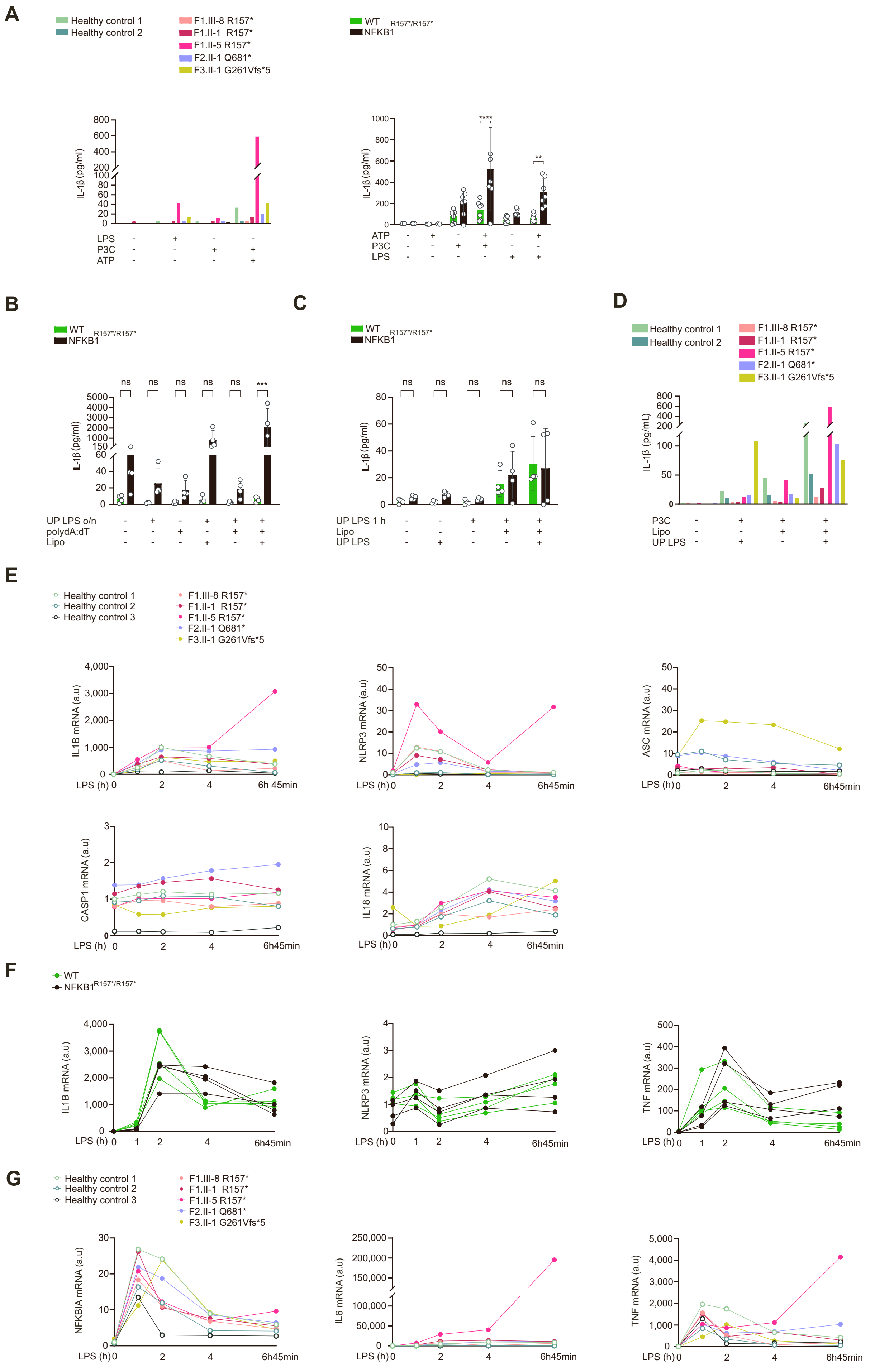

A
Healthy control 1
Healthy control 2
F1.III-8 R157*
F1.II-1 R157*
F1.II-5 R157*
F2.II-1 Q681*
F3.II-1 G261Vfs*5
WT
NFKB1 R157*/R157*
IL-1β (pg/ml)
LPS
P3C
ATP
B
C
D
UP LPS o/n
polydA:dT
Lipo
UP LPS 1 h
UP LPS
IL-1β (pg/mL)
E
Healthy control 3
IL1B mRNA (a.u)
NLRP3 mRNA (a.u)
ASC mRNA (a.u)
CASP1 mRNA (a.u)
IL18 mRNA (a.u)
LPS (h)
6h 45min
6h45min
F
TNF mRNA (a.u)
G
NFKBIA mRNA (a.u)
IL6 mRNA (a.u)

Figure S3. ***NFKB1*** **variants increase AIM2 inflammasome activation but have no effect on activation of the noncanonical inflammasome nor expression of NF-κB-regulated genes, related to Figures 2 and 4. (A)** HMDMs (left panel) and *NFKB1*$^{R157*/ R157*}$ and WT THP-1 monocytes (right panel) were primed with Pam3Cys-SKKKK (1 µg/ml, 6 h 45 min) or LPS (1 µg/ml, 6h 45 min), followed by ATP (5 mM, for the last 45 min of the incubation). Mature IL-1β was detected from supernatants by ELISA. Statistical analysis by 2-way ANOVA followed by Šidak's multiple comparison test. **(B)** *NFKB1*$^{R157*/ R157*}$ and WT THP-1 monocytes were primed o/n with ultrapure LPS (UP-LPS, 1 µg/mL) and transfected with dA:dT (0,2 µg/ml, 5 h) or **(C)** primed with UP-LPS (1 µg/mL, 1 h) and transfected with UP-LPS (1 µg/mL, 5 h). IL-1β secretion was detected from the supernatants by ELISA. 2-way ANOVA followed by Šidak's multiple comparison test. **(D)** HMDMs were primed with Pam3Cys-SKKKK (1 µg/ml, 6 h) after which the cells were transfected with UP-LPS (1 µg/mL, 5 h). IL-1β secretion was detected from the supernatants by ELISA. **(E)** HMDMs were activated with LPS (1 µg/ml) for indicated times and the expression of inflammasome components *IL1B*, *NLRP3, ASC*, *CASP1*, and *IL18* was analyzed by RT-qPCR. **(F)** *NFKB1*$^{R157*/ R157*}$ and WT THP-1 monocytes were activated with LPS (1 µg/ml) for indicated times and expression of *IL1B*, *NLRP3*, and *TNF* was analyzed by RT-qPCR. Statistical analysis by 2-way ANOVA followed by Šidak's multiple comparison test. **(G)** HMDMs were activated with LPS (1 µg/ml) for indicated times and the expression of *NFKBIA*, *IL6*, and *TNF* was analyzed by RT-qPCR. The data is shown as mean ±SD. (A) HMDM 5 variant carriers, 2 controls; THP-1 n=4; (B-C) n=4, (D) 5 variant carriers, 2 controls; (E, G) 5 variant carriers, 3 controls; (F) n=4.

Figure S4

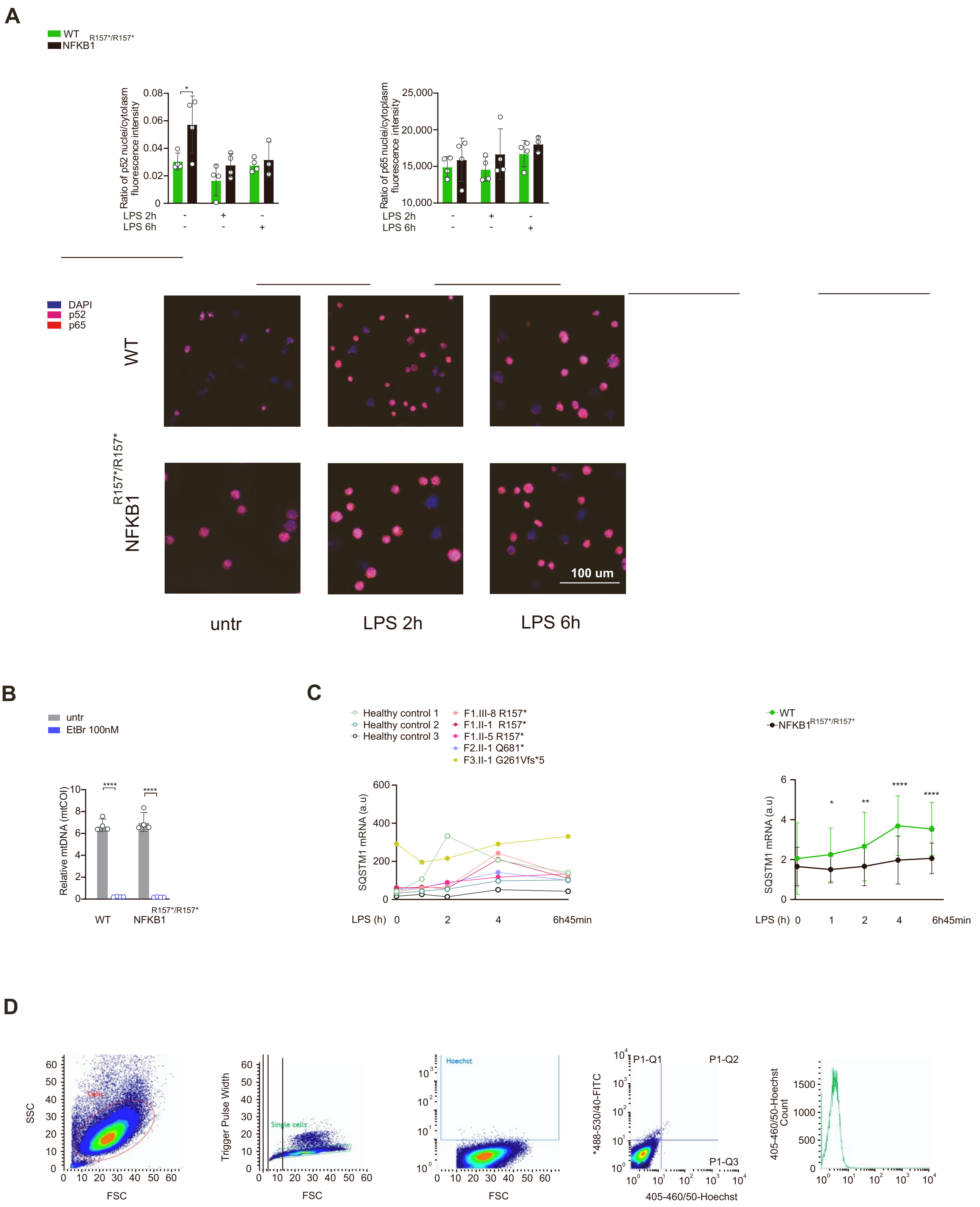

A
WT
NFKB1 R157*/R157*
Ratio of p52 nuclei/cytolasm fluorescence intensity
Ratio of p65 nuclei/cytoplasm fluorescence intensity
LPS 2h
LPS 6h
DAPI
p52
p65
WT
NFKB1 R157*/R157*
100 um
untr
LPS 2h
LPS 6h
B
untr
EtBr 100nM
Relative mtDNA (mtCOI)
WT
NFKB1 R157*/R157*
C
Healthy control 1
Healthy control 2
Healthy control 3
F1.III-8 R157*
F1.II-1 R157*
F1.II-5 R157*
F2.II-1 Q681*
F3.II-1 G261Vfs*5
SQSTM1 mRNA (a.u)
LPS (h)
6h45min
D
SSC
FSC
Trigger Pulse Width
Hoechst
P1-Q1
P1-Q2
P1-Q3
*488-530/40-FITC
405-460/50-Hoechst
405-460/50-Hoechst Count

Figure S4. ***NFKB1*** **variants increase nuclear location of p52 but have no effect on nuclear location of p65, related to Figures 4 and 5. (A)** *NFKB1*$^{R157*/ R157*}$ and WT THP-1 monocytes were activated with LPS (1 µg/mL) for indicated times, and ratios of nuclear-to-cytoplasmic staining for p52 and p65 were determined. Scale bar 100 µm. The data is shown as mean ±SD. **(B)** Total DNA was extracted from resting ρ^0 and conventionally cultured *NFKB1*$^{R157*/ R157*}$ and WT THP-1 monocytes, and levels of mitochondrial DNA were assessed by analyzing the level of mtCOI DNA by RT-qPCR. **(C)** HMDMs (left) and *NFKB1*$^{R157*/ R157*}$ and WT THP-1 (right) monocytes were activated with LPS (1 µg/ml) for indicated times and the expression of *SQSTM1* was analyzed by RT-qPCR. **(D)** Autophagic vesicle detection strategy by flow cytometry (untreated cells depicted). Single cells positive for Hoechst and *488-530/40-FITC were gated. (A-C) 2-way ANOVA followed by Šidak's multiple comparison test. The data is shown as mean ±SD. (A-B) n=4; (C) HMDM 5 variant carriers, 3 controls; THP-1 n=8.

Table S1, *NFKB1* variant carrier information, related to STAR Methods and Figure S1

Family	Family 1	Family 2	Family 3	Family 4	Family 5	Family 6
**Variant carrier**	F1.II-1<br>F1.II-5<br>F1.III-8	F2.II-1	F3.II-1	F4.II-2	F5.II-3	F6.II-1
**Gender**	M, M, M	M	M	F	M	M
**Age of onset**	48 / 28 / N.A.	63	45	newborn	2	30
**Ancestry**	European (Finland)	European (Finland)	European (Finland)	European (Germany)	Latin American (Argentina)	European (UK)
**Genomic variant (GRCh37)**	chr4:103498094, C>T [1]	chr4:103528393, C>T [1]	chr4:103501740, insCTGTC [1]	chr4:103500102 G>GGTGC [1]	chr4:103498094, C>T [1]	Chr4:103516108, delAA [1]
**cDNA change (NM_003998.3)**	c.469C>T	c.2041C>T	c.781_782insTCCTG	c.638_641dup	c.469C>T	c.1269_1270delAA
**Amino acid change**	p.R157*	p.Q681*	p.G261Vfs*5	p.L215Afs*11	p.R157*	p.T424Wfs*2
**Mutation type**	Truncating	Truncating	Truncating	Truncating	Truncating	Truncating
**Inheritance**	AD	AD	AD	*De novo*	AD	AD
**Exon**	7	18	9	8	7	13
**Location of NF or severe tissue inflammation**	(II-1) limb<br>(II-5) limb<br>(III-8) N/A	limb	scrotum	umbilicus, retroaulicular region	orbital region, abdomen	Sternocleido-mastoid muscle
**Blood count**		recurrent leucocytosis	recurrent leucopenia, neutropenia thrombocytopenia, low NK cells	N.A.	N.A.	microcytic anemia, monocytosis, low grade eosinophilia
**Low plasma immunoglobulin**	(II-1) IgG, IgM	-	IgM, IgA, IgG		-	-
**Febrile attacks**	-	-	-	-	x	x
**Pyoderma gangrenosum**	-	-	-	x	x	-
**Other genomic variants with IEI association[2]/ gene name/ variant type/ gnomad freq/ clinical interpretation**	Chr7:15601422,G>A/ AGMO/ nonsynonymous SNV/ 0,0001779/ Likely not significant;<br>Chr16:69727480,A>G/ NFAT5/ nonsynonymous SNV/ 0,00371/ Not significant;<br>Chr1:247587425,C>T/ NLRP3/ nonsynonymous SNV/ 0,0003856/ Likely not significant	Chr1:93303114,A>G/ RPL5/ nonsynonymous SNV/ 0,004748/ Not significant;<br>Chr12:110034320,G>A/ MVK/ nonsynonymous SNV/ 0,001578/ Not significant (as heterozygous);<br>Chr2:48018236,G>T/ MSH6/ nonsynonymous SNV/ 0,0009757/ Not significant;<br>Chr22:37261097,C>A/ NCF4/ nonsynonymous SNV/ 0,00243/ Not significant;<br>Chr8:61693942,->AAAGCA/ CHD7/ nonframeshift insertion/ 0,0062/ Not significant	No other pathogenic variants fitting to phenotype were found	No other pathogenic variants fitting to phenotype were found	Chr1:154574541, C>G/ ADAR/ nonsynonymous SNV/0,00221/conflicting	No other pathogenic variants fitting to phenotype were found
**Other**	N.A.	N.A.	oral aftosis, follicular hyperplasia of lymph nodes, splenomegaly, epiglottitis	N.A.	N.A.	splenomegaly, generalised lymphadenopathy

[1] Variant is not found in GnomAD; [2] all variants are heterozygous.

Table S2 Clinical features of the affected *NFKB1* variant carriers, related to STAR Methods and Figure S1

**Variant carrier**	**F1.II-1**	**F1.II-5**	**F1.III-8**	**F2.II-1**	**F3.II-1**	**F4.II-2**	**F5.II-3**	**F6.II-1**
**Variant**	R157*	R157*	R157*	p.Q681*	p.G261Vfs*5	p.L215Afs*11	p.R157*	p.T424Wfs*2
**Auto-antibodies**	n.a.	n.a.	n.a.	No ANCA antibodies<br>ANA titers: 1) 80; 2) <80<br>No RF or BP180 ab (Pemfigoid)<br>No other skin-directed antibodies (interstitium, basement membrane)	No ANA, no RF	ANA 1:200, no ANCA, no RF	No ANA or ANCA autoantibodies	mild elevation of ANA (40-80) without other autoantibodies
**CRP/ESR during inflammatory episode**	CRP 281 mg/mL	CRP 433 mg/L	n.a.	CRP 352 mg/L	n.a.	CRP 197 mg/L	Pneumonia (age 14 yrs) P-CRP 333 mg /L	Persistently elevated CRP (20-60 mg/L) and ESR (40-70)
**WBC/neutrophils during inflammatory episode**	WBC 46.6 x 10^9 cells/L	WBC 66.8 x 10^9 cells/L	n.a.	33.7-56.7 x 10^9 /L	n.a.	WBC 25.1 x 10^9 / L, neutrophils 13.8 x 10^9 /L	WBC 47 x 10^9 cells /L	WBC 21.81 x 10^9 /L Neutrophils 14.3 x 10^9 /L
**Bacterial cultures during inflammatory episode**	No bacterial or fungal pathogens found in blood cultures or at surgical site.	No bacterial or fungal pathogens found in blood cultures or at surgical site.	n.a.	Blood (n=16), deep tissue cultures, bacterial PCR and stains negative, superficial cultures grew low counts of *Staphylococcus aureus* and S.*epidermidis*	n.a.	No bacterial or fungal pathogens found in blood cultures (every 48-72 h) or in the retroauricular area after P2.	No anaerobic or acid fast bacteria in ulcer culture or intra-abdominal liquid	No bacterial or fungal pathogens found in blood cultures or at surgical site.
**Vaccine responses**	n.a.	n.a.	Response to Pneumovax 4/10	n.a.	Impaired anti-PnP	Normal responses against protein and attenuated viruses	Normal against tetanus toxoid Ab and pneumococcal vaccine	Normal responses to HiB and pneumococcal vaccine
**Neutrophil oxidative burst**	Normal (Ref. 22)	Normal (Ref. 22)	n.a.	n.a.	n.a.	Normal/slightly increased	Normal	n.a.
**CD62L shedding**	n.a.	n.a.	n.a.	n.a.	n.a.	Normal/slightly increased	n.a.	n.a.
**Other**	Slightly reduced marginal zone ($CD27^+IgM^+IgD^+$) and class-switched B-cells ($CD27^+IgM^-IgD^-$) [1]	Slightly reduced class-switched B-cells ($CD27^+IgM^-IgD^-$) [1]	Slightly reduced class-switched B-cells ($CD27^+IgM^-IgD^-$) [1]	n.a.	Splenomeghaly, hypogammaglobulinemia, follicular hyperplasia, epiglottitis (x 2), Scrotal ulcers, complex aftaeae, constant moderate thrombocytopenia and mild neutropenia [2]	Normal/slightly increased expression of CD11/CD18	Slightly reduced class-switched B-cells ($CD27^+IgM^-IgD^-$) and memory B cells ($CD19^+$ $CD27^+$)	No class-switched memory B cells (CD27+IgM-IgD-)

Abbreviations: ANA, antinuclear antibody; ANCA, antineutrophil cytoplasmic antibodies; anti-PnP, anti-pneumococcal polysaccharide.

1. Santaniemi, W., Astrom, P., Glumoff, V., Pernaa, N., Tallgren, E.N., Palosaari, S., Nissinen, A., Kaustio, M., Kuismin, O., Saarela, J., et al. (2023). Inflammation and Neutrophil Oxidative Burst in a Family with NFKB1 p.R157X LOF and Sterile Necrotizing Fasciitis. J Clin Immunol *43*, 1007-1018. 10.1007/s10875-023-01461-3.
2. Tuovinen, E.A., Kuismin, O., Soikkonen, L., Martelius, T., Kaustio, M., Hamalainen, S., Viskari, H., Syrjanen, J., Wartiovaara-Kautto, U., Eklund, K.K., et al. (2023). Long-term follow up of families with pathogenic NFKB1 variants reveals incomplete penetrance and frequent inflammatory sequelae. Clin Immunol *246*, 109181. 10.1016/j.clim.2022.109181.

Table S3. BioID interactome data of NF-κB1 WT and NF-κB1 variants, related to Figure 3.

Averages of affinity purification mass spectrometry streptavidin-hemagglutinin (Strep-HA)-tag normalized log2 transformed interactor values.

Interactor	R157*	G261Vfs*5	Q681*	WT
human NFKB1	2,482	3,867	20,778	25,691
human NFKB2	N.A.	N.A.	5,772	5,336
human TF65	N.A.	N.A.	3,919	3,708
human REL	N.A.	N.A.	2,386	2,155
human RELB	N.A.	N.A.	2,031	1,538
human IKBA	N.A.	N.A.	1,325	0,739
human IKBB	N.A.	N.A.	3,122	2,065
human IKBE	N.A.	N.A.	1,369	1,144
human TNIP2	N.A.	N.A.	1,292	1,098
human TNIP1	N.A.	N.A.	N.A.	0,503
human M3K8	N.A.	N.A.	0,397	0,436
human M1IP1	N.A.	N.A.	0,264	N.A.
human FETUA	0,339	N.A.	N.A.	N.A.
human HACL2	N.A.	N.A.	N.A.	0,573
human FLOT1	N.A.	N.A.	N.A.	0,314

Table S4. Nanostring gene expression data of *NFKB1* variant carriers and healthy controls, related to Figure 4.

Nanostring gene expression raw data, mean of triplicate scans.

Gene	Healthy control	Healthy control	Healthy control	F1.II-1	F1.II-5	F2.II-1	F3.II-1	F4.II-2
CASP1	12436	12694	7832	16040	11190	6763	8036	4228
CASP5	175	199	217	292	144	260	141	146
CASP8	7724	9199	13107	7202	7186	12301	9591	35101
CXCL10	144	163	71	276	80	84	171	44
CXCL9	121	55	71	125	84	154	25	44
IDO1	186	118	71	118	85	67	25	44
IFI27	80	55	76	926	76	67	66	44
IFI44	1625	1804	1911	9528	1966	1886	2768	3114
IFI44L	1640	974	1388	19714	1655	2204	3225	1543
IFI6	603	471	694	2900	808	716	778	645
IFIH1	1017	1055	1691	1820	841	1637	1323	2311
IFIT1	279	390	135	2044	353	311	429	154
IFIT2	1574	353	310	2838	888	515	674	454
IFIT3	160	131	101	793	146	223	117	44
IFNA1	80	55	71	102	76	67	25	44
IFNA2	80	55	71	102	76	67	25	44
IFNB1	80	55	71	102	76	67	25	44
IFNG	220	411	187	508	126	579	169	44
IL18	204	320	394	538	320	405	183	262
IL1A	261	332	170	324	335	124	25	86
IL1B	10709	13652	6368	16767	17297	4895	832	2251
IL6	139	85	281	308	201	231	113	270
ISG15	2348	1620	1863	5319	2131	1809	1953	1215
JAK1	9162	7484	12413	7698	9105	9747	7817	19474
JAK2	5854	5378	5569	10913	5178	5513	6102	4929
JAK3	2275	2924	3502	2229	1958	4803	4960	6861
MX1	3504	3540	4311	19434	5747	6852	7049	6648
NFKB1	1702	1671	1983	1271	1076	1309	1347	3310
NFKB2	2220	2087	7367	2571	1814	5580	2633	13892
NFKBIA	59024	100544	94648	48068	50819	48648	33869	130706
NLRP12	1251	1366	1791	1826	1520	1222	1518	703
NLRP3	3908	3384	5985	4359	4247	5258	2115	7794
OAS1	3755	3822	2609	8276	3106	2556	4061	924
REL	4387	2482	5862	4205	4035	2792	1838	8792
RELA	3683	3363	3508	3302	2987	2972	2645	5054
RELB	1345	905	1943	1122	1121	1198	1266	7061
RSAD2	465	397	340	2709	421	592	711	453
SIGLEC1	226	219	258	5088	305	374	297	196
STAT1	13836	10215	12062	27343	8164	15033	19665	11025
STAT2	1433	1269	1586	2850	1335	1799	2056	1667
STAT3	5876	5101	7759	7221	5967	8044	6588	12998
STAT4	2544	3136	4893	2634	2477	4535	3305	14165
STAT5B	4003	4384	4607	3727	3514	5021	4863	7612
STAT6	6299	6435	14496	8653	7100	16839	10578	14103
USP18	104	115	114	465	111	154	180	174
EEF1G	59729	62326	81358	49047	59706	93062	79289	114768
GAPDH	43788	39829	38315	47927	50881	34234	42730	35782
HPRT1	3038	2892	3190	2910	2857	2923	2615	3933
OAZ1	42929	42178	42991	48993	49551	46007	48330	30167
TUBB	3111	3505	2482	3166	2467	2477	2478	2178

Table S5. Primers used in RT-PCR and PCR analysis, and guides for CRISPR-Cas9 editing, related to STAR Methods.

RT-qPCR primers

Gene	Forward	Reverse
human A20	CTGGGACCATGGCACAACTC	TCGCTGTTTTCCTGCCATTTC
human ASC	TTGGACCTCACCGACAAGC	ATGTCGCGCAGCACGTTA
human CASP1	ATCCCACAATGGGCTCTGTTT	CTCTTTCAGTGGTGGGCATCT
human CASP4	TTGAAAATGGAAGCCACAAGCA	CAAGCTGTACTAATGAAGGTGCTC
human CASP5	CTTCAAGGCCTGGGCTACAC	TCAGCACTGACTCCATATCCCTG
human CXCL10	GCAAGCCAATTTTGTCCACGTGTTG	CAGCCTCTGTGTGGTCCATCCTT
human GBP1	GGAGATTGAAGTGGAACGTGTGA	TCTGCTTTCTTTTTGAAATCCCTCT
human GBP5	GTACTATCGGGAGCCTCGGA	TTTGAATCGCCGCCAACCTT
human IL10	TACGGCGCTGTCATCGATTT	TAGAGTCGCCACCCTGATGT
human IL18	TCAACTCTCTCCTGTGAGAACAAA	GTCCTGGGACACTTCTCTGAAA
human mtCOI1	CGATGGTGCAGCCGCTATTA	ATCATTTACGGGGGAAGGCG
human NFKBIA	CGGACTGCCCTTCACCTCGC	GTATCCGGGTGCTTGGGCGG
human NLRP3	CAACTGCAACCTCACGTCAC	ACGGTCAGCTCAGGCTTTTC
human RPLP0	GAAATCCTGAGTGATGTGCAGC	TCGAACACCTGCTGGATGAC
human SQSTM1	TCGGAGGATCCGAGTGTGAAT	TTTCCCTCCGTGCTCCACAT

PCR primers

Gene name	Forward primer	Reverse primer	Melting temperature
NFKB1 R157*/R157*	ACCCGGTAAGACTTCCTCAT	TGGACCCCTTGGAGGATCAA	60 °C
NFKB1 -/- Exon 7	TGCTCCCACTCCCGTAATCAAA	TGGAAACGGTGGGGTGTTTTTG	62 °C

Off target PCR primers

Off target	Forward	Reverse	Score
1	AGGGGAGATGCTTAGCGTGTTT	AAGGCTTGAGAGAGGAGCACAG	0,88
2	GCGACATTTGCTCATCCTCGAG	TCACTGCGTCTCCTTTCCAACT	0,88
3	TATGGTTGGAGCTGGATCGCAA	CCTTGGTTTGTCACTTATATGCCCC	0,87
4	AGGCGTACAAGGTCAGTGCTTC	CCAGGACAGAAATCAATGCGCT	0,85
5	CCCCAGCTGTAACTGATGGACA	AAGCTACACATGGCTCCAGGTT	0,81

CRISPR-Cas9 guide RNA & HDR donor

Gene name	HDR donor template	sgRNA	
NFKB1 R157*/R157*	TGTTGTTGCTGCTGCTGTTACTGTTTTTTCTCCAGCTTCGCAAACCTGGGTATACTTCATGTGACAAAGAAAAAAGTATTTGAAACTCTGGAAGCATGAATGACTGAAGCGTGTATAAGGGGCTATAATCCTGGACTCTTGGTGCACCCTGACCTTGCCTATTTGCAAGCAGAAGGTGGAGGGGACCGGCAGCTGGGAGG	ACTGGAAGCACGAATGACAG	
**Gene name**		**sgRNA1**	**sgRNA2**
NFKB1 -/-<br>Exon 7		GAGATTTGTATAAAGCATTG	GATGAGGCTGAAAGTTGCTG
